# Reaction enhancement via product scavenging and phase separation-driven pathway canalization

**DOI:** 10.1038/s41467-026-77899-x

**Published:** 2026-09-28

**Authors:** Byunghwa Kang, Heonjoon Lee, Eli Kengmana, Jie Yi Yea, Dong Woo Kim, Rebecca Schulman

**Affiliations:** 1 Institute for Nanobiotechnology, Johns Hopkins University, Baltimore, Maryland USA; 2 Chemistry-Biology Interface Program, Johns Hopkins University, Baltimore, Maryland USA; 3 Department of Biomedical Engineering, Johns Hopkins University, Baltimore, Maryland USA; 4 Department of Chemical and Biomolecular Engineering, Johns Hopkins University, Baltimore, Maryland USA; 5 Department of Computer Science, Johns Hopkins University, Baltimore, Maryland USA

**Keywords:** Biocatalysis, Nanostructures, RNA, Synthetic biology

## Abstract

Targeted biomolecular modification requires recognizing and binding to extended sequence domains, which can limit product dissociation rates and, therefore, catalytic efficiency. Product removal by downstream enzymes or condensates, common in biological catalysis, pushes catalytic reactions toward product formation, but cannot overcome intrinsically low product off-rates. Here, we demonstrate that designed affinity reagents, termed scavengers, can actively displace a product from an enzyme. Designed scavengers for three nucleic acid enzymes increase catalytic turnover up to 39-fold. Additionally, co-localizing enzymes and substrates, but not scavengers, within a biomolecular condensate further enhances >6-fold in catalytic rate by biasing productive substrate-enzyme interaction over inhibitory substrate-scavenger complexation. Scavenging and colocalization can be combined into a single generalizable and programmable framework, multiphase-controlled product scavenging (MCPS). By enabling rapid and exquisitely specific biomolecular modification, MCPS could lead to the development of potent nucleic acid enzyme therapeutics or efficient, controllable pathways for biomolecular modification, cleavage, and synthesis.

## Abstract (web summary)

Catalysts can be slowed when reaction products remain bound to them. Here, authors design scavengers that remove these products and use droplet-like compartments to keep the scavengers away from unreacted reactants, accelerating nucleic acid enzyme reactions by approximately 230-fold.

## Introduction

Catalysis underlies essentially all biological processes from metabolism to gene regulation. However, a ubiquitous limitation of catalysis is product inhibition, in which reaction products decrease catalytic rate and yield^1,2^. For example, 685 of the 747 human metabolic reactions ( ~ 92%) are downregulated by their own products^3,4^. The most prevalent form of product inhibition, competitive inhibition, in which substrate and product compete for the same active site^3,5^, is notoriously difficult to mitigate. Engineering a catalyst to reduce product inhibition is challenging because substrates and products have similar structures and chemistries, so reducing the strength of product binding usually reduces the strength of substrate binding^6^. In practice, it is difficult or impossible to identify changes that neither diminish substrate binding nor decrease overall catalytic efficiency^7–9^.

Product inhibition, therefore, generally must be alleviated by adjusting substrate concentrations or sequestering products. Increasing substrate concentration can enhance catalytic turnover by enabling the substrate to outcompete the product for the catalyst’s active site^10^. However, high substrate concentrations can also lead to substrate inhibition, for example, the binding of multiple substrates to a single catalyst. Substrate inhibition is also common: it is estimated to affect ~ 25% of known enzymes^11^. Increasing substrate concentration can also be impractical because of solubility limits, toxicity, or substrate degradation^12^. Removing products also reduces product inhibition because removing or converting products can shift the equilibrium toward product release by preventing product rebinding, thereby accelerating catalytic turnover^13^. If a product of a catalytic reaction is an intermediate converted in a multi-enzyme pathway, it will be removed through interconversion downstream, driving catalytic flux^14,15^. In cells, products can also be removed or sequestered via compartmentalization, which modulates local molecular concentrations and access^16–18^. Biomolecular condensates, formed by liquid-liquid phase separation (LLPS), for example, can enrich desired reactants while excluding unwanted molecules, thereby accelerating catalytic rates^19^.

However, increasing substrate or decreasing product concentrations alone generally have a limited effect, because in product inhibition, product dissociation is typically rate-limiting. This challenge is particularly evident in synthetic nucleic acid enzymes, including DNAzymes and ribozymes, which are widely used as therapeutics, diagnostics, and molecular biology tools^20^. These catalysts typically demand moderately long targeting domains ( ~ 15–25 nucleotides) to ensure mismatch discrimination and sufficient binding energy^21^. Consequently, product dissociation can be exceedingly slow: the dissociation rate constant for a cleaved product containing only ten base pairs bound to a hammerhead ribozyme has been measured at 2 × 10^−4 ^min^−1^^22^. More broadly, slow product release is observed across diverse enzymatic systems, including CRISPR/Cas9^23^, cellulases^24^, and RuBisCo^25^, where enzymes remain bound to their products from minutes to hours, substantially limiting catalytic turnovers.

**Fig. 1:**
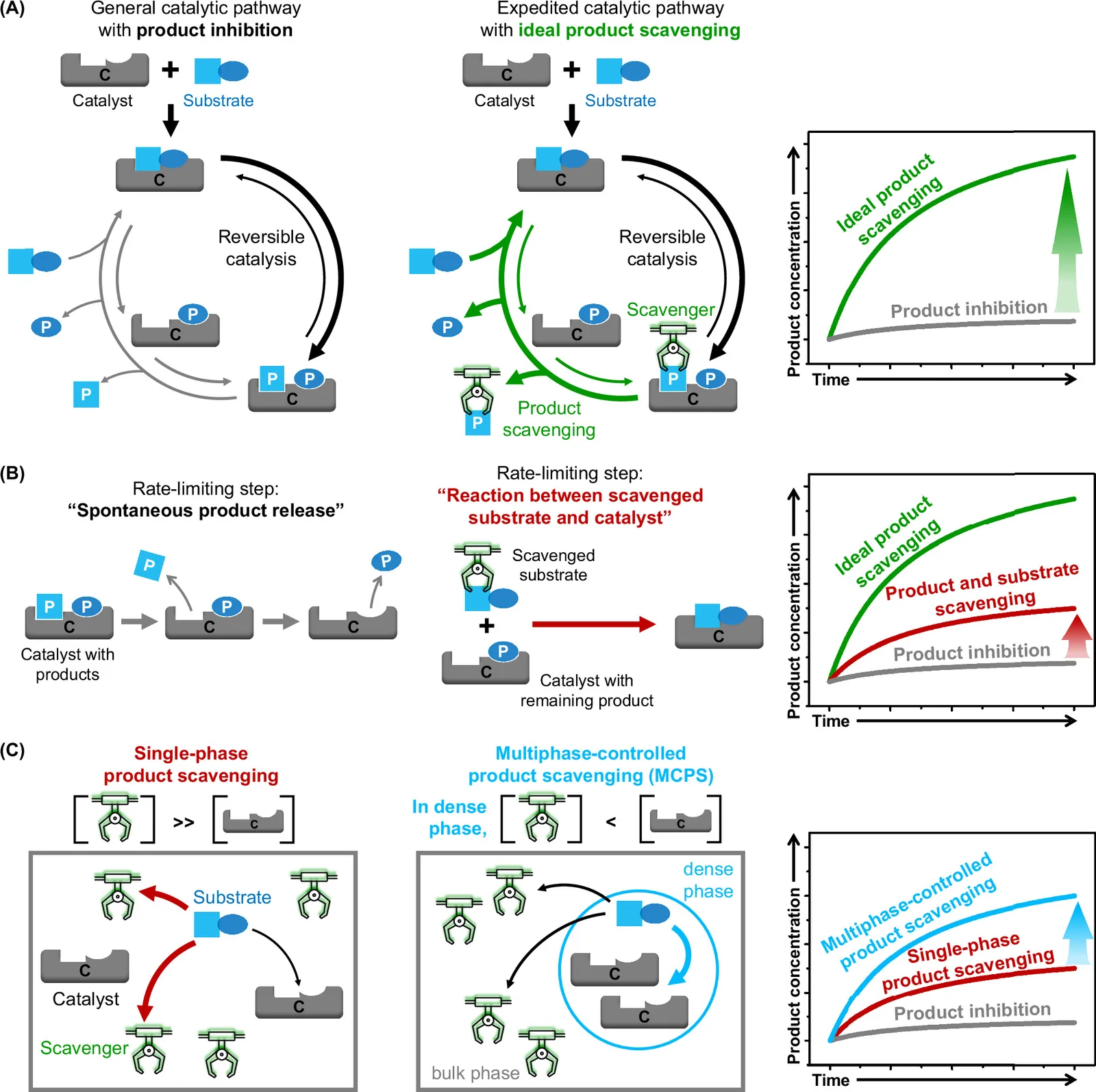
Scavenger- and multiphase-driven control can mitigate product inhibition, enhancing catalytic turnover. **A** In a typical catalytic cycle, the catalyst associates with a specific substrate and performs a reversible catalytic reaction (left scheme). However, the products often impede subsequent catalytic turnover due to their slow dissociation and rapid re-association rates. To mitigate this competitive product inhibition, we propose a controlled catalytic pathway in which a scavenger selectively and rapidly sequesters a reaction product, thereby facilitating turnover by creating a site where a new substrate can bind to the catalyst (right scheme). In the presence of a scavenger, product scavenging removes product dissociation as the rate-limiting step. Product scavenging could, therefore, significantly enhance catalytic turnover rates (right graph). **B** Without scavenging, the dissociation of products from the catalyst is slow, limiting the rate of turnover (left scheme). Scavenging accelerates this step. However, due to structural similarities between the substrate and products, the scavenger may also bind to the substrate, forming a scavenger-substrate complex (right scheme). This unintended interaction can slow the association of the substrate with the catalyst, reducing overall enhancement of catalytic turnover (right graph). **C** A multiphase strategy can circumvent unwanted substrate scavenging. Under multiple-turnover conditions, the substrate’s concentration is typically much higher than that of the catalyst, so that an excess of scavengers is required to effectively sequester all the resulting products (left scheme). However, this excess inevitably leads to unintended substrate scavenging. In a multiphase system in which substrates and catalysts are co-partitioned within the same phase, enriched concentrations of substrate and catalyst promote rapid substrate-catalyst complex formation in one phase. Scavengers are present in excess throughout both phases, allowing them to scavenge the product without being highly concentrated in the substrate and catalyst-rich phase.

Here, we devise a generalizable, specific mechanism to overcome the limitations imposed by slow product dissociation and thereby accelerate catalytic flux. We show that the use of an affinity reagent, a scavenger that dissociates a particular complex of product-enzyme by displacing the product, can overcome slow product dissociation rates, dramatically accelerating catalytic turnover. Once product dissociation is removed as the rate-limiting step by this affinity reagent, compartmentalization even further enhances turnover by maintaining high concentrations of active substrate while sequestering products, yielding a powerful combined strategy for reaction enhancement, multiphase-controlled product scavenging (MCPS) (Fig. 1A–C).

RNA processing reactions experience product inhibition both in vivo and in vitro because cleavage products often retain substantial complementarity to the catalyst and therefore may still dissociate slowly when recognition domains are long. More generally, the severity of product inhibition depends on how product formation changes catalyst-binding affinity; in templated ligation or assembly, for example, it is generally even more severe than for cleavage reactions because the product often binds more strongly to the catalyst than the separate reactants do^26–28^. We demonstrate how MCPS dramatically enhances the catalytic turnover of nucleic acid enzymes, including the hammerhead ribozyme, 10-23 DNAzyme, and hairpin ribozyme for RNA processing reactions. We develop a standard procedure for designing a scavenger complementary to the catalytic product to displace the target product from the catalyst via a strand exchange reaction^29^. This reaction forms a stable product-scavenger complex that prevents product reassociation, thereby mitigating both product inhibition and the reverse reaction that regenerates substrate. We find that introducing scavengers into the reaction mixture can lead to a dramatic increase in turnover frequency; for instance, adding a scavenger for hammerhead ribozyme led to a 39-fold improvement in RNA cleavage rate (from 0.067 to 2.62 h^−1^). However, a new rate-limiting step due to substrate scavenging still limits catalytic rates (Fig. 1B). To circumvent this undesired binding, ribozyme catalysis is co-localized with transcription—a canonical upstream reaction that can supply substrate to ribozymes—within a dense LLPS phase formed by DNA nanostar assemblies (Fig. 1C), achieving 50- to 100-fold enrichment of each reaction component, comparable to biological condensates. MCPS further increases product formation around six-fold over scavenging alone, with a combined enhancement of ~ 230-fold in catalytic rate. In sum, MCPS provides a generalizable and programmable framework by encouraging active product release decoupled from substrate-catalyst recognition, thus providing opportunities for highly effective catalytic conversions demanded in therapeutics, diagnostics, and molecular synthesis^30^.

## Results

### Characterization of ribozyme-mediated RNA processing

**Fig. 2:**
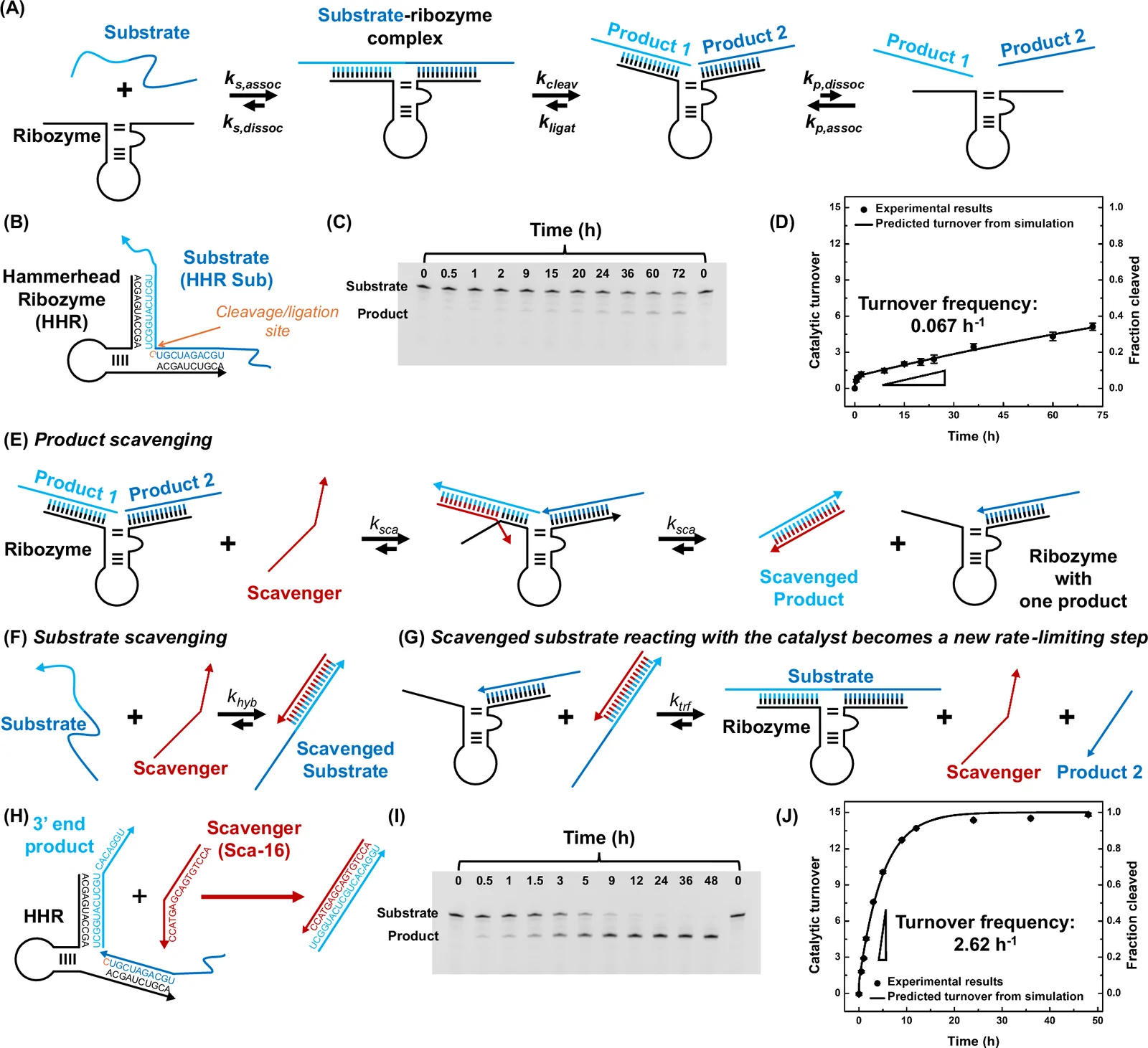
Scavenger-driven enhancement of catalytic turnover of the hammerhead ribozyme reaction. **A** Kinetic mechanism: substrate binding is followed by reversible cleavage; dissociation of both products permits turnover, whereas strong product–ribozyme hybridization causes product inhibition. **B** Secondary structure of HHR-substrate (HHR Sub) complex. Orange arrow indicates cleavage/ligation site. **C** Denaturing-gel time course of FAM-labeled HHR Sub (3 μM) cleavage by HHR (200 nM) over 72 h. **D** HHR turnover and fraction of substrate cleaved. Dots are gel-derived data (including **C**); black line, simulation (Supplementary Note 1). Four biological replicates were used. **E** A product-complementary scavenger selectively removes one cleavage product by strand displacement, yielding a single-product-bound ribozyme that can bind new substrate and a scavenger–product complex to unable to rebind HHR. **F** Because the scavenger also complements the substrate, it forms a substrate–scavenger complex. **G** Scavenger binding masks the substrate’s HHR-binding site, making substrate transfer to the single-product-bound ribozyme rate-limiting. **H** Secondary structures of HHR and both products, and expected Sca-16-mediated displacement of the 3′ product (3’P). **I** Denaturing-gel time course of HHR (200 nM)-catalyzed cleavage of HHR Sub (3 μM) with Sca-16 (20 μM) over 48 h. **J** HHR turnover and fraction of substrate cleaved with Sca-16. Dots are gel-derived data (including **I**); black line, simulation (Supplementary Note 3). Four biological replicates were used. All data are presented as the mean ± SD. Source data are provided as a Source Data file.

To explore scavenger-enhanced catalytic turnover, we chose as model catalysts to study RNA-cleaving ribozymes—naturally occurring RNA enzymes that cleave and ligate RNA substrates^20^ (Fig. 2A). As noted, ribozymes face strong product inhibition because cleaved products are competitive inhibitors of substrate-ribozyme binding^31^. The cleavage rate constants (*k*_*cleav*_, Fig. 2A) of hammerhead ribozymes are generally > 1 min^−1^ under single-turnover conditions^32^. However, when the substrate is in excess, the cleavage rate drops dramatically ( ~ 3 to > 100-fold) if the ribozyme-product recognition domains are stable duplexes, e.g., > 10 base pairs. Yet such long recognition domains that form stable duplexes are often required for accessible and selective recognition of structured RNA targets^33–35^. For example, cleaving a specific RNA transcript by Hepatitis delta virus (HDV) ribozyme requires 15-16 bp recognition^36,37^. Extending the antisense arms of hammerhead ribozymes can be required for efficacy in human cells, particularly for nuclear targets^35,38^. We therefore chose to examine a moderately long recognition system (21 bps) as a deliberately stringent, product-inhibited model relevant to structured-targeting regimes.

To examine this regime experimentally, we designed a hammerhead ribozyme HHR that forms 21 bps with its substrate (HHR Sub) via 10- and 11-nt recognition arms (Fig. 2B). We first confirmed that HHR can cleave its intended target. With excess HHR (2.5 µM) over substrate (0.5 µM), the cleavage kinetics showed a fast reaction rate (2.54 min^−1^) for 57% product formation and following a slow rate (0.14 min^−1^) to generate 43% product (Supplementary Fig. 1). This reaction profile is typical for hammerhead ribozymes, which exhibit conformational heterogeneity^32^. Under multiple-turnover conditions, where HHR Sub (3 µM) was 15 times more abundant than HHR (0.2 µM), product yield was low because turnover reached only ~ 1.6 after 9 h (Fig. 2C, dots in Fig. 2D) due to strong product inhibition.

To identify means to accelerate product formation, we used a standard model of ribozyme-catalyzed reversible RNA cleavage comprising three reactions^32,39^: (1) substrate-ribozyme binding, (2) cleavage of the substrate into two products, and its reverse, the ligation of products to reform the substrate, and (3) product dissociation and subsequent substrate association, yielding a new substrate-ribozyme complex (Fig. 2A, Supplementary Note 1 with Supplementary Fig. 2). All rates but one were drawn from our or previous experimental measurements, with the remaining rate fitted from cleavage kinetics. We used a measured RNA association rate (3.5 × 10^6 ^M^−1^s^−1^)^40^ as a hybridization rate constant (*k*_*s,assoc*_) and the biphasic cleavage rates *k*_*cleav*_ measured for our HHR (2.54 and 0.14 min^−1^). Because ligation is typically 50–150 times slower than cleavage^41,42^, the corresponding ligation rates (*k*_*ligat*_) were set at 0.02*k*_*cleav*_, i.e., 0.052 and 0.0028 min^-1^. The rate of a new substrate-ribozyme complex formation after product dissociation and subsequent substrate association was fitted to our experimental data, resulting in a rate of 6.8 M^−1^s^−1^ (95% CI from 6.37 to 7.23) (Supplementary Fig. 2). This very slow rate is on par with the rate of zero-nt toehold-mediated strand exchange (5.5 M^−1^s^−1^) between RNA strands^40^.

The model shows an initial burst of cleavage, followed by a slow linear rate once turnover is needed (Fig. 2D, line). The first cleavage reaction is completed in the model within 20 min, but turnover reaches only ~ 5 after 3 days. We used the slope of the predicted turnover over time fitted with linear regression, 0.067 h^−1^, as the estimated turnover frequency of HHR (Supplementary Fig. 3), which was over 100 times slower than even the less active form of HHR (0.14 min^−1^). That is, the model shows how product inhibition critically limits the overall rate of product generation.

The model showed how mitigation of product inhibition is required to increase catalytic turnover. Increasing either *k*_*s,assoc*_ or the two cleavage rates ten-fold in the model predicted no or < 2% increases in turnover frequency, respectively (Supplementary Fig. 4A, B). Similarly, eliminating the reverse catalysis, *i.e*., removal of ligation (by setting *k*_*ligat*_ = 0), also increased turnover frequency by < 2% (Supplementary Fig. 4C). By contrast, a tenfold increase in the rate of new substrate-ribozyme formation after cleavage yielded a tenfold increase in turnover frequency (0.66 h^−1^) (Supplementary Fig. 4D).

### Scavenger design and characterization

To expedite the rate-limiting step of product release and subsequent formation of a new substrate-ribozyme complex, we introduced an affinity reagent, termed a scavenger, designed to detach one of the two products from a catalyst through a displacement reaction. Two key design requirements guided this approach.

First, the scavenger should facilitate the release of one of the two products from the catalyst and stably sequester it, i.e., the rate of product displacement facilitated by the scavenger (*product scavenging*) should be much faster than the rate of unassisted product dissociation, and the process should form a stable scavenger-product complex. For nucleic acid hybridization reactions, rapid displacement can be achieved through toehold-mediated strand exchange^29^ (Fig. 2E), which provides a thermodynamic driving force for the reaction. Scavenger-assisted release leaves one of the substrate-binding arms of the ribozyme free, allowing a new substrate to be accessible for the next catalytic turnover. If the scavenger binds to additional nucleotides on the product, it displaces, product-scavenger binding is preferred over product-ribozyme binding, preventing product re-association.

Second, given the structural similarity between the substrate and products, the scavenger will also bind the substrate (*substrate scavenging*; Fig. 2F), slowing down substrate-ribozyme association. Consequently, the association of scavenged substrate and the ribozyme still bound to one product will be the rate-limiting step in turnover (Fig. 2G). An effective scavenger must therefore balance efficient product sequestration with efficient substrate binding to the ribozymes in the presence of the unreleased product and free scavenger.

We applied these considerations to design a scavenger for the HHR using NUPACK (Supplementary Note 2 with Supplementary Fig. 5 and Supplementary Table 2)^43^. First, we designed candidate scavengers complementary to 3’P and checked their thermodynamic stabilities when binding to either 3′P or HHR Sub with those of the corresponding HHR complexes (Supplementary Fig. 5). Second, we assessed whether each candidate could provide a rapid kinetic pathway for removing HHR-bound 3′P through toehold-mediated strand exchange^29,40,44,45^. Candidates were expected to displace 3′P substantially faster than spontaneous substrate-mediated product replacement. Third, we asked whether the full-length substrate (HHR Sub) would still preferentially bind the ribozyme even in the presence of the 5’ end product (5’P) and free candidate scavenger. Assuming sequestration of 3’P by the scavenger, we evaluated whether the HHR:HHR Sub complex remained favored in the presence of 5′P and increasing excesses of scavenger (1-, 10-, and 100-fold; Supplementary Table 2). Based on these analyses, we selected the 16-nt DNA scavenger Sca-16, which is complementary to 3′P and forms a 16-bp DNA:RNA duplex, enabling displacement of 3′P from its 11-bp RNA:RNA duplex with HHR (Fig. 2H and Supplementary Fig. 5)^46–48^. Before cleavage, however, HHR Sub remains resistant to Sca-16-mediated displacement because it forms a 21-bp duplex with HHR. In the simulations, HHR Sub could remain associated with HHR even in the presence of excess Sca-16 and 5′P. We therefore selected Sca-16 as a scavenger that balances efficient product sequestration with retention of HHR:HHR Sub complex formation. We also designed 3′-extended, 5′-truncated, and 3′-truncated variants to examine how scavenger length and orientation affect catalytic turnover (Supplementary Fig. 6).

Sca-16 dramatically accelerated HHR turnover (Fig. 2I). Under conditions requiring multiple turnovers of substrate (0.2 µM HHR and 3 µM HHR Sub), a 72 h incubation without Sca−16 resulted in only five turnovers (Fig. 2D). In contrast, when the same concentrations of HHR and HHR Sub were combined with an excess of Sca-16 (20 µM), the HHR achieved over 7.5 turnovers within just 3 h, representing a > 30-fold increase in turnover rate (Fig. 2I). Both the truncated and extended scavenger variants also significantly enhanced HHR’s catalytic turnover (Supplementary Fig. 6, right), demonstrating the scavenging strategy’s robustness.

To investigate how the scavenger accelerates product formation by the HHR, we extended our ribozyme reaction model to include the two key scavenger-mediated reactions: (1) scavenger-mediated release of the 3’P (product scavenging) and (2) substrate transfer from the scavenger to the ribozyme hybridized to the 5’P (Supplementary Note 3 with Supplementary Figs. 7-9). We assumed a rate constant of 5 × 10^4 ^M^−1^s^−1^ for scavenger-mediated displacement of 3’P (*k*_*sca*_) from the ribozyme based on prior experimental measurements^40,44,45^. Here, other hybridization reactions in the model, such as substrate binding to scavenger (*k*_*hyb*_), were assigned rates of 1 × 10^6 ^M^−1^s^−1^, consistent with measured values^40,44,45^. Varying these rate constants up to three orders of magnitude did not affect HHR’s turnover rate in the model (Supplementary Fig. 8). We then fit the rate (*k*_*trf*_) of substrate transfer from the scavenger to the HHR-5’P complex using the experimentally measured HHR’s turnover rate in the presence of Sca-16 (Fig. 2I-J). The best fit was 2.85 × 10^2 ^M^−1^s^−1^ (95% CI from 2.69 × 10^2^ to 3.00 × 10^2^). The model thus suggests that with the scavenger, product dissociation is no longer the turnover bottleneck; instead, the rate-limiting step becomes transfer of the substrate from the scavenger-bound state to the ribozyme, accompanied by displacement of the remaining 5’P from the HHR-5’P complex. Here, the scavenger does not alter the intrinsic catalytic step of the ribozyme; it introduces additional strand-exchange pathways that redistribute intermediate-state occupancies and reduce effective product inhibition. The full reaction-cycle diagram and kinetic parameters for the scavenger-assisted ribozyme reaction model are provided in Supplementary Fig. 9.

Similar to the model without the scavenger, the new model predicts an initial burst of cleavage, followed by slower but sustained product formation (Fig. 2J, line). However, the slope of the linear region is significantly steeper with scavenging (Supplementary Fig. 10) than without (Supplementary Fig. 3). Linear regression of simulated data yielded a turnover frequency of 2.62 h^−1^, a 39.1-fold improvement in overall cleavage rate vs. without scavenger due to mitigation of product inhibition; although the model includes canonical substrate-assisted product removal, this pathway is substantially slower than scavenger-mediated displacement ( ~ 10⁴-fold), indicating that the scavenger provides the dominant route for relieving product inhibition.

**Fig. 3:**
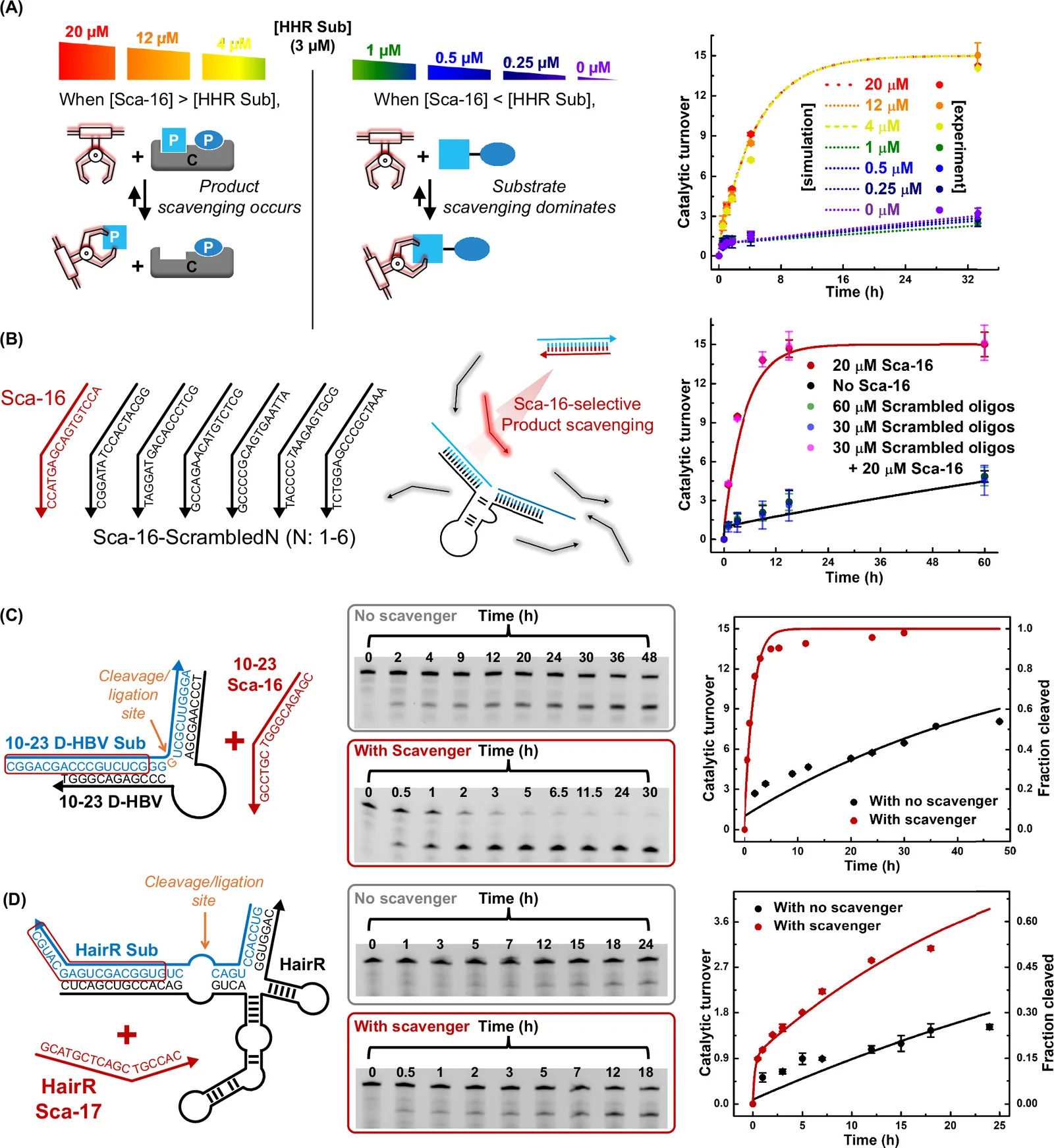
Product scavenging is robust to scavenger concentration, selective, and generalizable. **A** Dependence of catalytic turnover on [Sca-16]. Under multiple-turnover conditions where HHR Sub is in excess of HHR (200 nM), catalytic turnover is effectively accelerated ([Sca-16] > [HHR Sub]). When [HHR Sub] was 3 µM, Sca−16 concentrations of 4, 12, and 20 µM successfully enhanced HHR-mediated RNA cleavage, whereas lower concentrations of Sca−16 (1, 0.5, and 0.25 µM) failed to do so. **B** Sequence-specific acceleration of HHR-mediated cleavage reactions by Sca−16. Six different DNA molecules were generated by randomly scrambling the sequence of Sca−16 using Shuffle DNA (Bioinformatics.org). None of these scrambled strands were able to perform product scavenging to enhance catalytic turnover, demonstrating that Sca−16 exhibits sequence specificity in HHR reactions. The red and black simulation lines correspond to those developed in Fig. 2 with and without scavenger. **C** Scavenger-mediated enhancement of 10–23 DNAzyme-driven cleavage reactions. A 16-nt scavenger (10–23 Sca−16; 20 µM) was designed to specifically sequester one of the two products from 10–23 D-HBV (200 nM). The scavenger effectively alleviated product inhibition, thereby leading to a substantial increase in turnover. [10-23 D-HBV Sub] was 3 µM. The red dotted box denotes the binding domain for 10–23 Sca−16. **D** Scavenger-mediated enhancement of hairpin ribozyme-driven cleavage reactions. A 17-nt scavenger (HairR Sca−17; 20 µM) was designed to target one of the two products of HairR (500 nM), facilitating product removal to increase cleavage turnover. The red dotted box denotes the binding domain for HairR Sca-17. [HairR Sub] was 3 µM. All data are presented as the mean ± SD of three independent biological replicates (*n* = 3). Source data are provided as a Source Data file.

The model also predicts that the scavenger’s concentration only needs to exceed that of the substrate to achieve maximal catalytic acceleration (Fig. 3A and Supplementary Fig. 11). We experimentally confirmed that cleavage was not accelerated at scavenger concentrations below the substrate concentration, whereas ~40-fold acceleration was observed for scavenger concentrations above the substrate concentration (Fig. 3A and Supplementary Fig. 11). Intuitively, when scavenger is limiting, it binds mostly to substrate, preventing the scavenger from accelerating product release. However, when the scavenger is in excess, free scavenger can displace the product.

The scavenger was also selective (Fig. 3B). We designed six molecules with the same ATGC content as Sca-16 but with scrambled sequences that could not displace the target product from the ribozyme (Supplementary Table 3 and Supplementary Fig. 12). When 5 or 10 µM (total concentrations of 30 or 60 µM, respectively) of these six scrambled scavengers were each added, catalytic turnover of the HHR was no higher than without any scavenger. By contrast, adding Sca-16 to a mixture of scrambled scavengers recovers the enhanced catalytic rate (Fig. 3B).

We observed that scavenging is a general strategy applicable to other catalysts with different mechanisms (Fig. 3C, D). The 10-23 DNAzyme is a well-studied RNA-cleaving DNAzyme, with potential applications in gene therapy and diagnostics^49^. We designed a scavenger for a 10-23 DNAzyme that targets the pregenomic RNA of Hepatitis B virus (Accession no. X65257.1) (Fig. 3C, left). Unlike the hammerhead ribozyme, the 10-23 DNAzyme lacks measurable ligation activity^50^. Under multiple-turnover conditions, its turnover frequency was 0.25 h^−1^ (black, Fig. 3C and Supplementary Note 4 with Supplementary Figs. 13-14 and Supplementary Table 4) without scavenger and increased to 6.54 h^−1^ with 20 µM scavenger present (red, Fig. 3C). We then designed a scavenger for a four-way junction hairpin ribozyme (HairR) (Fig. 3D, left), a natural RNA motif involved in RNA processing in vivo^51^. HairR exhibits strong ligation activity, with the internal ligation rate of the ribozyme-bound product state exceeding its internal cleavage rate from the ribozyme-bound substrate state by several- to more than ten-fold^51^. Without scavenger, HairR achieved ~ 1 turnover after 12 h with a turnover frequency of 0.086 h^-1^ (black, Fig. 3D and Supplementary Note 4). With scavenger (HairR Sca-17; 20 µM), the HairR reaction exceeded > 1 turnover within 1 h, and a sustained turnover frequency of 0.22 h^−1^ (red, Fig. 3D).

### Characterization of a multiphase system through LLPS

Although scavengers alleviate product inhibition, excess scavenger can bind substrate (Fig. 3A), which makes substrate transfer to the HHR–5′P complex rate-limiting (Fig. 2G). Consistent with this limitation, our model predicts that eliminating substrate scavenging would increase the HHR turnover frequency to 86.4 h^−1^ (1.44 min^−1^), close to its cleavage rate under single-turnover conditions (1.51 min^_1^) (Supplementary Figs. 1 and  15).

We hypothesized that the inherent tradeoff between product and substrate scavenging might be avoided in an LLPS-induced multiphase system by organizing where substrates, products, catalysts, and scavengers reside (Fig. 1C). If unreacted substrates are localized to a catalyst-enriched phase, the substrate would experience high local catalyst concentrations. Scavenger could be present in both the catalyst-enriched and bulk phases at a lower concentration than the catalyst in the catalyst-enriched phase. In such a system, substrates would preferentially bind to the catalyst in the catalyst-enriched phase, while products could be scavenged by the scavenger and then removed into the bulk phase. Molecules can rapidly shuttle between the dense phase and dilute phases, sustaining this separation. The substantially larger volume of the dilute phase allows it to serve as a reservoir for free scavenger and scavenged products, supporting continued scavenging over multiple reaction cycles^52^.

**Fig. 4:**
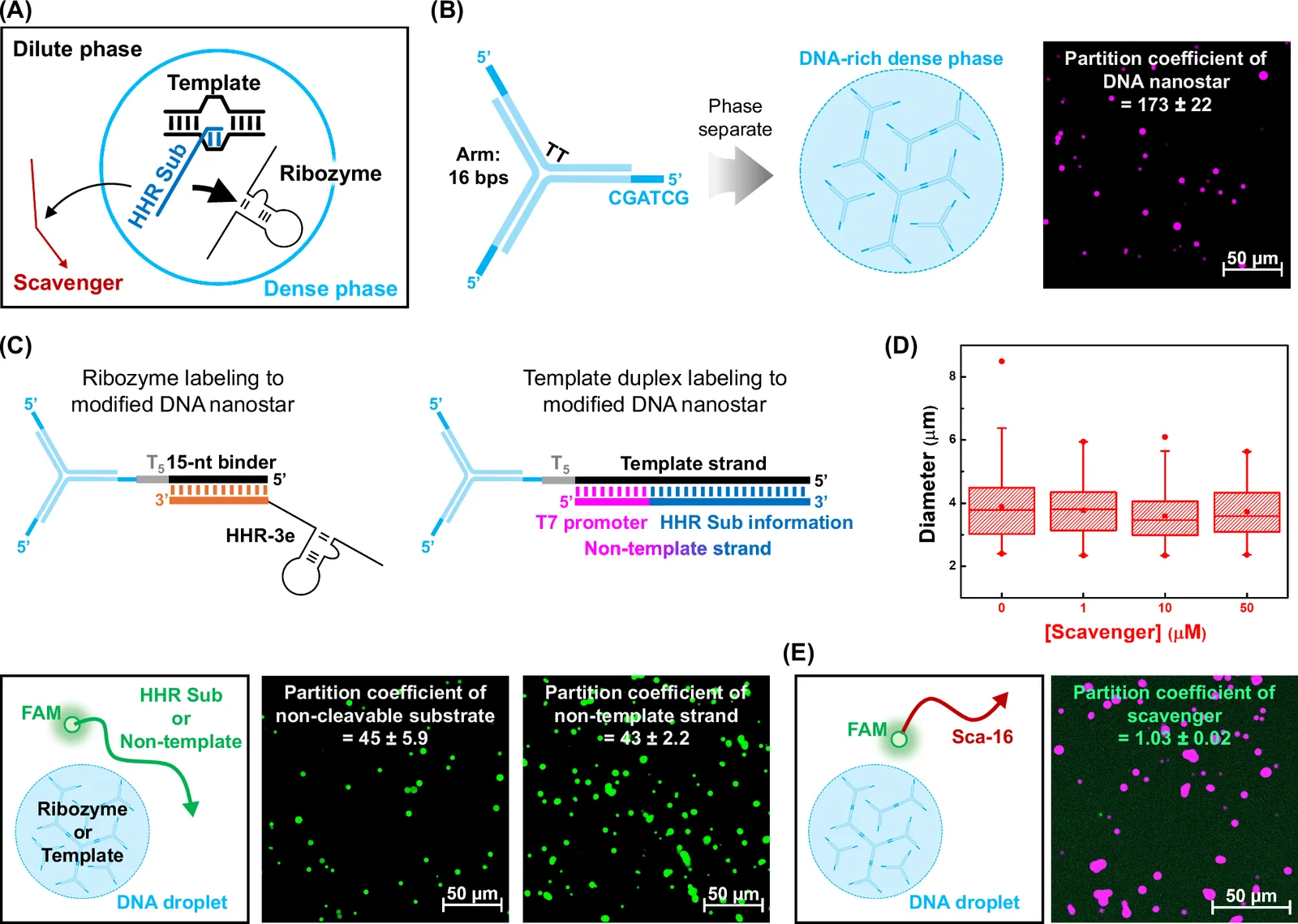
Programmed phase separation can co-localize ribozymes and templates, but not scavengers, within a dense phase. **A** Co-partitioning of the ribozyme with a template where substrates are transcribed within the same phase directs newly transcribed substrates to encounter the ribozyme rather than the scavenger, which is not localized. **B** 3-armed DNA nanostars, consisting of 16-bp arms, two Ts in the central core, and GCTAGC sticky ends for a condensed phase with a partition coefficient exceeding 170. **C** 5’-extension of a DNA nanostar strand for localization of ribozymes or template duplexes. One of the three sticky ends of the DNA nanostar was extended with five Ts as a flexible linker, followed by a binder sequence capable of capturing a target nucleic acid. For ribozyme labeling, the 5’-extended domain contained a 15-nt sequence complementary to the 3’ extended end of HHR. For template duplex attachment, the extended domain included a T7 promoter and a sequence encoding HHR Sub, which, upon hybridization with a non-template strand, formed a stable duplex. When DNA nanostars labeled with ribozymes or templates underwent LLPS, the resulting droplets effectively partitioned FAM-labeled non-cleavable substrates or non-template strands. **D** Effect of scavenger concentration on DNA droplet size. Incubation of Sca-16 with DNA nanostar droplets at varying concentrations (0, 1, 10, 50 µM) showed negligible effects on droplet size (*n* = 91, 107, 112, 82). Box plots show the median (red square) and the 25^th^ and 75^th^ percentiles (lower and upper box bounds, respectively). Whiskers extend to the minimum and maximum values within 1.5 times the interquartile range. Red circles indicate the maximum and minimum values. **E** Measurement of the partition coefficient of Sca-16 (FAM-labeled) in Cy5-labeled DNA nanostar droplets shows homogeneous distribution of Sca-16 between the dense and dilute phases. Source data are provided as a Source Data file.

By adopting LLPS, we hypothesized that co-localization of ribozymes with an upstream substrate-supplying process within a dense phase could guide a new substrate to encounter the localized ribozyme rather than the scavenger, canalizing the reaction pathway toward RNA processing and away from substrate scavenging (Fig. 4A).

To create such a system, we employed designed DNA oligomers (DNA nanostars) that spontaneously phase separate via weak but multivalent hybridization reactions at nanostar ends^53^. The modularity of DNA nanostars allows their modification to include specific domains for localizing desired cargo molecules to the condensed phase. We designed a Y-shaped DNA nanostar with 16-bp duplexed arms, a flexible internal core with two Ts, and a GCTAGC palindromic sticky end (Fig. 4B and Supplementary Fig. 16)^54^. When 5 µM of nanostars (NS), 10% of which were labeled with Cy5 (NS-Cy5), were incubated at 37 °C for 1 h, LLPS occurred, yielding a partition coefficient of 173 ± 22 (Fig. 4B, right)^55,56^. Fluorescence Recovery After Photobleaching (FRAP) experiments showed 50% fluorescence recovery of DNA droplets within 163 ± 5.8 s (Supplementary Fig. 17), confirming rapid droplet mixing and dynamic molecular exchange between phases^57^.

To localize ribozymes within DNA droplets, we modified one DNA nanostar arm to present a 15-nt binding domain at its sticky end (NS-Bind) (Fig. 4C). We then extended the 3’ end of the HHR to present this domain’s complement (HHR-3e) (Fig. 4C, orange). Neither the 3’ extension of HHR nor its hybridization to NS-Bind affected cleavage turnover rates (Supplementary Fig. 18). When NS (4.5 µM), NS-Bind (0.5 µM), and HHR-3e (0.5 µM) were incubated together at 37 °C for 1 h, droplets formed (Supplementary Fig. 19). When a FAM-labeled non-cleavable substrate (NC-HHR Sub) (0.5 µM) (Fig. 4C, bottom left) was added to this mixture, its partition coefficient within the ribozyme-containing DNA droplets was 44.9 ± 5.9 (bottom middle), demonstrating partitioning of the ribozyme to the DNA droplets such that the ribozyme retains its ability to bind the substrate.

As a substrate-supplying process, we chose transcription—a ubiquitous upstream reaction that provides substrates for ribozymes in living cells^20,58^. Localizing transcription templates within droplets can localize RNA’s production and participation in downstream reactions to that phase^54^. We designed an NS-template nanostar with one arm presenting a template strand for substrate transcription (Fig. 4C, blue), including the T7 promoter sequence (magenta) recognized by T7 RNA polymerase (RNAP). A FAM-labeled non-template strand (nonTemplate-FAM) (0.5 µM) was mixed with NS (4.5 µM) and template-labeled nanostar (NS-template) (0.5 µM), resulting in successful localization of non-template with a partition coefficient of 43.1 ± 2.2 (Fig. 4C, bottom right). We also confirmed the co-localization of ribozymes and template duplexes within the same droplets without reducing either’s partition coefficient (Supplementary Fig. 20).

We next verified that scavengers can diffuse between phases without interacting with DNA-rich phases (Fig. 4D, E). Molecules that bind droplet components can act as surfactants, altering droplet size^59^. To test whether scavengers could have this effect, we measured the sizes of Cy5-labeled DNA (4.5 µM NS and 0.5 µM NS-Cy5) droplets at varying Sca-16 concentrations (0, 1, 10, and 50 µM). Average droplet diameters were 3.90 ± 1.06, 3.78 ± 0.82, 3.60 ± 0.79, and 3.73 ± 0.83 µm, respectively (Fig. 4D). The interquartile ranges (IQR) of the diameters, which quantify variation in droplet size, were 1.44, 1.18, 1.05, and 1.23 µm, respectively. Scavengers thus have negligible interactions with DNA droplets. Furthermore, the partition coefficient of FAM-labeled scavenger (Sca-16-FAM) was 1.03 ± 0.02 (Fig. 4E), confirming homogeneous scavenger distribution throughout the multiphase system.

### Multiphase-controlled product scavenging

**Fig. 5:**
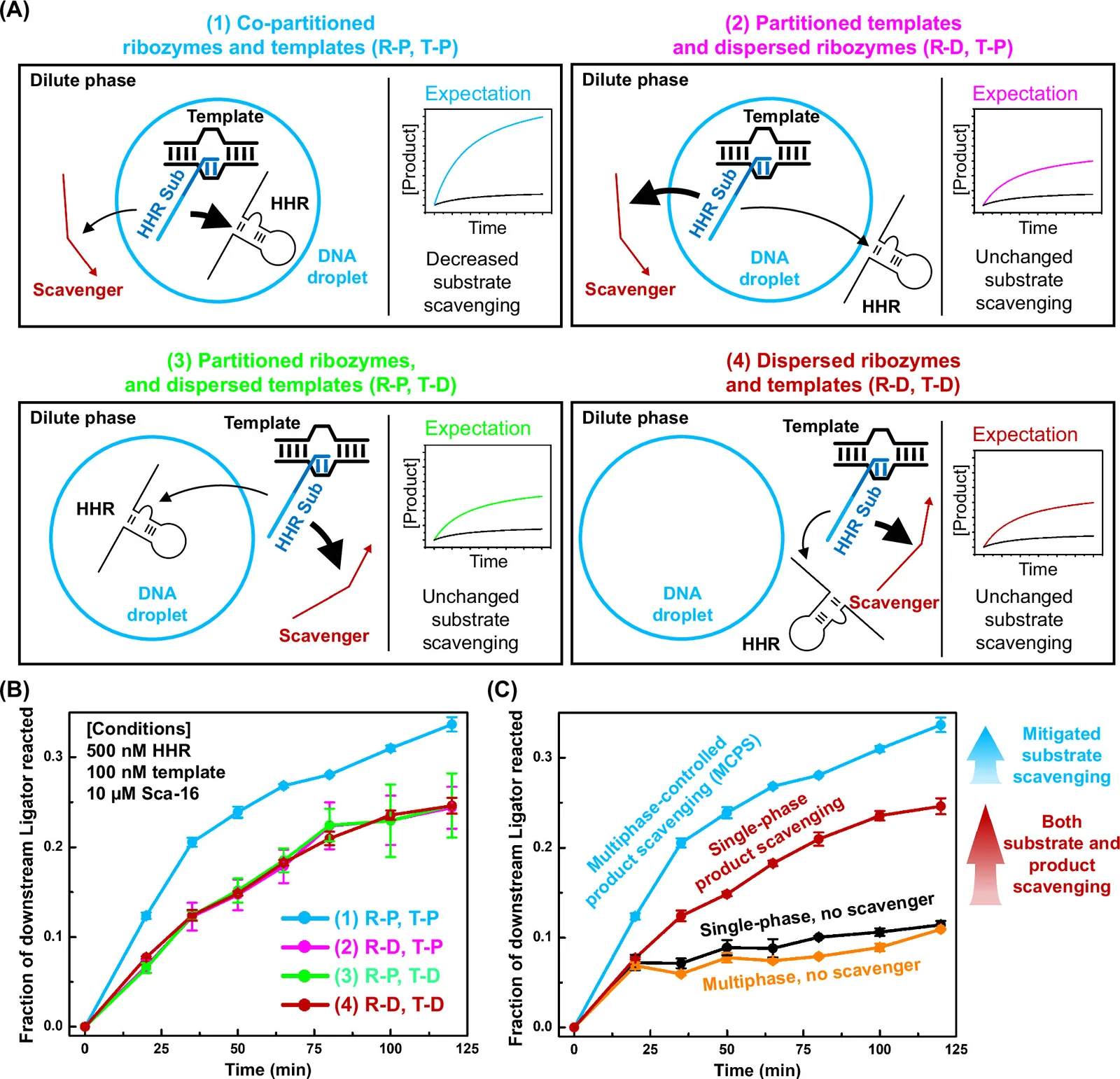
Ribozyme and substrate co-partitioning further speeds up scavenger-accelerated catalysis. **A** Experimental design for evaluating how co-partitioning of ribozymes and templates within the same dense phase enhances the overall rate of substrate cleavage by HHR. The kinetics of cleavage were measured under the four conditions shown. Abbreviations: R-P = ribozyme partitioned, R-D = ribozyme dispersed, T-P = template partitioned, and T-D = template dispersed. **B** Reaction kinetics were analyzed by quantifying a downstream reaction product, one of two cleaved transcripts chemically conjugated to a Ligator in terms of the fraction of Ligator reacted (Supplementary Fig. 21). The fraction of downstream Ligator reacted under four different experimental conditions. Only co-partitioning of ribozymes and templates within DNA droplets significantly increased the fraction of Ligator reacted compared to the well-mixed condition, where ribozymes, templates, and Sca−16s were well-mixed. **C** Measured overall HHR cleaved product production with and without scavenger (Sca−16) and with and without ribozyme-template co-partitioning. Scavenging accelerates HHR-mediated transcript cleavage, and multiphase-driven mitigation of substrate scavenging leads to a further increase in product formation. R-P, T-P experiments were performed in quadruplicate (*n* = 4), whereas all the other experiments were performed in triplicate (*n* = 3). All data are presented as the mean ± SD. Source data are provided as a Source Data file.

To test whether co-localizing substrate production and the ribozyme—but not the scavenger—mitigates substrate scavenging, we compared four spatial arrangements of transcription templates (100 nM) and HHRs (500 nM) (Fig. 5A and Supplementary Table 5). In the first, templates and HHRs were both partitioned within the dense phase (R-P, T-P) (top left, Fig. 5A). In the second, only templates were partitioned (R-D, T-P) (top right), and in the third, only HHRs were partitioned (R-P, T-D) (bottom left). In the last arrangement, both components were dispersed (R-D, T-D) (bottom right). When making ribozymes or templates dispersed in experiments, we replaced ribozyme- or template-labeled nanostars with blunt-end nanostar variants lacking multivalent interactions. Only co-partitioning of the template and HHR was expected to direct newly transcribed substrates preferentially toward locally concentrated HHRs rather than scavengers.

To specifically track ribozyme-driven substrate cleavage, we devised a fluorescence labeling strategy (Supplementary Fig. 21). Because it is difficult to distinguish the catalyzed products from the numerous oligonucleotides in the mixture using conventional gel staining or fluorescence assays, we exploited the 2’-3’ cyclic phosphate present on the ribozyme-catalyzed 5’P^51,60^ but not on solid-phase synthesized or transcribed oligonucleotides. After deactivating ribozymes and RNAPs to halt their reactions, samples were incubated with a designed Ligator complex, comprising a hairpin ligase ribozyme and a FAM-labeled ligation primer. The 5’ hydroxyl group of the ligation primer ligated to the product’s 2’-3’ cyclic phosphate, forming a covalent adduct. The fraction of Ligator reacted then served as a measure of product concentration, and thereby of ribozyme turnover.

Only co-partitioning of templates and ribozymes effectively accelerated ribozyme reactions (Fig. 5B and Supplementary Fig. 22A). After 35 min of transcription initiated by adding RNAP and cleavage, followed by product labeling using the Ligator, the fraction of Ligator reacted was 0.21 ± 0.005 when both reactions were co-localized. However, when either the ribozyme or template was delocalized, the fractions reacted were 0.12 ± 0.015 and 0.12 ± 0.005, respectively, the same as for a well-mixed solution (0.12 ± 0.006).

To confirm that this acceleration stemmed from reduced substrate scavenging rather than altered transcription kinetics, we examined whether template localization altered transcription rates (Supplementary Fig. 23) using a fluorescently labeled DNA reporter that reacts with transcribed HHR Sub via toehold-mediated strand displacement^61^. Under two template and polymerase concentrations (100 nM + 4 units/µl and 10 nM + 1 unit/µl), neither the presence of DNA droplets nor template localization affected transcription rate, indicating that enhanced ribozyme turnover occurred for the same overall amount of substrate not due to greater overall substrate abundance. In addition, we tested whether phase separation can direct reaction pathways by comparing the binding of transcribed HHR Sub to two competing reporter complexes (500 nM) in multiphase and single-phase systems (Supplementary Fig. 24). In the multiphase setup, the HHR-mimicking reporter was co-localized with the transcription template (100 nM), whereas the scavenger-mimicking reporter remained dispersed in the whole solution (Supplementary Fig. 24A). Fluorescence kinetics showed that newly formed HHR Sub preferentially bound the co-partitioned HHR-mimicking reporter in the multiphase system (Supplementary Fig. 24B, C). In contrast, this preference disappeared in the single-phase control (Supplementary Fig. 24D), demonstrating that compartmentalization canalizes the binding reaction pathway by biasing interactions toward spatially co-localized components. Moreover, the result shows that selective partitioning can bias newly generated nucleic acid substrates toward co-localized binding partners and away from dispersed competitors. Thus, the multiphase component of MCPS is not simply a special feature of the HHR catalytic reaction, but a programmable physical mechanism for controlling competing nucleic-acid recognition pathways.

We next verified that greater local concentrations of catalyst than scavenger in the dense phase are required for co-partitioning to accelerate cleavage (Supplementary Fig. 25). If the scavenger concentration approaches or exceeds the catalyst concentration, substrate scavenging will not be mitigated, as the substrate will be as likely to bind to the abundant scavenger as the locally abundant catalyst. Given typical condensed-phase volume fractions of 0.01-0.04 for total nanostar concentrations of micromolar^62^ and the partition coefficient of ribozyme-substrate complex ( ~ 45) (Fig. 4C), the estimated ribozyme concentration within DNA droplets was 8–16 µM. Therefore, at excessive scavenger concentrations (100 µM), locally transcribed substrates would not preferentially bind ribozymes within the dense phase. Consistent with this hypothesis, partitioning of templates and ribozymes provided no advantage in product formation at 100 µM scavenger (Supplementary Fig. 25). This implies that the multiphase system can enhance ribozyme catalysis only when substrate scavenging is circumvented through selective enrichment that presents higher concentrations of ribozyme than scavenger to the substrate.

Lastly, we verified that scavenger- and multiphase-driven reaction pathway canalization works synergistically to accelerate ribozyme catalysis (Fig. 5C and Supplementary Fig. 22B) by comparing the amounts of product formed from transcribed substrate in the presence and absence of both scavenger and template and ribozyme partitioning. After 2 h transcription and ribozyme reactions, both well-mixed and co-partitioned systems lacking scavenger showed similar fractions of Ligator reacted, 0.11 ± 0.004 (black) and 0.11 ± 0.0007 (orange), respectively. Adding a scavenger increased this fraction to 0.25 ± 0.009 (red) in the non-partitioned sample by expediting product dissociation, while further enhancement was achieved (0.34 ± 0.008, blue) when ribozymes and templates were co-partitioned in the presence of scavenger, which permitted product scavenging while effectively mitigating substrate scavenging. To quantitatively analyze the underlying HHR cleavage, we used a calibrated downstream ligation assay to convert ligation yields into concentrations of cleaved transcripts and cleavage turnovers over time (see Supplementary Note 5 with Supplementary Fig. 26). Briefly, after 2 h, the MCPS condition produced ~ 3200 nM cleaved products and left only 387 nM uncleaved transcript, whereas the dispersed condition yielded 1730 nM cleaved product and 1870 nM uncleaved transcript. We used the measured transcription rate (Supplementary Fig. 23) in a simple transcription-coupled kinetic model to estimate *k*_*obs*_ values of 7.75 × 10^−2 ^min^−1^ for MCPS and 1.21 × 10^−2 ^min^−1^ for single-phase product scavenging, corresponding to a 6.5-fold enhancement in cleavage rate. Together, these results show that systemic catalytic pathway control can thus help overcome product inhibition—one of the fundamental challenges in biocatalysis.

## Discussion

We have demonstrated that strong product inhibition along with slow product dissociation—a pervasive rate-limiting process in both biological and engineered catalytic reactions—can be overcome through multiphase-controlled product scavenging (MCPS), which facilitates product liberation and minimizes unwanted substrate-scavenger interactions. MCPS dramatically accelerates catalytic turnover without the need for catalyst engineering, substrate modification, or complex extraction machinery. Our work illustrates how principles from chemical biology, including affinity-driven molecular recognition, programmed nucleic acid hybridization, and designer phase behaviors, can be integrated to reshape the inhibitory landscape of biochemical reactions.

Critically, we observed that MCPS could be generalized to all nucleic acid enzymes tested—despite those enzymes’ differences in chemistry (cleavage versus ligation), structural class, and nucleic acid type. This finding supports the potential broader applicability of product scavenging with nucleic acid catalysis, where predictable base-pairing interactions and thermodynamic analysis can guide scavenger design. Nucleic acid processing is central to many biological and biotechnological systems. Future applications might include additional ribozymes, DNAzymes, and CRISPR-associated nucleic acid enzymes. Extension to protein-enzyme systems could be more difficult because simple base-pairing rules and energy evaluations may not be used reliably, necessitating iterative optimization. Despite this challenge, MCPS could be tested in cases when an inhibitory product can be selectively captured by an available affinity reagent; for instance, SAH-binding RNA aptamers^63^ and UDP-binding aptamers^64^ may be employed as scavenger candidates for SAH-dependent methyltransferase and UDP-producing glycosyltransferase reactions, respectively. More broadly, advances in biotechnology have expanded the available repertoire of diverse affinity reagents, including aptamers and antibodies, with tunable binding affinities^65–67^, that could act as scavengers. For example, product scavenging could be achievable by finding aptamers that bind small peptide products of protease.

MCPS also offers a general design paradigm for regulating enzymatic flux. Rather than altering catalysts or substrates, MCPS shows how controlling where molecules reside and what other species they encounter can overcome fundamental kinetic constraints. Specifically, we found that LLPS-driven molecular enrichment can be sufficient to canalize reaction pathways and bias molecular encounters. The partition coefficients of client components ( ~ 50) observed in our synthetic condensates fall within the range observed in natural biomolecular condensates (2-100)^68^, suggesting such a strategy is broadly applicable. LLPS also inherently provides multiple environments, enabling selective molecular interactions that are difficult to achieve in homogeneous, single-phase solutions, especially when substrates and products differ only subtly. MCPS could therefore be extended to regulate multi-step pathways or dynamically respond to environmental chemical cues. In sum, MCPS represents not only a solution to product inhibition but also a conceptual bridge between synthetic biology, systems chemistry, enzymology, and the emerging biology of LLPS.

Product scavenging is not energetically neutral. The enhanced catalytic flux achieved by MCPS is driven by the favorable free energy of scavenger-product complex formation: the free scavenger acts as a stoichiometric affinity reagent whose binding capacity is consumed as product accumulates, rather than a catalyst. MCPS therefore improves the kinetic efficiency of catalyst turnover, not the thermodynamic efficiency of the overall closed system. This creates an inherent trade-off. More abundant or more strongly binding scavenger more effectively relieves product inhibition, but also increases reagent consumption and can promote competing substrate scavenging, consistent with our observation that efficient acceleration requires a scavenger in excess over substrate. In practice, however, this trade-off can be advantageous because MCPS uses a DNA affinity reagent to accelerate a process catalyzed by any nucleic acid enzymes, including ribozymes. DNA oligonucleotides are generally less expensive to synthesize, easier to handle, and more stable during storage than RNA enzymes^69^. Thus, even though the scavenger is consumed stoichiometrically, using an inexpensive and stable DNA strand to improve the productive turnover of a more costly and less stable RNA catalyst can provide a practical and potentially economical route to faster RNA-catalyzed reactions. This feature is particularly relevant for diagnostic or analytical applications, where rapid and sensitive signal generation is often prioritized^70^, and where improving the turnover of the catalytic RNA component may be preferable to increasing the amount of ribozyme required.

A complementary strategy was recently demonstrated for DNA-templated assembly^28^, in which product formation creates an internal toehold that allows a fuel strand to selectively displace the completed product from its template. Because this recognition motif is generated only after assembly, the fuel avoids substantial binding to the separate reactants, thereby preventing substrate-scavenging. In cleavage reactions, however, the product sequence is already contained within the uncleaved substrate, making analogous product-selective recognition less straightforward. MCPS mitigates this limitation through spatial control. Future approaches could exploit cleavage-generated termini, structures, or newly exposed recognition sites to achieve product-gated scavenging.

## Methods

### Reagents and materials

All oligonucleotides were synthesized by Integrated DNA Technologies (IDT) (Coralville, IA) if not otherwise mentioned (Supplementary Table 1). Tris buffer (pH 7.9) was prepared by using Trizma Base (T6791) and Trizma HCl (T6666) purchased from Sigma-Aldrich (St. Louis, MO). Magnesium acetate tetrahydrate (M0631), formamide (F7503), N,N,N’,N’-tetramethylethylenediamine (TEMED) (T9381) and dithiothreitol (DTT) (43819) were purchased from Sigma-Aldrich. Ribonucleotide triphosphates (R0481) solution, T7 RNA polymerase (EP0113), empty gel cassettes (NC2010), 10X TBE buffer (15581044), and urea (29700) were purchased from Thermo Scientific (Waltham, MA). Ammonium persulfate (193988) was purchased from MP Biomedicals (Irvine, CA). SequaGel ureagel 29:1 (EC-829) was purchased from National Diagnostics (Charlotte, NC). Inorganic pyrophosphatase (M2403S) was purchased from New England Biolabs (Ipswich, MA). All chemicals are used in experiments with no further purification.

### Catalyzed RNA cleavage reactions

To investigate cleavage reactions mediated by the hammerhead ribozyme, we prepared a reaction mixture containing 10 µM ribozyme solution (0.5 µl), 4X reaction and condensation buffer (4X RC buffer; 110 mM magnesium acetate, rNTPs with 10 mM each ribonucleotide triphosphate, 100 mM Tris-HCl, 4 mM DTT) (6.25 µl), varying concentrations of a scavenger (5 µl), and 12.5 µl of deionized water. To initiate the ribozymatic cleavage reaction, 100 µM HHR Sub (0.75 µl) was added to the mixture, resulting in a final reaction volume of 25 µl. The reaction mixture was incubated at 37 °C, and 2 µl samples were collected at designated time points. To terminate ribozyme-driven cleavage after sampling, each sample was immediately mixed with > 99% formamide (18 µl) and then subjected to heat treatment (95 °C for 3 min, followed by rapid cooling to room temperature within a minute). Finally, the composition of each sample was analyzed by running 9 µl of each sample-formamide mixture in a lane of a 7.5 M urea-15% PAGE-1X TBE gel. Gel images were acquired using a Syngene G:Box Ef2 (Syngene; Cambridge, UK) gel imager, and band intensities were analyzed using our custom MATLAB code. Catalytic turnover at the designated time point was calculated by measuring the fraction cleaved (*FC*) using the formula:

$${FC}=({F}_{{cleaved\; product}}-{F}_{{background}})/({F}_{{cleaved\; product}}+{F}_{{substrate}}-2{F}_{{background}})$$

where *F*_*cleaved product*_ is the band intensity of the cleaved product, *F*_*substrate*_ is the band intensity of the full-length substrate, and *F*_*background*_ is the background intensity. This FC value is then multiplied by *C*_*substrate*_*/C*_*ribozyme*_, where *C*_*substrate*_ and *C*_*ribozyme*_ are the initial concentrations of the substrate and ribozyme, respectively. Temperature during the experiments was controlled by Eppendorf^TM^ Mastercycler^TM^ Nexus (Eppendorf corporate; Hamburg, Germany). Analysis of the cleavage rates by the 10-23 DNAzyme and the hairpin ribozyme were conducted in the same manner. These experiments were performed in triplicate if not mentioned separately. Uncropped gel images are provided in the Source data file.

### DNA nanostar motif and condensate preparation

To assemble DNA nanostars (NS), we prepared 20 µM oligonucleotides (NS1, NS2, and NS3) in a buffer containing 1.5 mM magnesium acetate and 20 mM Tris-HCl (pH 7.9). The annealing process consisted of three steps: thermal denaturation at 95 °C for 5 min, gradual cooling to 20 °C at a rate of − 0.5 °C/min, and incubation at 20 °C for 30 min. DNA nanostars were freshly prepared before each experiment. To induce LLPS, 5 µl of DNA nanostar solution (20 µM) was incubated with 5 µl of 4X RC buffer and 10 µl deionized water for 1 h at 37 °C.

To localize ribozymes, templates, or both within DNA droplets, we prepared two distinct DNA nanostars (NS-Bind and NS-template), combining NS1 with either NS1-Bind or NS1-Template nanostar species. For constructing ribozyme-localized droplets under 500 nM ribozyme conditions, we prepared a nanostar solution containing NS (4.5 µM), DNA nanostars with NS1-Bind sequence (NS-Bind; 0.5 µM), and HHR-3e (0.5 µM). This solution was incubated at 37 °C for 1 h to allow LLPS and compartmentalization of the ribozyme. All protocols for the preparation of nanostar mixtures for localizing specific components within DNA droplets followed two guidelines: (1) the sum of the concentrations of the different types of nanostars was set at 5 µM, and (2) the concentrations of DNA nanostars presenting complementary sequence for cargo (e.g., HHR-3e) were equal to those of ribozyme (HHR-3e) or non-template (nonTemplate). When we prepare NS-Bind that presents blunt ends, NS1-Bind, NS2-BE, and NS3-BE were employed. Likewise, NS-template with blunt ends was assembled by NS1-Template, NS2-BE and NS3-BE.

Imaging of DNA droplets was performed using a Zeiss LSM 780 and a FLUOVIEW™ FV4000 confocal laser scanning microscope. Zeiss LSM780 was equipped with 488 and 633 nm lasers with GFP and Cy5 filter sets. FV4000 was equipped with 488 and 640 nm lasers with a Volume Phase Hologram grating and slit. Image analysis was conducted using Zeiss ZEN 3.9 and ImageJ 1.54 f software. To calculate the partition coefficients of fluorophore-labeled strands, DNA droplet solutions were loaded into capillary tubes (Rectangle Glass Tubing 5010, VitroCom). Both ends of the tubes were then sealed with epoxy (Devcon 14250) on a plain glass slide (16004-424, VWR International). The prepared slides were subsequently analyzed using confocal microscopy at 37 °C. Z-stack images were acquired to identify the z-height corresponding to the center of each DNA droplet. Thereafter, fluorescence intensities inside (*F*_*inside*_) and outside (*F*_*outside*_) the droplets of interest were then measured. The partition coefficient was determined as the ratio of *F*_*inside*_ to *F*_*outside*_^54^. For all partition coefficient measurements, at least 10 different droplets were analyzed. The sizes of DNA droplets were analyzed using a custom MATLAB script that applied threshold-based segmentation to identify and measure the area and diameter of each condensate within a confocal fluorescence image. For fluorescence recovery after photobleaching (FRAP) experiments to investigate the mobility of DNA nanostars through DNA droplets, the fluorescence intensities in the images before and immediately after photobleaching were set as the values corresponding to 100% and 0% recovery, respectively, for normalization. After photobleaching, images were taken every 10 sec and then used to analyze fluorescence recovery time.

### LLPS-driven acceleration of RNA cleavage reactions

To evaluate the co-partitioning effect of the ribozymes and templates on cleavage turnover, we incubated NS (5.6 µM), NS-Bind (0.64 µM), NS-template (0.128 µM), HHR-3e (0.64 µM), nonTemplate (0.128 µM), and YIPP (0.00178 units/µl) in 1X RC buffer (15.6 µl) at 37 °C for 1 h. Following this incubation, 4 µl of Sca-16 at varying concentrations and 0.4 µl of T7 RNA polymerase (200 units/µl) were added to initiate transcription. In experiments localizing only templates, NS1-Bind-containing DNA nanostars, i.e., the combination consisting of NS1-Bind, NS2-BE, and NS3-BE, were used. Similarly, to partition ribozymes alone, we employed DNA nanostars that consisted of the strands NS1-Template, NS2-BE, and NS3-BE. After adding T7 RNA polymerase at 37 °C, we collected 2 µl of each experimental sample at each measured time point and immediately added 0.5 µl of c(HHR) (1.1 µM), a complementary strand designed to halt HHR cleavage reactions, to the sample. The collected samples were then heat-treated (90 °C for 5 min and cooled down to room temperature at a rate of − 0.1 °C/sec). Next, 0.5 µl of Ligator (Ligator Sub and Ligator Ribo, 25 µM) was added to each sample, and the mixture was incubated overnight at 37 °C. Samples were then mixed with > 99% formamide (17 µl) and subjected to heat treatment as mentioned above. Finally, 10 µl of each heat-treated sample was analyzed via gel electrophoresis as mentioned above. These experiments were performed in triplicate.

### Statistics and reproducibility

For replication, all experiments were performed using the same equipment, chemicals (see Methods), and experimental conditions across one to seven independent experimental days. Error bars in quantitative data represent ± one SD. Data in Fig. 4D, E were pooled from four independent experiments. Data in Fig. 4C were pooled from three independent experiments. No statistical method was used to predetermine sample size. No data were excluded from the analyses. The experiments in this paper were not randomized. All attempts at replication were successful.

### Reporting summary

Further information on research design is available in the Nature Portfolio Reporting Summary linked to this article.

## Supplementary information

Supplementary Information

Reporting Summary

Transparent Peer Review file

## Source data

Source Data

## Data Availability

All data presented in this manuscript are provided in the Supplementary Information and Source data file. Data are also available at: https://github.com/BH-Kang/Scavenger-project and https://doi.org/10.7281/T1ARUT0S^71^. Source data are provided in this paper.

## Code availability

Simulations for ribozyme kinetics, gel image analysis, optimizer function to fit a rate constant for substrate-driven product displacement, and droplet size measurement are available at: https://github.com/BH-Kang/Scavenger-project and https://doi.org/10.7281/T1ARUT0S^71^.
