# Supplementary material for: Reaction enhancement via product scavenging and phase separation-driven pathway canalization
Source: Nat Commun. 2026 Sep 28;17:11174. doi: 10.1038/s41467-026-77899-x (PMC13714313; doi:10.1038/s41467-026-77899-x)
Supplement: Supplementary file 1 [file 41467_2026_77899_MOESM1_ESM.pdf]

Supplementary Information

Reaction enhancement via product scavenging and phase separation-driven pathway canalization

(B. Kang *et al.*)

Table of Contents

1. **Supplementary notes, Tables, and Figures**

Supplementary Table 1: Oligonucleotide information

Supplementary Figure 1: Observed cleavage rates of HHR

Supplementary note 1: Reaction pathway of HHR-catalyzed substrate cleavage

Supplementary Figure 2: The RNA-cleaving mechanism of hammerhead ribozyme, and the rate of each step

Supplementary Figure 3: Turnover frequency of HHR with no scavenger

Supplementary Figure 4: Predicted catalytic turnover and turnover frequency of HHR with changed kinetic rates

Supplementary note 2: Design processes for scavengers

Supplementary Figure 5: Thermodynamic stabilities and secondary structures of complexes consisting of the Sca-16 and the HHR

Supplementary Table 2: Simulated complex formation between HHR and HHR Sub in the presence of Sca-16 and the 5' end product

Supplementary Figure 6: Cleavage reactions of HHR relying on seven different scavengers

Supplementary note 3: Modeling cleavage kinetics of HHR in the presence of scavenger

Supplementary Figure 7: Mechanism of HHR-mediated RNA cleavage in the presence of Sca-16

Supplementary Figure 8: Effects of hybridization and exchange rates between nucleic acids on HHR's turnover

Supplementary Figure 9: Full reaction-cycle diagram and kinetic parameters for the scavenger-assisted ribozyme reaction model.

Supplementary Figure 10: Turnover frequency of the HHR with Sca-16

Supplementary Figure 11: Turnover frequency of the HHR at varying [Sca-16]

Supplementary Table 3: Complex formation between a scrambled scavenger and a cleaved product

Supplementary Figure 12: Thermodynamic stability for hybridization between Sca-16-Scrambled4 and 3' end product

Supplementary note 4: Cleavage reactions mediated by various nucleic acid enzymes

Supplementary Figure 13: Substrate cleavage kinetics of 10-23 DNAzyme (10-23 D-HBV) and hairpin ribozyme (HairR).

Supplementary Table 4. Rate constants used in the model for all three nucleic acid enzymes

Supplementary Figure 14: Turnover frequency of 10-23 DNAzyme and Hairpin ribozyme with or without partner scavengers

Supplementary Figure 15: Turnover frequency of HHR when there is no substrate scavenging.

Supplementary Figure 16: Y-shaped DNA nanostar formation

Supplementary Figure 17: FRAP analysis of DNA nanostar droplet

Supplementary Figure 18: Effects of 3’ extension and nanostar binding to the extended 3’ end of hammerhead ribozyme on cleavage turnover

Supplementary Figure 19: Phase separation of ribozyme-labeled DNA nanostar

Supplementary Figure 20: Co-localization of hammerhead ribozymes and transcription templates in DNA droplets

Supplementary Table 5: Experimental conditions to check the multiphase effects on cleavage turnover

Supplementary Figure 21: Specific fluorescent labeling of ribozyme-catalyzed products

Supplementary Figure 22: Raw gel images corresponding to Fig. 5B-C.

Supplementary Figure 23: Transcriptional rates relying on the position of template and the presence of DNA droplets

Supplementary Figure 24: Demonstration of reaction pathway canalization using reporters

Supplementary Figure 25: Multiphase reaction system effect on cleavage reaction of ribozyme in the presence of excessive scavengers

Supplementary note 5: Quantification of HHR-mediated cleavage of HHR Sub when using ligation-based downstream reactions

Supplementary Figure 26: Quantitative analysis of multiphase-assisted cleavage reaction of HHR

2. Supplementary references

1. Supplementary notes, Tables, and Figures

Supplementary Table 1. Oligonucleotides used in this work.

Name	Sequence (5' to 3')	Modification	Length	Purification
HHR	ACGAGUACCGA<br>CUGAUGAGUCUGAAAAGACGAA<br>ACGAUCUGCA	-	43	Desalting
HHR Sub	UGCAGAUCGUCUCGGUACUCG<br>U CACAGGU	3' FAM	29	HPLC
Sca-16	ACCTGTGACGAGTACC	-	16	Desalting
NC-HHR Sub	UGCAGAUCGUGUCGGUACUCG<br>UCACAGGU	3' Atto488	29	HPLC
Sca-15	CCTGTGACGAGTA CC	-	15	Desalting
Sca-14	CTGTGACGAGTA CC	-	14	Desalting
Sca-17	GACCTGTGACGAGTACC	-	17	Desalting
Sca-18	AGACCTGTGACGAGTACC	-	18	Desalting
Sca-15-3'	ACCTGTGACGAGTAC	-	15	Desalting
Sca-14-3'	ACCTGTGACGAGTA	-	14	Desalting
Sca-16-Scrambled1	GGCATCACCTATAGGC	-	16	Desalting
Sca-16-Scrambled2	GCTCCCACAGTAGGAT	-	16	Desalting
Sca-16-Scrambled3	GCTCTGTACAAGACCG	-	16	Desalting
Sca-16-Scrambled4	ATTAAGTGACGCCCCG	-	16	Desalting
Sca-16-Scrambled5	GCGTGAGAATCCCCAT	-	16	Desalting
Sca-16-Scrambled6	AAATCGCCCGAGGTCT	-	16	Desalting
10-23 D-HBV	TGTGGTAGATTAGGCTAGCTACA<br>ACGAGAATTTGAGTAGTT	-	41	Desalting
10-23 D-HBV Sub	AUUACUUAACUACUCAAAUUCA<br>UAAUCUACCACA	3' FAM	34	HPLC
10-23 Sca-16	CGAGACGGGTCGTCCG	-	16	Desalting
HairR	CUCAGCUGCCACAGAGAAGUCA<br>ACCAGAGAAACACACGUUGUGG<br>UAUAUUACCUGGUACGCCGAAA<br>GGCGUGGUGGAC	-	78	Desalting
HairR Sub	GGUCCACCUGACAGUC<br>CUGUGGCAGCUGAG CAUGC	3' FAM	35	Desalting

HairR Sca-17	GCATGCTCAGCTGCCAC	-	17	Desalting
NS1	GCTAGCCAGTGAGGACGGAAGTTTGTCGTAGCATCGCACC	-	40	Desalting
NS2	GCTAGCCAACCACGCCTGTCCATTACTTCCGTCCTCACTG	-	40	Desalting
NS3	GCTAGCGGTGCGATGCTACGACTTTGGACAGGCGTGGTTG	-	40	Desalting
NS2-BE	CAACCACGCCTGTCCATTACTTCCGTCCTCACTG	-	34	Desalting
NS3-BE	GGTGCGATGCTACGACTTTGGACAGGCGTGGTTG	-	34	Desalting
NS1-Atto488	GCTAGCCAGTGAGGACGGAAGTTTGTCGTAGCATCGCACC	3’ Atto488	40	HPLC
NS1-Cy5	GCTAGCCAGTGAGGACGGAAGTTTGTCGTAGCATCGCACC	5’ Cyanine	40	HPLC
NS1-Bind	CGGGCAGGCAGGGCGTTTTTGCTAGCCAGTGAGGACGGAAGTTTGTCGTAGCATCGCACC	-	60	Desalting
HHR-3e	ACGAGUACCGACUGAUGAGUCUGAAAAGACGAAACGAUCUGCAUUCGCCCUGCCUGCCCG	-	60	Desalting
NS1-Template	CCTTTACCTGTGACGAGTACCGAGACGATCTGCACCTATAGTGAGTCGTATTAGAATTTTTGCTAGCCAGTGAGGACGGAAGTTTGTCGTAGCATCGCACC	-	101	Ultramer synthesis
nonTemplate	TTCTAATACGACTCACTATAGGTGCAGATCGTCTCGGTACTCGTCACAGGTAAAGG	-	56	Desalting
nonTemplate-FAM	TTCTAATACGACTCACTATAGGTGCAGATCGTCTCGGTACTCGTCACAGGTAAAGG	5’ FAM	56	HPLC
nonTemplate-TYE665	TTCTAATACGACTCACTATAGGTGCAGATCGTCTCGGTACTCGTCACAGGTAAAGG	5’ TYE665	56	HPLC
c(HHR)	TGCAGATCGTTTCGTCTTTTCAGACTCATCAGTCGGTACTCGT	-	43	Desalting
Sca-16-FAM	ACCTGTGACGAGTACC	3’ FAM	16	Desalting
Ligator Sub	GUCCUGUGGCAGCUGAGU	3’ FAM	18	Desalting
Ligator Ribo	CUCAGCUGCCACAGAGAAGCGAACCAGAGAAACACACGUUGUGGUAUAUUACCUGGUACGCCGAAAGGCGUUCUGCA	-	77	Desalting
HHR Sub-Rep-3Q	CCT TTA CCT GTG ACG AGT ACC GAG A	3’ Iowa Black® RQ	25	HPLC

HHR Sub-Rep-5F	TCT CGG TAC TCG TCA CAG GT	5’ Cy3	20	HPLC
Localized Rep-3Q	CGCCCTGCCTGCCCGTTTTTA CCTGTGACGAGTACCGAGACG ATCTGCACC	3’ Iowa Black® RQ	51	HPLC
Localized Rep-5F	GGTGCAGATCGTCTC	5’ TYE665	15	HPLC
Dispersed Rep-3Q	TTTTTA CCTGTGACGAGTACCGAGACG ATCTGCACC	3’ Iowa Black® FQ	36	HPLC
Dispersed Rep-5F	GGTGCAGATCGTCTC	5’ Atto488	15	HPLC

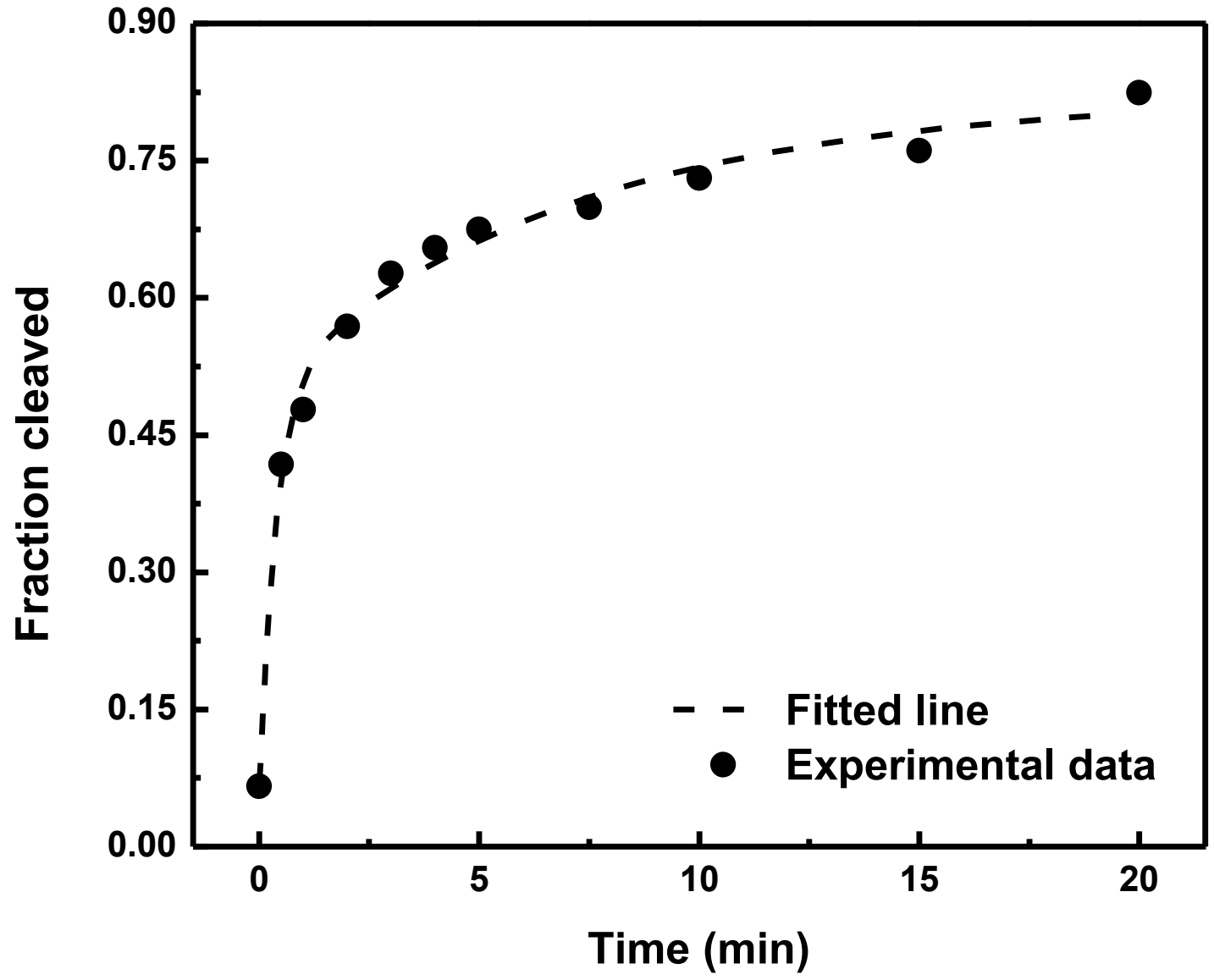

Supplementary Figure 1. The biphasic behavior of hammerhead ribozyme in cleaving its RNA substrate. 2.5 μM HHR and 0.5 μM HHR Sub were used in 1X RC buffer. We obtained the following regression curve, $F_c = F_0 - P_1(1 – \exp(-k_1t)) - P_2(1 – \exp(-k_2t))$ (F_0: 0.068, P1: 0.43, k_1: 2.54, P_2: 0.32, and k_2: 0.14) through least-squares fitting. The R-squared value of the regression curve is 0.99. The experiment was performed in duplicate.

Supplementary note 1. Reaction pathway of HHR-catalyzed substrate cleavage.

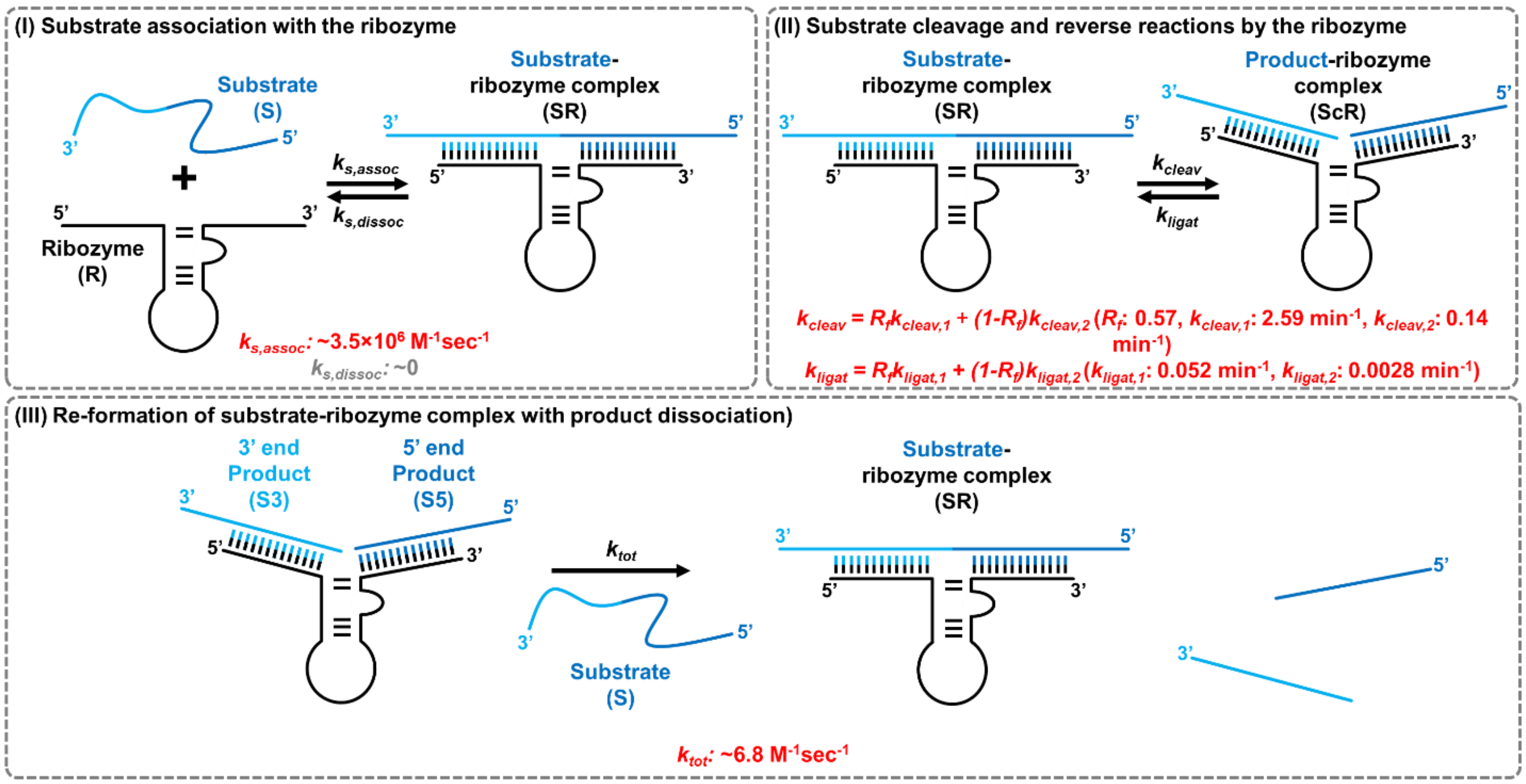

Supplementary Figure 2. The modeled RNA-cleaving mechanism of hammerhead ribozyme, and the rate of each step.

From the multistep reactions of hammerhead ribozyme in RNA cleavage (Supplementary Fig. 2), we obtained six equations to predict the catalytic turnover of HHR in a time-dependent fashion.

$$(Equation\ 1.1)\ \frac{d[S]}{dt} = -k_{s,assoc}[S][R] - k_{tot}[S][ScR]$$

$$(Equation\ 1.2)\ \frac{d[R]}{dt} = -k_{s,assoc}[S][R]$$

$$(Equation\ 1.3)\ \frac{d[SR]}{dt} = k_{s,assoc}[S][R] - R_f k_{cleav,1}[SR] + R_f k_{ligat,1}[ScR] -$$

$$(1 - R_f)k_{cleav,2}[SR] + (1 - R_f)k_{ligat,2}[ScR] + k_{tot}[S][ScR]$$

$$= k_{s,assoc}[S][R] - k_{cleav,avg}[SR] + k_{ligat,avg}[ScR] + k_{tot}[S][ScR]$$

$$(Equation\ 1.4)\ \frac{d[ScR]}{dt} = k_{cleav,avg}[SR] - k_{ligat,avg}[ScR] - k_{tot}[S][ScR]$$

$$(Equation\ 1.5)\ \frac{d[S3]}{dt} = k_{tot}[S][ScR]$$

$$(Equation\ 1.6)\ \frac{d[S5]}{dt} = k_{tot}[S][ScR]$$

Simulation was performed using Python's SciPy package (version 1.10.1) with the odeint library. Based on the two different cleavage rates of the hammerhead ribozyme in Supplementary Fig. 1, R_f and $1 - R_f$ were set to 0.57 and 0.43, respectively. In the present equations, we assumed that the fast and slow cleavage and ligation pathways act on the same substrate–ribozyme and product–ribozyme variables. This, the weighted averaged rates of cleavage and ligation rates ($k_{cleav,avg}$ and $k_{ligat,avg}$) were derived rather than an explicit representation of two separate ribozyme populations. From the above six equations, [S5] and [ScR] were obtained through numerical integration and then these two concentrations were summed to achieve the total concentration of 5' end product (5'P) over time. Catalytic turnover at a given time was calculated by $[5'P]/[R]_0$ at that time (here, $[R]_0$ is the initial concentration of the ribozyme). The fraction cleaved for a given time was calculated as $[5'P]/[S]_0$ for [5'P] at that time and $[S]_0$ as the initial concentration of substrate.

It should be noted that in this model, the release of the products by an incoming substrate was represented as a single effective second-order transition proportional to [S] and [ScR] (Supplementary Fig. 2, Step III). This is coarse-grained term rather than a mechanistically resolved elementary step and represents the overall exchange process by which product-bound ribozyme is converted back to the substrate-bound complex. Since the intermediate steps were not directly measured, they were lumped into one effective rate to provide a minimal phenomenological description of turnover.

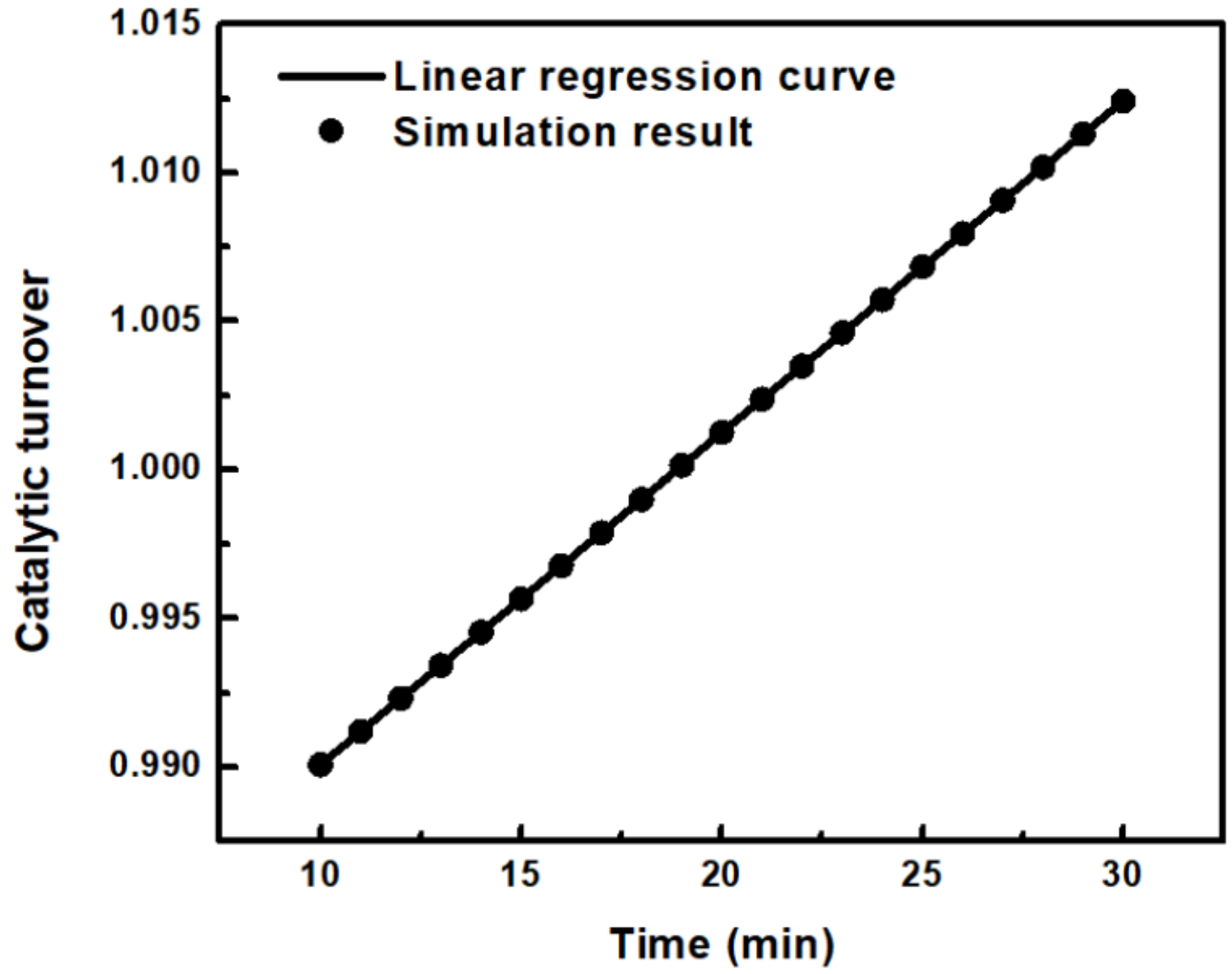

Supplementary Figure 3. Linear regression curve of simulated catalytic turnover of HHR over time in the absence of scavenger. Catalytic turnover of HHR was predicted using the kinetic model described in Supplementary note 1 and plotted from 10 to 30 min. Using this simulated data, we obtained the following regression curve, y = a + bx (a: 0.98, and b: 0.0011). The R-squared value of the regression curve is 1.0. From the slope of the graph (b value), the turnover frequency was calculated as 1.12×10^{-3} min^{-1} (0.067 h^{-1}).

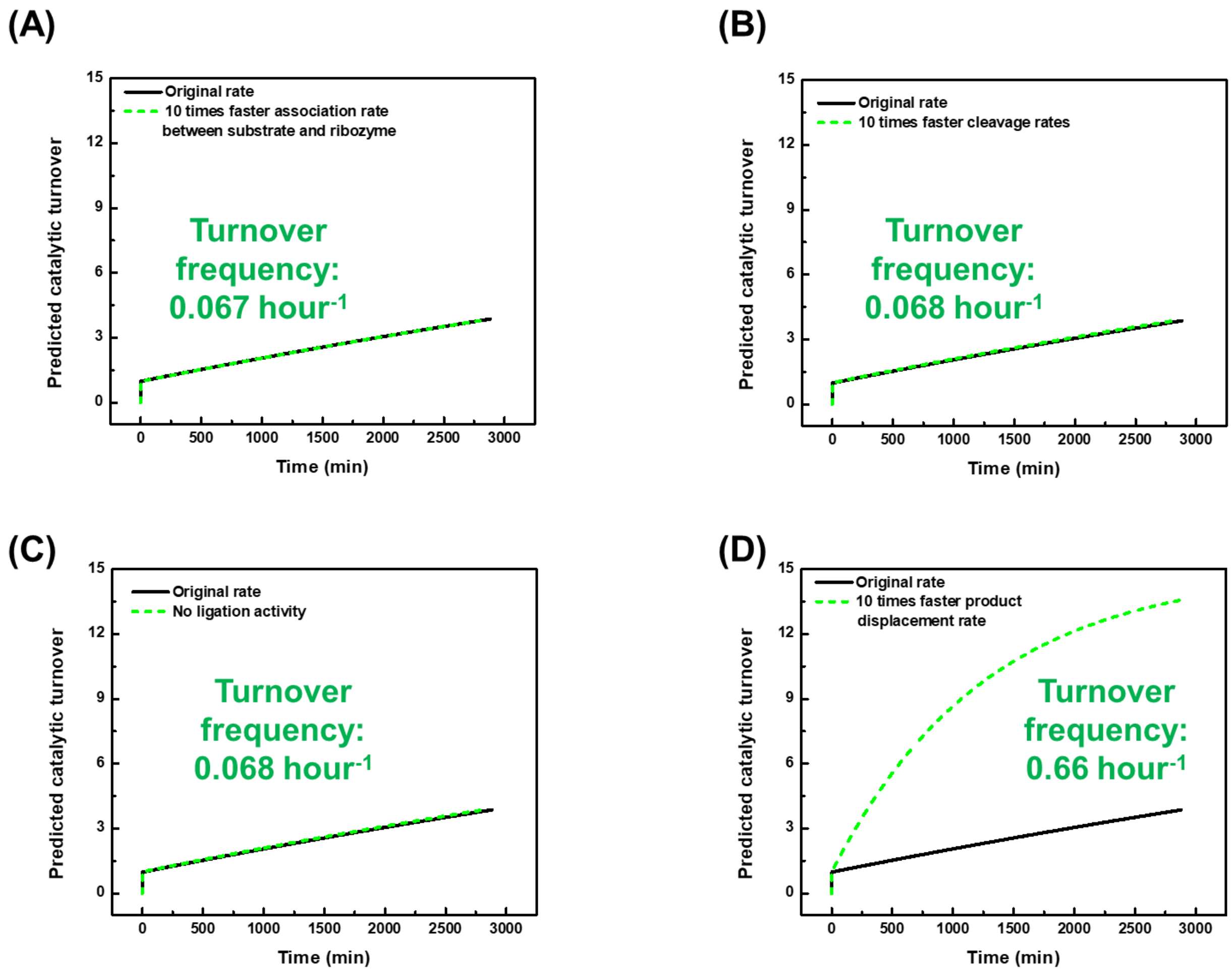

Supplementary Figure 4. Predicted catalytic turnover and turnover frequency of HHR over time according to the equations in Supplementary note 1 when four different kinetic rates in the cleavage mechanism were intentionally altered. (A) Effect of a 10-fold increase in the hybridization rate between the substrate and ribozyme ($k_{s,assoc} \approx 3.5\times10^{7}$ $M^{-1}s^{-1}$) on HHR-mediated catalysis. (B) Effect of a 10-fold increase in the cleavage rates of the HHR ($k_{cleav} \approx 25.4$ and $1.4\ min^{-1}$) on HHR-mediated catalysis. (C) Effect of no ligation activity ($k_{ligat} \approx 0$) on HHR-mediated catalysis. (D) Effect of a 10-fold increase in product displacement mediated by the full-length substrate on HHR-mediated catalysis. Only mitigating product inhibition (D) is effective in increasing the turnover frequency of HHR.

Supplementary note 2. Design processes for scavengers that perform product scavenging.

To design scavengers that selectively sequester one of the two products (3’P) of ribozyme-mediated cleavage reactions, we performed a three-step analysis. First, using NUPACK in both DNA-only and RNA-only modes,[1] we evaluated the thermodynamic stability of each scavenger candidate in complex with its target product (3’P) and with the full-length substrate (HHR Sub) (Supplementary Fig. 5, top and middle panels). We also assessed the thermodynamic stability of complexes formed between the ribozyme (HHR) and either its substrate or 3’P (Supplementary Fig. 5, bottom panel). This analysis was used to identify scavenger candidates that bind 3′P more strongly than 3′P binds to HHR, while avoiding excessive stabilization of scavenger–substrate complexes.

Second, we estimated whether each scavenger candidate could provide an accelerated kinetic pathway for product removal via toehold-mediated strand exchange. Specifically, scavengers were designed to displace HHR-bound 3′P much faster than spontaneous substrate-mediated replacement of the old product, based on expected rates of toehold-mediated strand-exchange reactions.[2-5] For example, the apparent rate constant for formation of a new substrate–ribozyme complex through substrate-mediated displacement of the old product was 6.8 $M^{-1}s^{-1}$ (Supplementary Fig. 2). Therefore, the scavenger was designed to initiate strand exchange reaction through a 3′ toehold of 3’P longer than 4 nt, which is expected to promote substantially faster product displacement than the spontaneous substrate-mediated pathway.

Next, we tested whether 3’P was preferentially released from HHR Sub to form the scavenger:3’P complex in simulations containing HHR Sub, 5’P, 3’P, and the scavenger candidate at 1 μM each. Under these conditions, the predicted formation of the Sca-16:3’P complex was 99.7 and 98.2% in the RNA- and DNA-only simulations, respectively. Finally, assuming complete sequestration of 3’P by the scavenger, we examined whether the HHR:HHR Sub complex could still form efficiently at equilibrium in the presence of 5’P, HHR, HHR Sub and scavenger (Supplementary Table 2). To model conditions in which the scavenger was present in excess over the ribozyme, NUPACK simulations were performed with scavenger concentrations of 1X, 10X, and 100X relative to the other species. In this study, we selected the scavenger sequence that maintained >50% formation of the HHR:HHR Sub complex at equilibrium in both DNA-only and RNA-only NUPACK simulations.

We also note that scavenger design may affect not only the initial displacement of HHR-bound 3′P, but also the subsequent toehold-exchange reaction between the ribozyme-bound product complex and a scavenged substrate (Supplementary Fig. 6). Variants with longer residual output toeholds modestly improved turnover compared with matched shorter-toehold designs, consistent with a beneficial role for this toehold.

However, the limited magnitude of this effect, together with the similar activity of variants containing 0–2 nt output toeholds, suggests that scavenger performance reflects multiple coupled design constraints rather than output-toehold length alone.

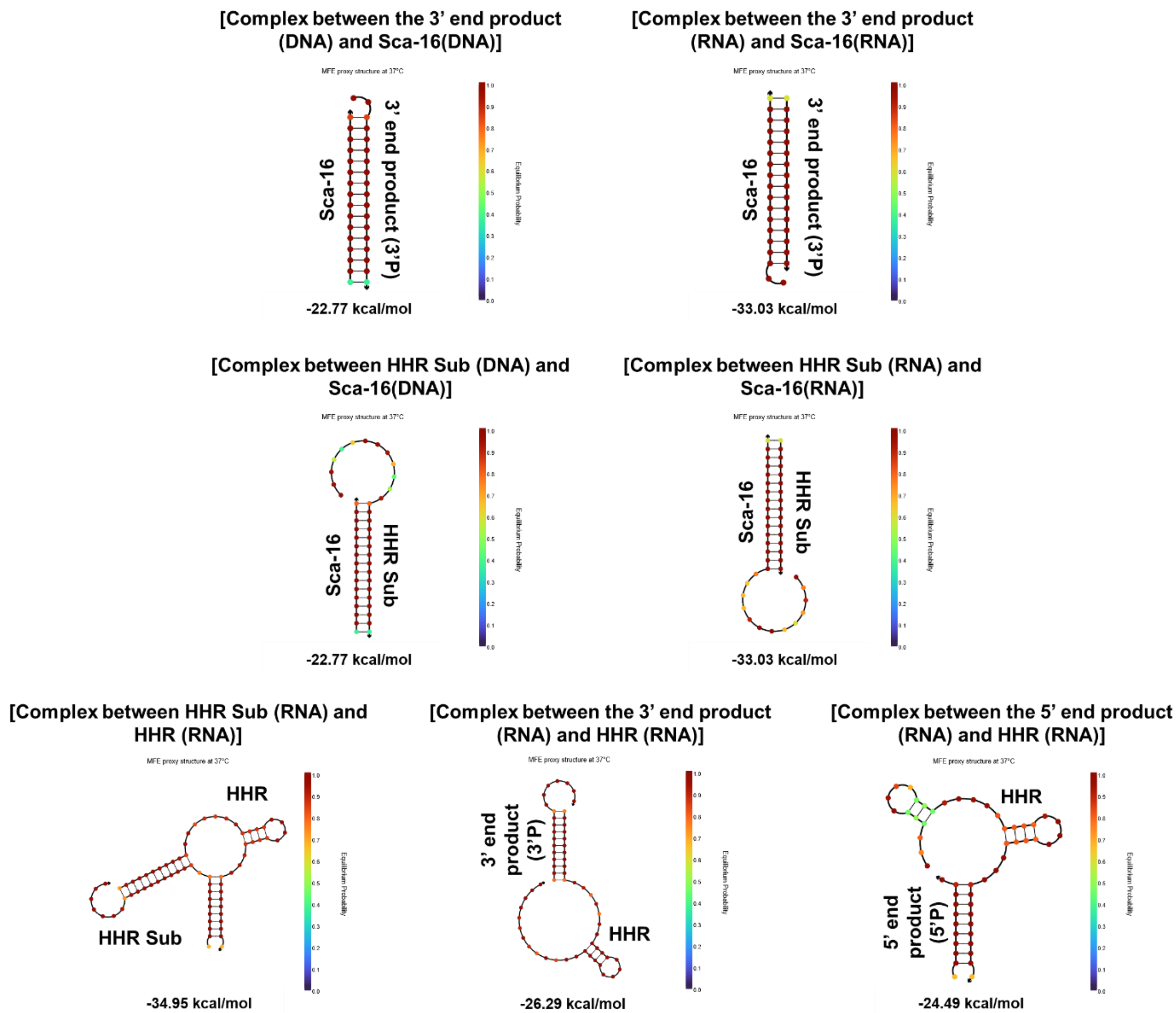

Supplementary Figure 5. Thermodynamic stabilities and secondary structures of nucleic acid complexes containing Sca-16 or HHR, predicted by NUPACK simulations[1]. Because NUPACK does not support direct thermodynamic predictions for DNA:RNA hybrids, we estimated the thermodynamic stabilities of Sca-16:3'P and Sca-16:HHR Sub complexes using DNA-only and RNA-only versions of oligonucleotides as simplified proxy systems (top and middle panels). These calculations should therefore be interpreted qualitatively. Because the relative stability of DNA:RNA and DNA:DNA duplexes is dependent on relative purine content,[6,7] given 50% purine contents in the 3'P and Sca-16, we inferred that Gibbs free energy of the Sca-16 (DNA): 3'P (RNA) complex falls between -22.77 and -33.03 kcal/mol. Consequently, this complex is predicted to be less stable than the HHR:HHR Sub complex (first structure in the bottom panel) but more stable than the HHR:3'P complex (second structure in the bottom panel). Based on these predictions, Sca-16 is expected to effectively displace the 3'P from the HHR but is unlikely to detach the full-length substrate. Simulation conditions: 1 μM oligonucleotides, 37 °C temperature, rna06 or dna04 parameters, All stacking ensemble, and 1 M Na^+ salts.

Supplementary Table 2. NUPACK-predicted equilibrium fraction of HHR substrate bound to HHR in the presence of Sca-16. The percentage was calculated as [HHR:HHR Sub]/[HHR Sub]$_{total}$×100, i.e., the predicted fraction of HHR Sub bound to HHR at equilibrium. Predictions were calculated for two conditions: without the 5’ cleavage product (5’P) and with the 5’P present. When performing simulations, all analyzed oligonucleotides were present at once.

Supplementary Table 2A. Without 5’ cleavage product

Predicted percentages of HHR Sub bound to HHR in the presence of Sca-16

Ratio of Sca-16 to other oligos[a]	Nucleic acid type	
	RNA[b]	DNA[c]
1X	99.8%	98.6%
10X	99.2%	95.2%
100X	99.0%	86.8%

Supplementary Table 2B. With 5’ cleavage product

Predicted percentages of HHR Sub bound to HHR in the presence of Sca-16 and 5’P

Ratio of Sca-16 to other oligos[d]	Nucleic acid type	
	RNA	DNA
1X	78.1%	61.9%
10X	72.8%	56.4%
100X	72.7%	55.0%

[a]Sca-16, HHR Sub, and HHR were used. Here, as HHR, sequence including the two binding arms and shortened catalytic core was used. [Sca-16] was fixed at 1 μM, and the concentrations of the other oligonucleotides were varied according to the indicated ratio (for example, at 10X, 1 μM Sca-16, 100 nM HHR Sub, and 100 nM HHR were used). [b]We used the RNA version of Sca-16. [c]We used the DNA versions of HHR, HHR Sub, and the 5’P. Simulation conditions: 37 °C temperature, rna06 or dna04 parameters, All stacking ensemble, and 1 M Na^+ salts. [d]Sca-16, HHR Sub, HHR, and 5’P were used.

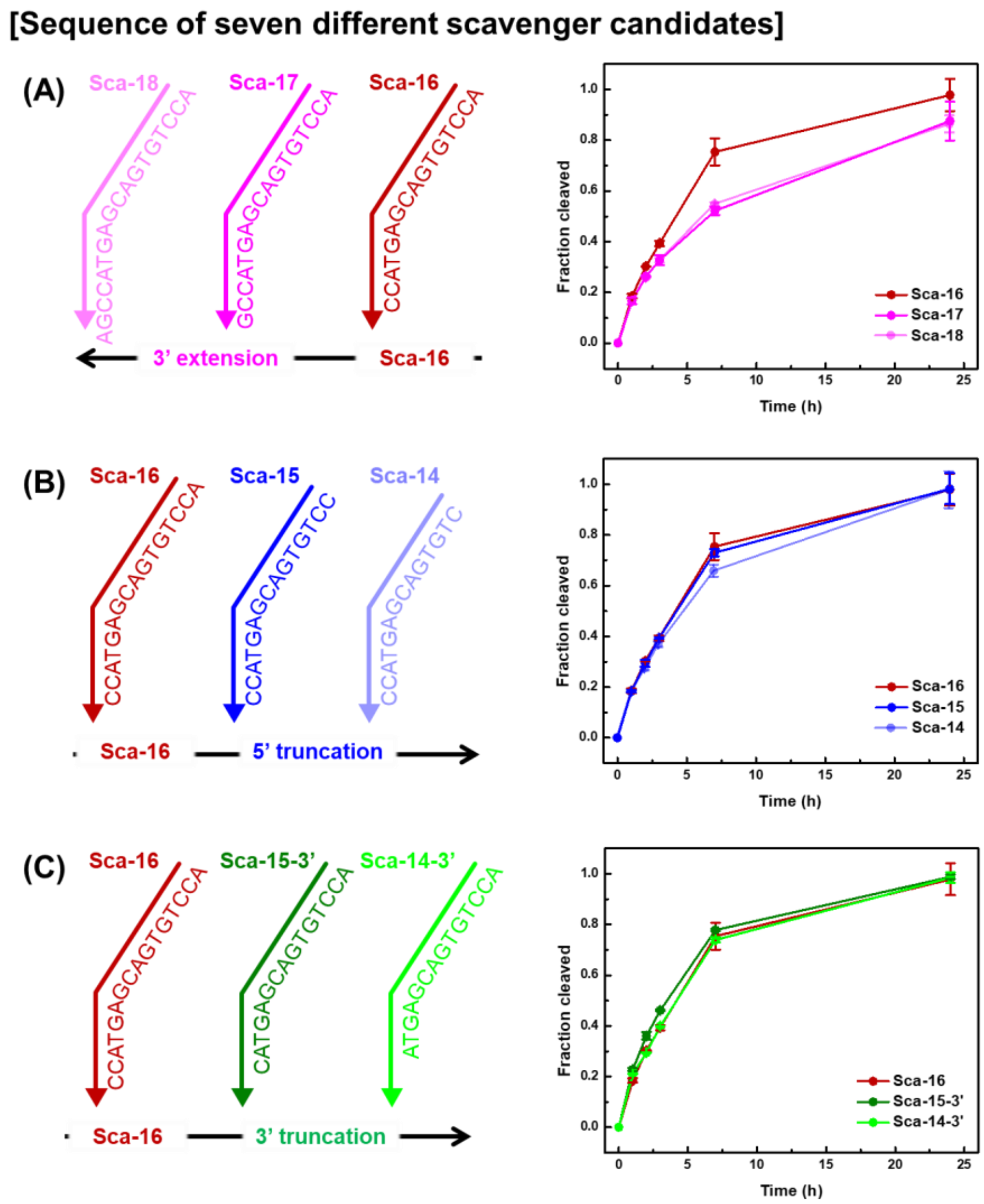

Supplementary Figure 6. Investigation of the effects of seven different scavengers with different lengths on RNA cleavage reactions. Sca-17 and Sca-18 were designed by extending the 3' end of Sca-16 (A). Sca-15 and Sca-14 were 5' truncated versions of Sca-16 (B), while Sca-15-3' and Sca-14-3' were 3'-truncated versions (C). Compared to no scavenger condition (200 nM HHR and 3 µM HHR Sub), all seven scavengers (20 µM) successfully accelerated cleavage turnover, achieving much higher fractions cleaved than without scavenger. Among scavengers of equal length, Sca-15-3' and Sca-14-3' outperformed Sca-15 and Sca-14, respectively, possibly because the scavenged substrate forms longer toeholds when reacting with the ribozyme. These experiments were performed in duplicate.

Supplementary note 3. Modeling cleavage kinetics of HHR in the presence of scavengers.

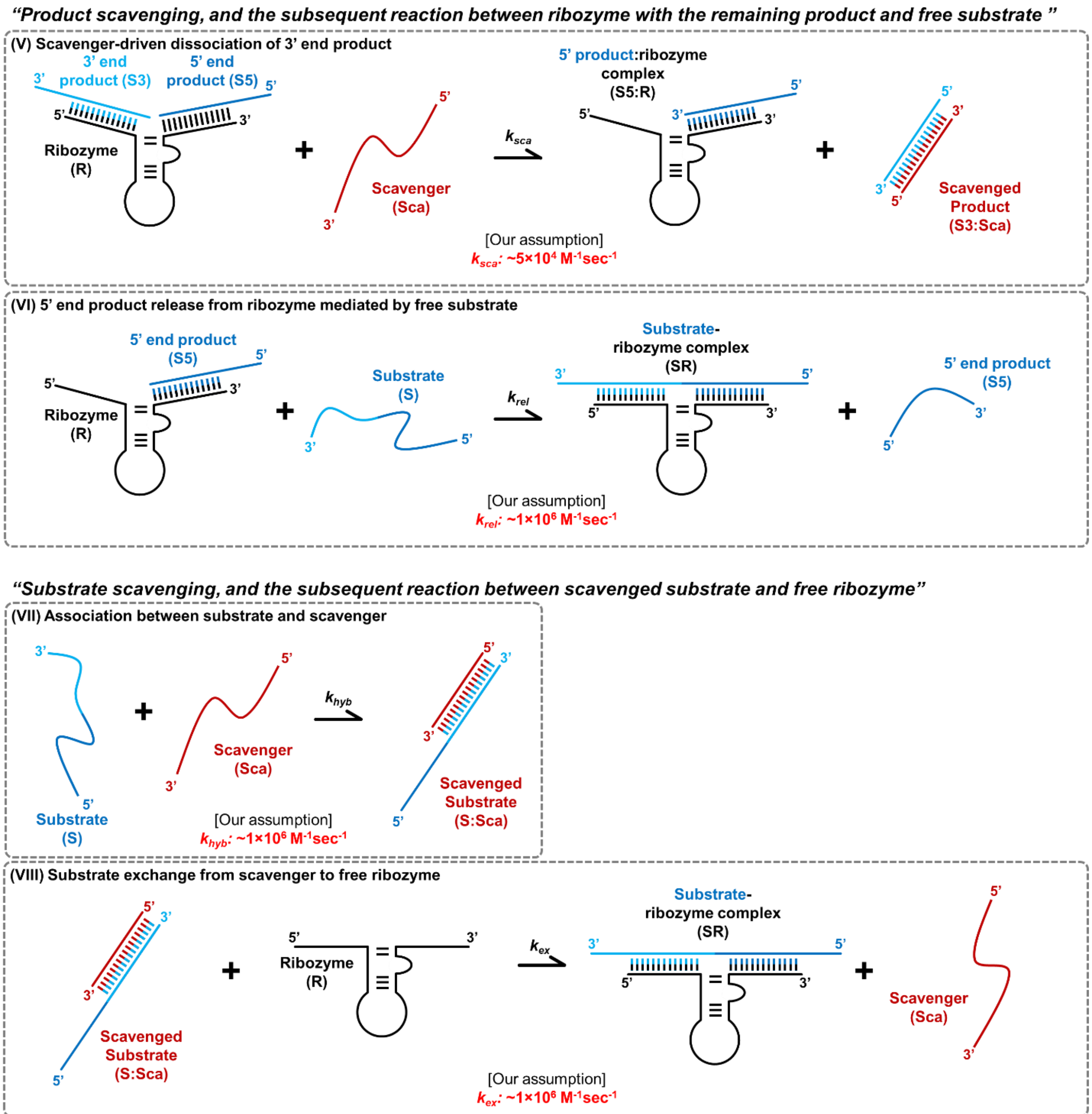

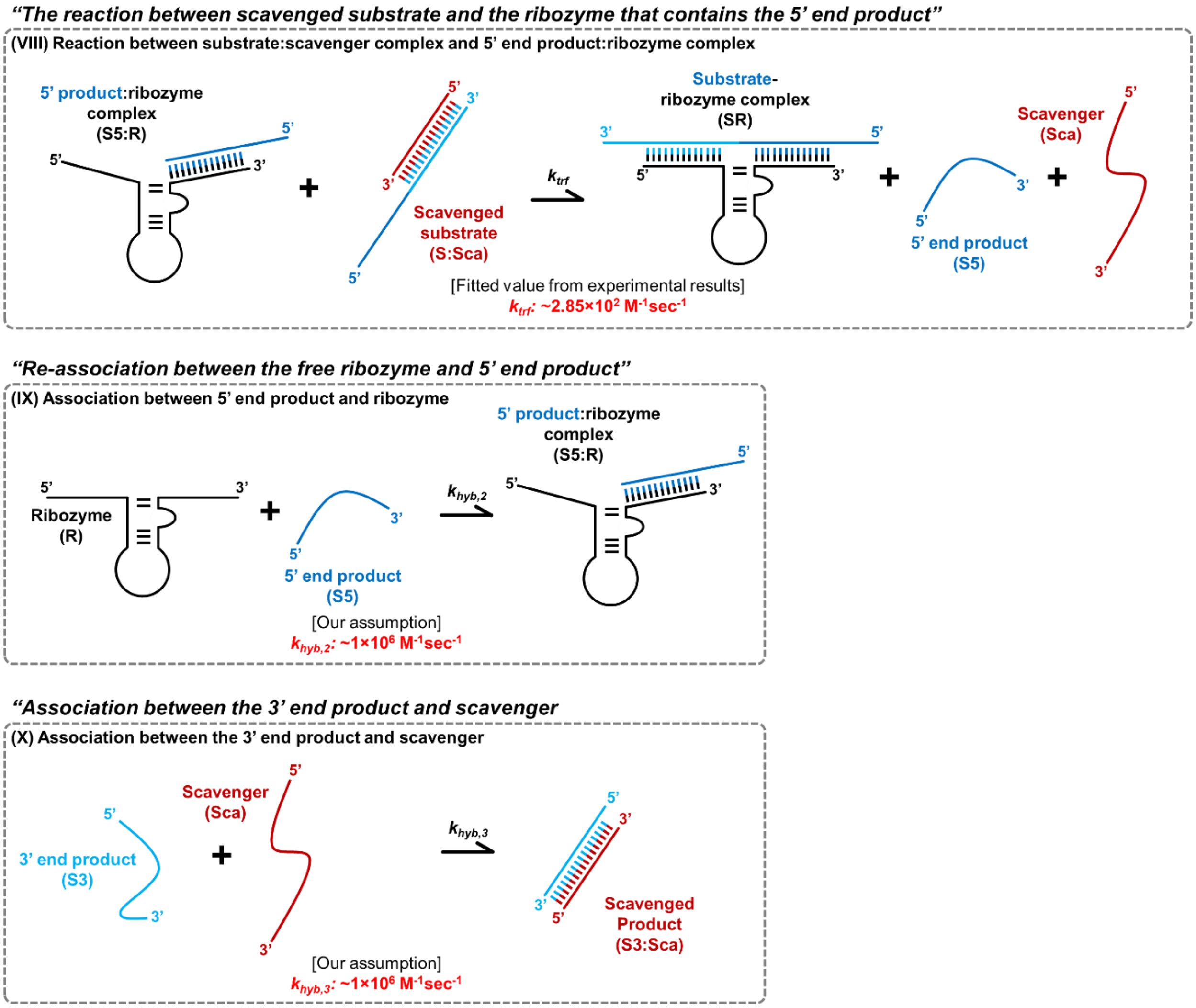

Supplementary Figure 7. The expected mechanism of HHR-mediated RNA cleavage in the presence of scavenger.

In the reaction mechanism of hammerhead ribozyme-driven RNA cleavage (Supplementary Fig. 2) which includes (1) association between ribozyme and substrate, (2) cleavage and ligation of substrate, and (3) substrate-assisted displacement of products to re-form the substrate-ribozyme complex, expected reactions involving scavengers (Supplementary Fig. 7) were considered. We obtained ten equations to predict catalytic turnover of ribozyme in the presence of scavenger.

$$(Equation\ 2.1)\ \frac{d[S]}{dt} = -k_{s,assoc}[S][R] - k_{hyb}[S][Sca] - k_{rel}[S][S5{:}R] - k_{tot}[S][ScR]$$

$$(Equation\ 2.2)\ \frac{d[R]}{dt} = -k_{s,assoc}[S][R] - k_{ex}[S:Sca][R] - k_{hyb,2}[S5][R]$$

$$(Equation\ 2.3)\ \frac{d[SR]}{dt} = k_{s,assoc}[S][R] - R_f k_{cleav,1}[SR] + R_f k_{ligat,1}[ScR] -$$

$$(1 - R_f)k_{cleav,2}[SR] + (1 - R_f)k_{ligat,2}[ScR] + k_{ex}[S:Sca][R]$$

$$+k_{rel}[S][S5:R] + k_{trf}[S5:R][S:Sca]$$

$$(Equation\ 2.4)\ \frac{d[ScR]}{dt} = R_f k_{cleav,1}[SR] - R_f k_{ligat,1}[ScR] + (1 - R_f)k_{cleav,2}[SR] -$$

$$(1 - R_f)k_{ligat,2}[ScR] - k_{sca}[ScR][Sca]$$

$$(Equation\ 2.5)\ \frac{d[S5:R]}{dt} = k_{sca}[ScR][Sca] + k_{hyb,2}[S5][R] -$$

$$k_{rel}[S][S5:R] - k_{trf}[S5:R][S:Sca]$$

$$(Equation\ 2.6)\ \frac{d[S5]}{dt} = -k_{hyb,2}[S5][R] + k_{rel}[S][S5:R] +$$

$$k_{trf}[S5:R][S:Sca] + k_{tot}[S][ScR]$$

$$(Equation\ 2.7)\ \frac{d[Sca]}{dt} = -k_{hyb}[S][Sca] + k_{ex}[S:Sca][R] -$$

$$k_{sca}[ScR][Sca] + k_{trf}[S5:R][S:Sca] - k_{hyb,3}[S3][Sca]$$

$$(Equation\ 2.8)\ \frac{d[S:Sca]}{dt} = k_{hyb}[S][Sca] - k_{ex}[S:Sca][R] - k_{trf}[S5:R][S:Sca]$$

$$(Equation\ 2.9)\ \frac{d[S3:Sca]}{dt} = k_{sca}[ScR][Sca] + k_{hyb,3}[S3][Sca]$$

$$(Equation\ 2.10)\ \frac{d[S3]}{dt} = k_{tot}[S][ScR] - k_{hyb,3}[S3][Sca]$$

From these equations, [S5], [S5:R], and [ScR] were acquired and then summed to achieve the final 5' end product (5'P) concentration in a time-dependent manner. Catalytic turnover was calculated by $[5'P]/[R]_0$. Fraction cleaved was calculated by $[5'P]/[S]_0$ (here, $[S]_0$ means the initial concentration of substrate). Here, we did not perform a separate direct binding assay between nucleic acid strands. Instead, the relative tendency for their interactions was evaluated using standard nucleic-acid thermodynamic/design analysis based on sequence complementarity, together with concentration-dependent kinetics of

the full reaction system; the kinetic parameters used were taken from previous studies or constrained from experimental data.

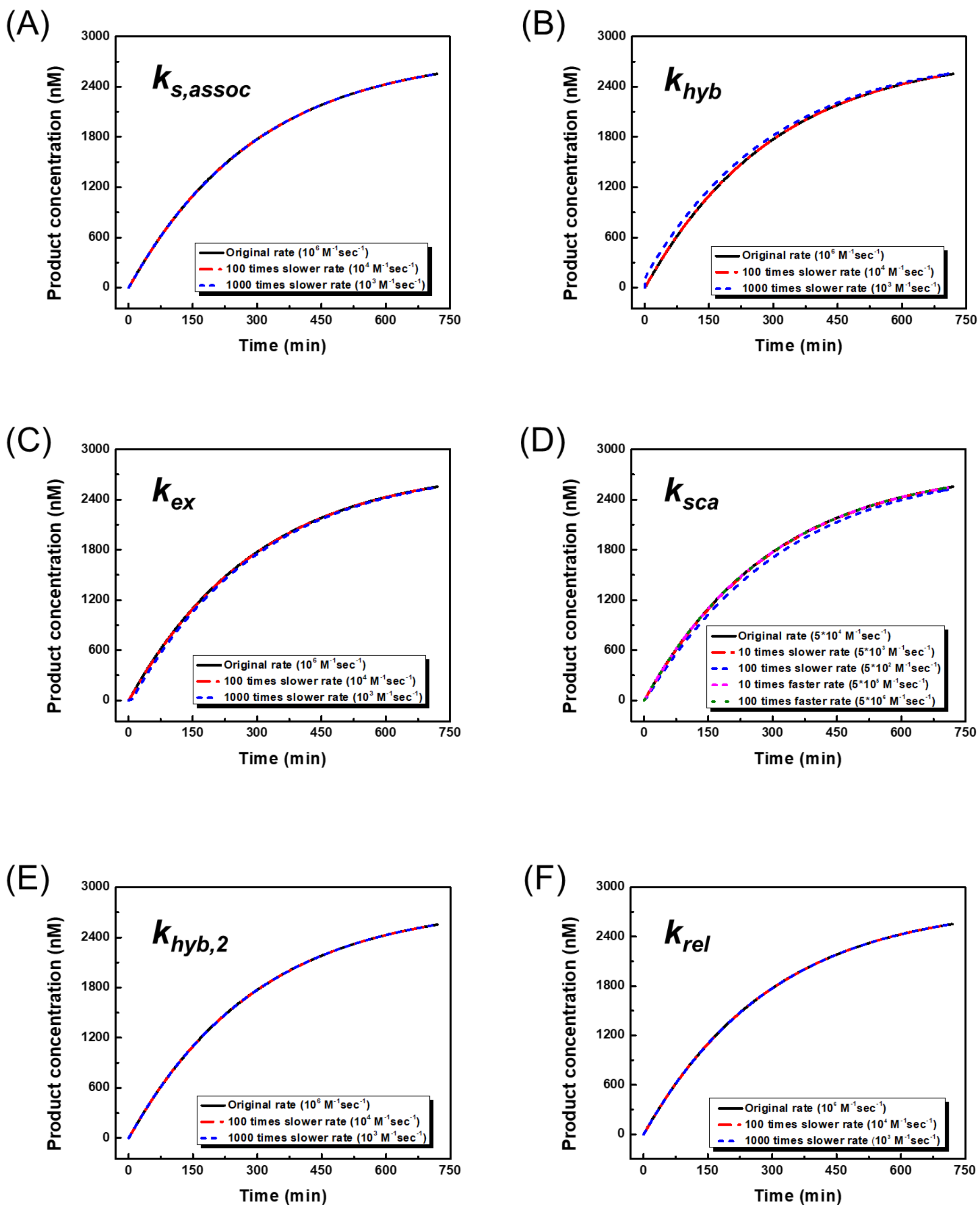

Supplementary Figure 8. Negligible effects of six rate constants on HHR's product formation. Even 2- or 3-orders-of-magnitude decrease in either of these rates does not affect the HHR's cleaved product (5'P) formation over time. Moreover, because the rate of substrate transfer from the scavenger to the ribozyme that possesses the 5'P is rate-limiting step, even 100 times faster product scavenging (D) does not enhance HHR's turnover.

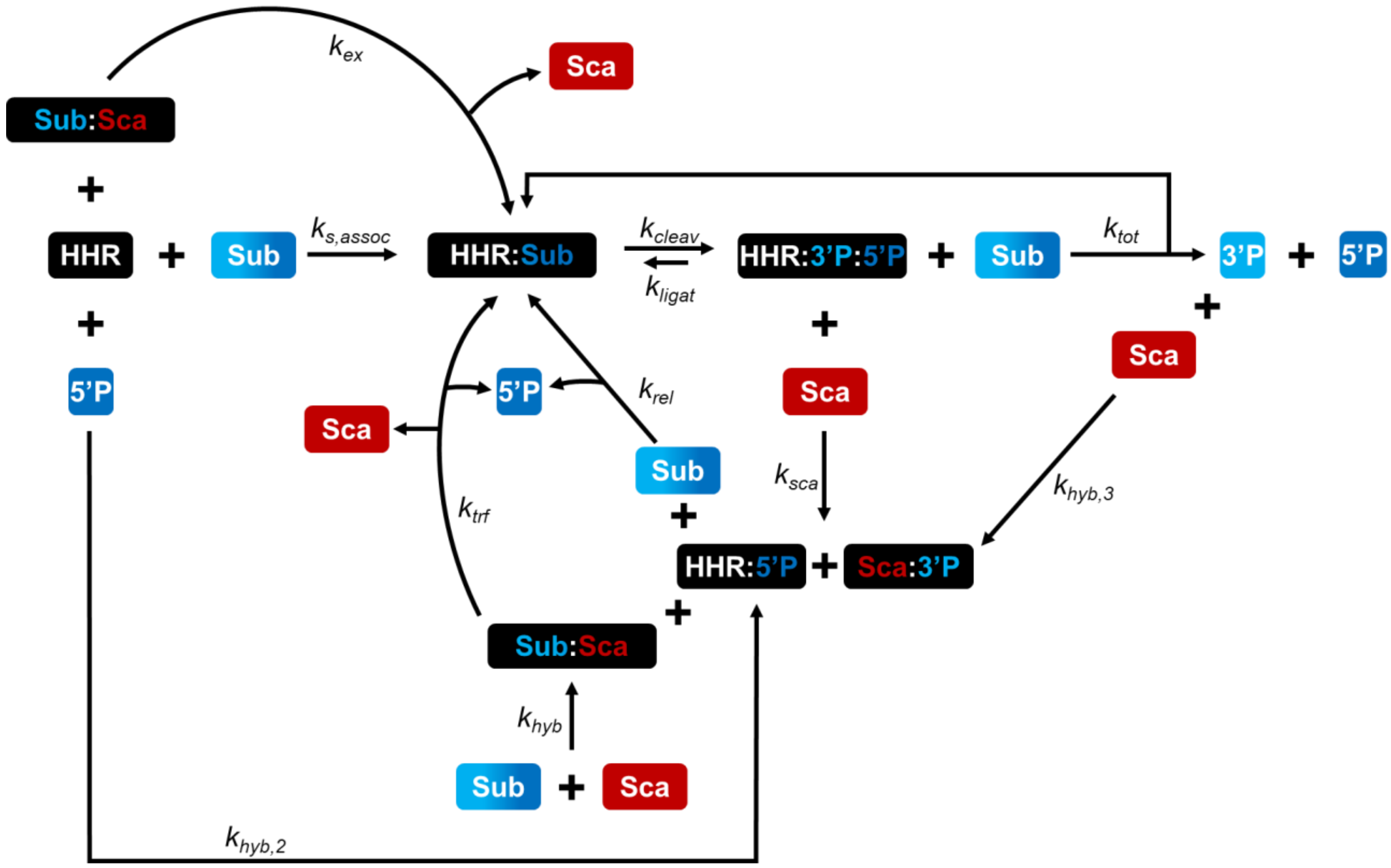

Supplementary Figure 9. Full reaction-cycle diagram and kinetic parameters for the scavenger-assisted ribozyme reaction model incorporated into the kinetic model in Supplementary notes 1 and 3. All modeled reaction pathways and associated kinetic parameters are shown.

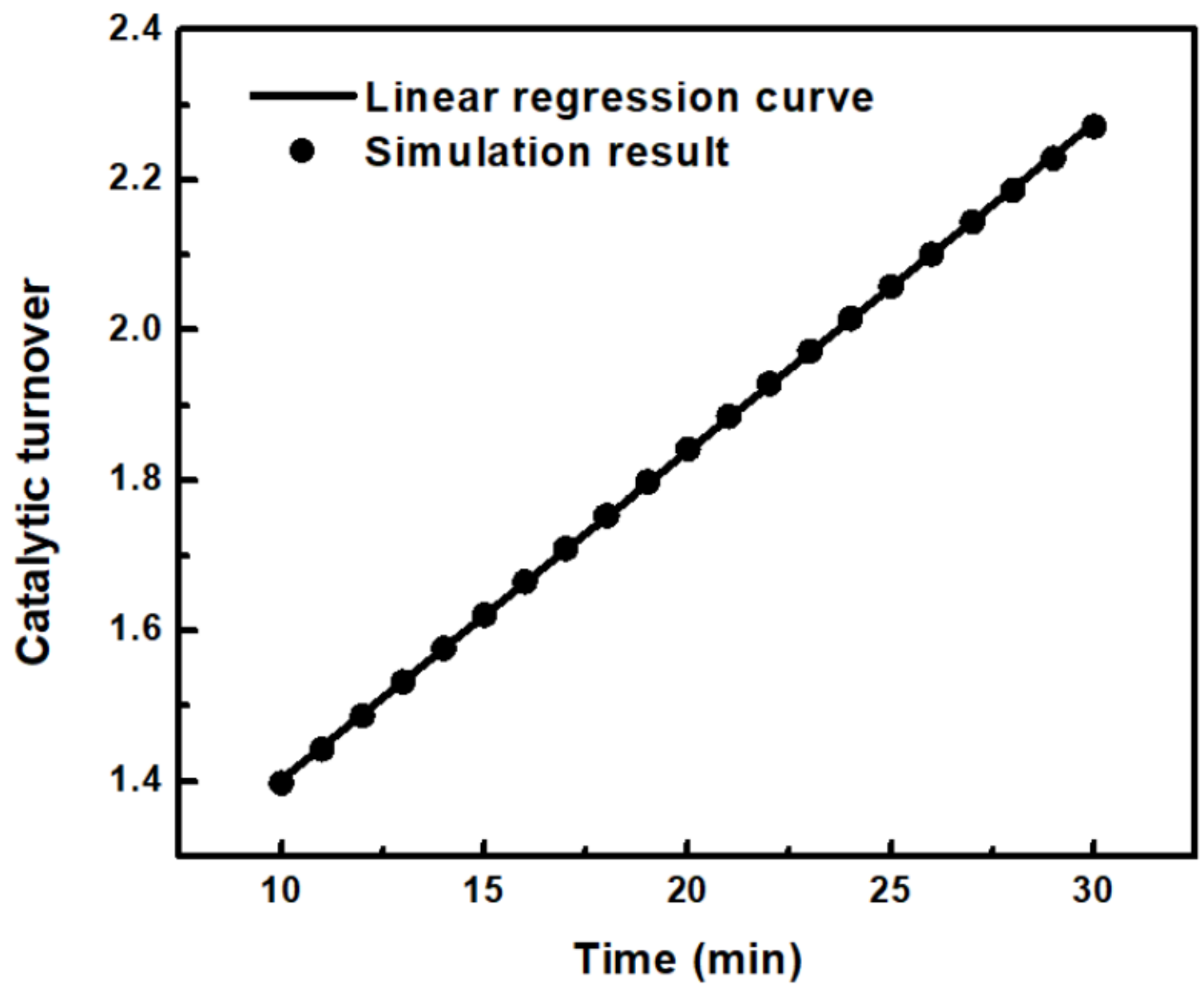

Supplementary Figure 10. Linear regression of simulated catalytic turnover of HHR over time in the presence of Sca-16. Catalytic turnover of HHR was predicted using the kinetic model described in Supplementary note 3 and plotted from 10 to 30 min. We obtained the following regression curve, y = a + bx (a: 0.97, and b: 0.044). The R-squared value of the regression curve is >0.99. From the slope of the graph (b value), turnover frequency was calculated as 4.4×10^{-2} min^{-1} (2.62 h^{-1}).

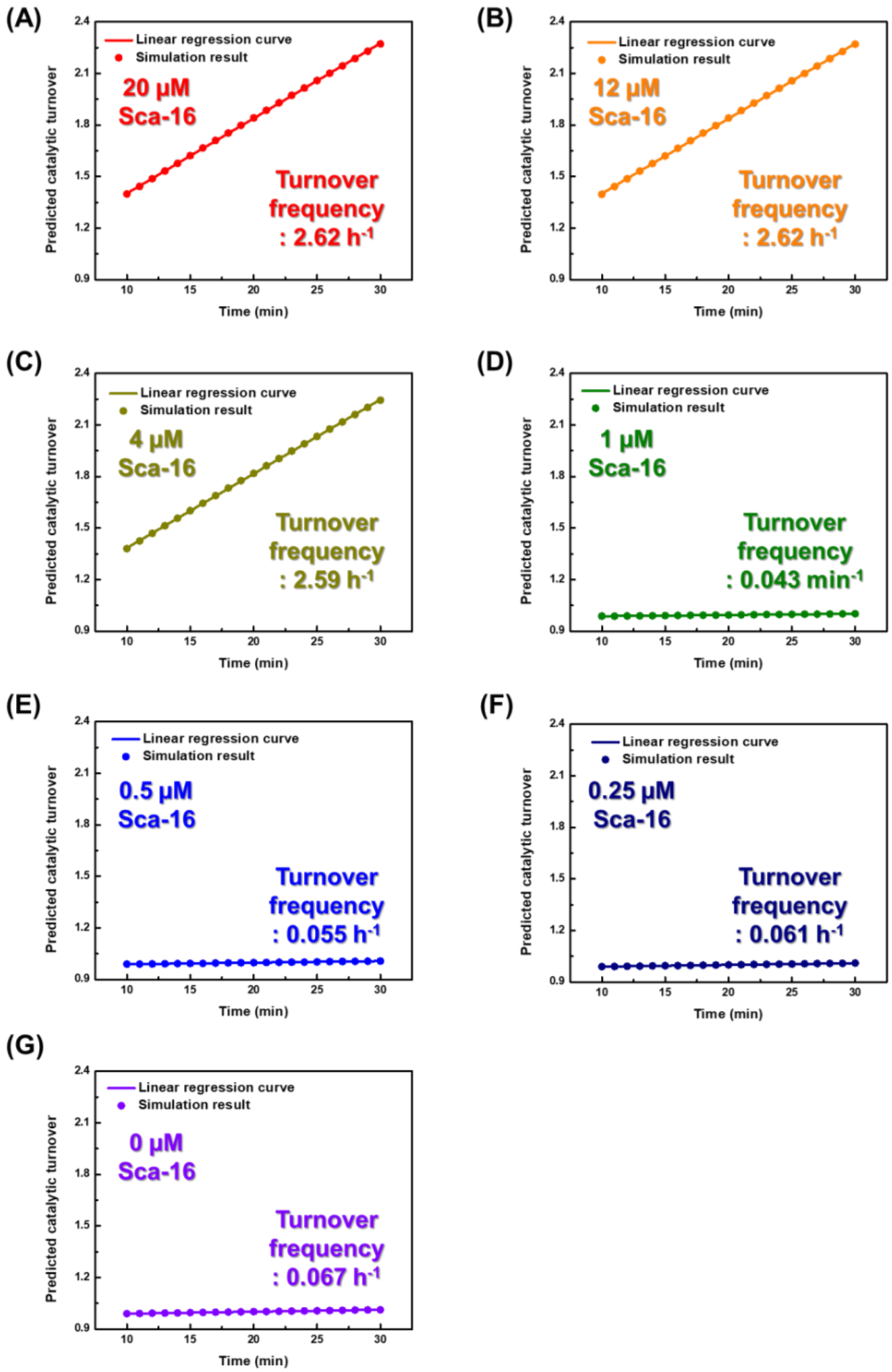

Supplementary Figure 11. Turnover frequency of HHR at varying concentrations of Sca-16. In this simulation, the concentrations of HHR and HHR Sub were fixed at 200 nM HHR and 3 µM, respectively. When the concentration of Sca-16 is higher than that of HHR Sub, scavengers successfully enhanced turnover frequency of HHR (A to C).

However, when the concentration of Sca-16 is lower than that of HHR Sub, its presence decelerated cleavage turnover of HHR (D to G).

Supplementary Table 3. NUPACK simulations[a] to predict the concentrations of complexes consisting of one of two products (3'P or 5'P) and scrambled scavengers.

	Complex formation with 3'P (nM)	Complex formation with 5'P (nM)
Sca-16-Scrambled1	0.290	0.517
Sca-16-Scrambled2	0.125	0.143
Sca-16-Scrambled3	1.84	0.391
Sca-16-Scrambled4	130	1.83
Sca-16-Scrambled5	1.25	0.214
Sca-16-Scrambled6	1.25	0.545

[a]Simulation conditions were set to complexes between scavenger and DNA versions of 3'P and 5'P, 37 °C temperature, dna04 parameters, All stacking ensemble, 1 M Na^+ salts, and 1 μM strand each.

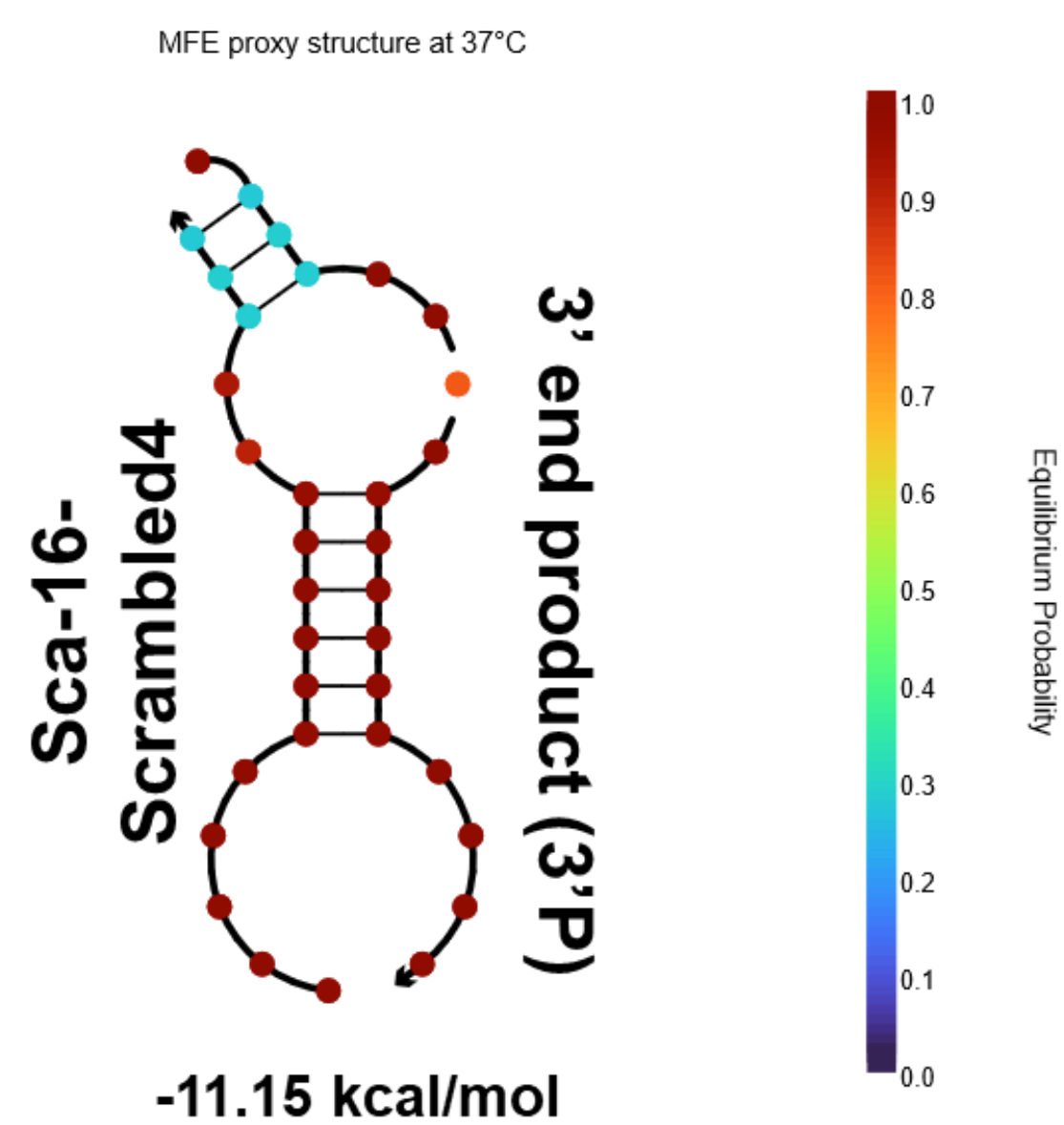

Supplementary Figure 12. NUPACK simulation to predict complex formation between Sca-16-Scrambled4 and the 3'P. Although Sca-16-Scrambled4 could hybridize with the 3'P, its thermodynamic stability is much lower than the stability of the complex consisting of the 3'P and HHR (Supplementary Fig. 5). Thus, the Sca-16-Scrambled4 is unable to displace the product from HHR. The NUPACK simulation conditions are the same as in Supplementary Table S3.

Supplementary note 4. Cleavage reactions mediated by various nucleic acid enzymes

To understand how scavengers accelerate reactions involving various RNA-cleaving nucleic acid enzymes (10-23 DNAzyme and hairpin ribozyme), we used the model described in Supplementary note 3 but changed relevant rate constants to correspond to those for of these ribozymes and their cleavage reactions (Supplementary Fig. 13 and Supplementary Table 4).

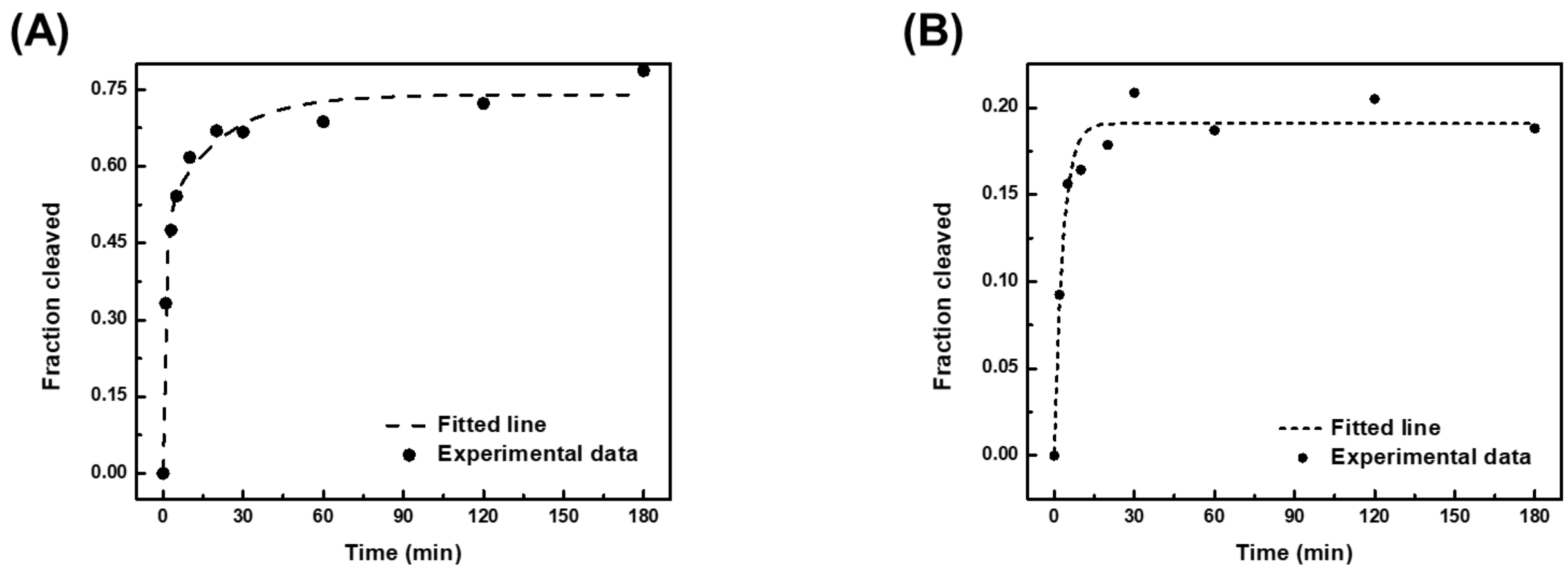

Supplementary Figure 13. Substrate cleavage kinetics of a 10-23 DNAzyme (10-23 D-HBV) and a hairpin ribozyme (HairR). (A) 2.5 μM 10-23 D-HBV and 0.5 μM 10-23 D-HBV Sub were used, and we obtained the following regression curve, $F_c = F_0 - P_1(1 – \exp(-k_1t)) - P_2(1 – \exp(-k_2t))$ (F_0: 0.0032, P1: 0.50, k_1: 0.96, P_2: 0.24, and k_2: 0.048). The R-squared value of the regression curve is 0.976. (B) 30 μM HairR and 3 μM HairR Sub were used, and we obtained the following regression curve, $F_c = F_0 - P_1(1 – \exp(-k_1t))$ (F_0: 0.0018, P1: 0.19, k_1: 0.31). The R-squared value of the regression curve is 0.961. From these observations, we acquired rate constants for substrate cleavage catalyzed by 10-23 D-HBV and HairR. Each experiment was performed in duplicate.

Supplementary Table 4. Rate constants used in the kinetic models for all three nucleic acid enzymes.

	Hammerhead ribozyme	10-23 DNAzyme	Hairpin ribozyme
$k_{s,assoc}$	$3.5×10^6$ $M^{-1}s^{-1}$	$3.5×10^6$ $M^{-1}s^{-1}$	$3.5×10^6$ $M^{-1}s^{-1}$
$k_{s,dissoc}$	0	0	0
R_f	0.57	0.66	1
$1-R_f$	0.43	0.34	0
$k_{cleav,1}$[a]	2.54 min^{-1}	0.96 min^{-1}	0.31 min^{-1}
$k_{cleav,2}$[a]	0.14 min^{-1}	0.048 min^{-1}	0

$k_{cleav,avg}$	1.74 min^{-1}	0.65 min^{-1}	0.31 min^{-1}
$k_{ligat,1}$[b]	0.051 min^{-1}	0	3.1 min^{-1}
$k_{ligat,2}$[b]	0.0028 min^{-1}	0	0
$k_{ligat,avg}$	0.030 min^{-1}	0	3.1 min^{-1}
$k_{3p,dissoc}$	0	0	0
$k_{5p,dissoc}$	0	0	0
k_{tot}[c]	6.8 $M^{-1}s^{-1}$ 95% CI [6.37, 7.23]	2.48×10^{1} $M^{-1}s^{-1}$ 95% CI [2.18×10^{1}, 2.78×10^{1}]	1.09×10^{2} $M^{-1}s^{-1}$ 95% CI [9.10×10^{1}, 1.27×10^{2}]
k_{sca}[d]	5×10^{4} $M^{-1}s^{-1}$	1×10^{5} $M^{-1}s^{-1}$	1×10^{3} $M^{-1}s^{-1}$
k_{rel}[d]	1×10^{6} $M^{-1}s^{-1}$	1×10^{6} $M^{-1}s^{-1}$	1×10^{6} $M^{-1}s^{-1}$
k_{hyb}[e]	1×10^{6} $M^{-1}s^{-1}$	1×10^{6} $M^{-1}s^{-1}$	1×10^{6} $M^{-1}s^{-1}$
k_{ex}[e]	1×10^{6} $M^{-1}s^{-1}$	1×10^{6} $M^{-1}s^{-1}$	1×10^{6} $M^{-1}s^{-1}$
k_{trf}[f]	2.85×10^{2} $M^{-1}s^{-1}$ 95% CI [2.69×10^{2}, 3.00×10^{2}]	9.69×10^{2} $M^{-1}s^{-1}$ 95% CI [8.46×10^{2}, 1.09×10^{3}]	2.05×10^{1} $M^{-1}s^{-1}$ 95% CI [1.89×10^{1}, 2.22×10^{1}]
$k_{hyb,2}$[e]	1×10^{6} $M^{-1}s^{-1}$	1×10^{6} $M^{-1}s^{-1}$	1×10^{6} $M^{-1}s^{-1}$
$k_{hyb,3}$[e]	1×10^{6} $M^{-1}s^{-1}$	1×10^{6} $M^{-1}s^{-1}$	1×10^{6} $M^{-1}s^{-1}$

[a]Cleavage rates of nucleic acid enzymes were measured by our experiments (Supplementary Fig. 13). [b]Ligation rates were assumed based on cleavage rates. [a,b]The cleavage and ligation constants used here are effective kinetic parameters for the modeled catalyst-bound states and should not be interpreted as direct measures of the overall thermodynamic equilibrium constant of the uncatalyzed cleavage-ligation reaction. [c]The rate of substrate-mediated product displacement was fit to our experimental data (time-dependent cleavage rates with no scavenger) (Fig. 3C-D). [d]These rates were assumed based on prior experimental measurements[3-5]. [e]We set an arbitrary rate in hybridization. [f]The rate of substrate transfer from the scavenger to the ribozyme was fit to our experimental data (time-dependent cleavage rates with scavenger) (Fig. 3C-D).

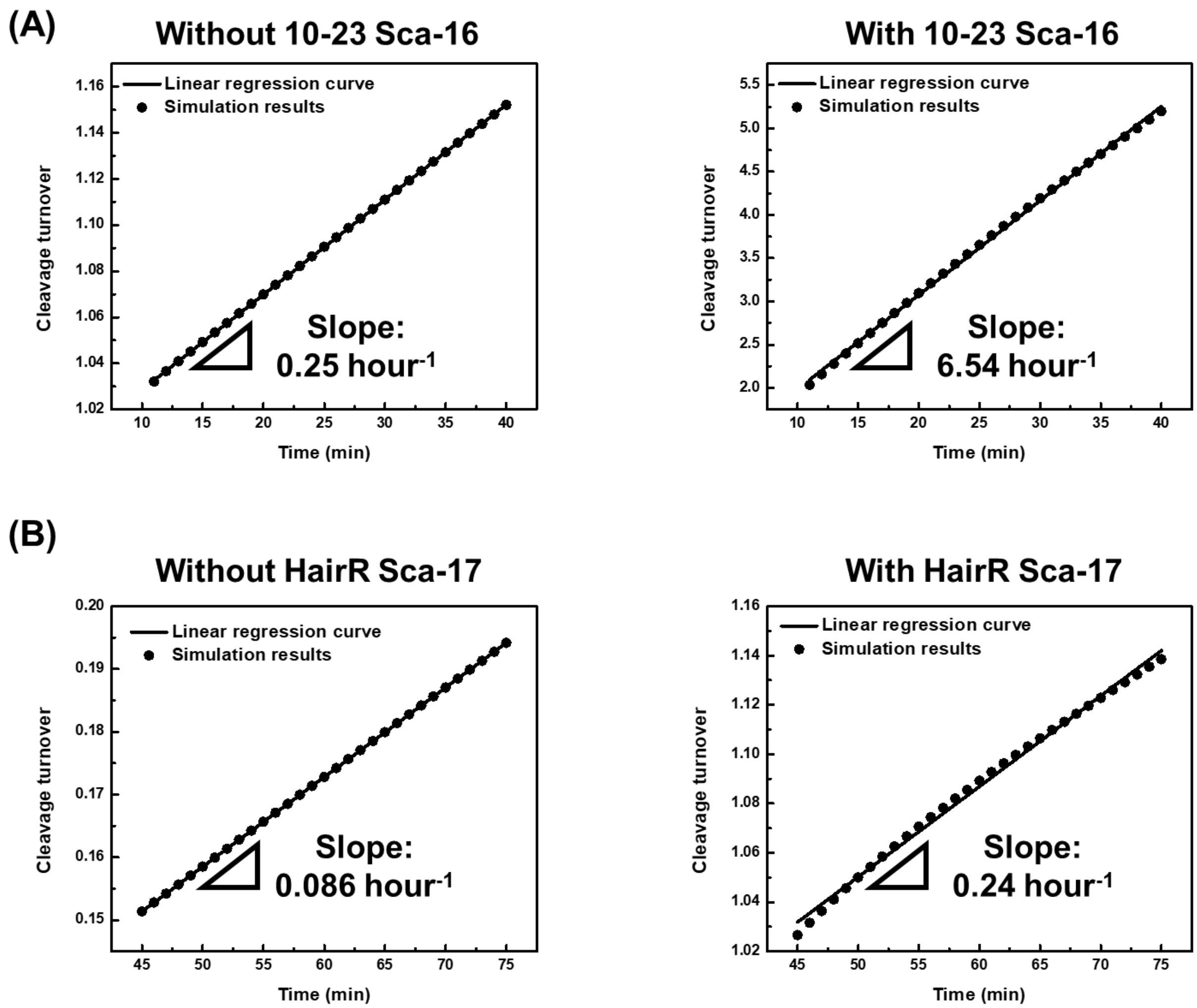

Supplementary Figure 14. Turnover frequency of two different nucleic acid enzymes with and without partner scavengers. Catalytic turnover rates of nucleic acid enzymes were predicted using the kinetic model described in Supplementary note 3 and the rate constants listed in Supplementary Table 4. (A) Predicted turnover frequency of 10-23 D-HBV without (left) or with 10-23 Sca-16 (right). (B) Predicted turnover frequency of HairR without (left) or with (right) with HairR Sca-17. The linear slope of each simulated trajectory was used to calculate turnover frequency.

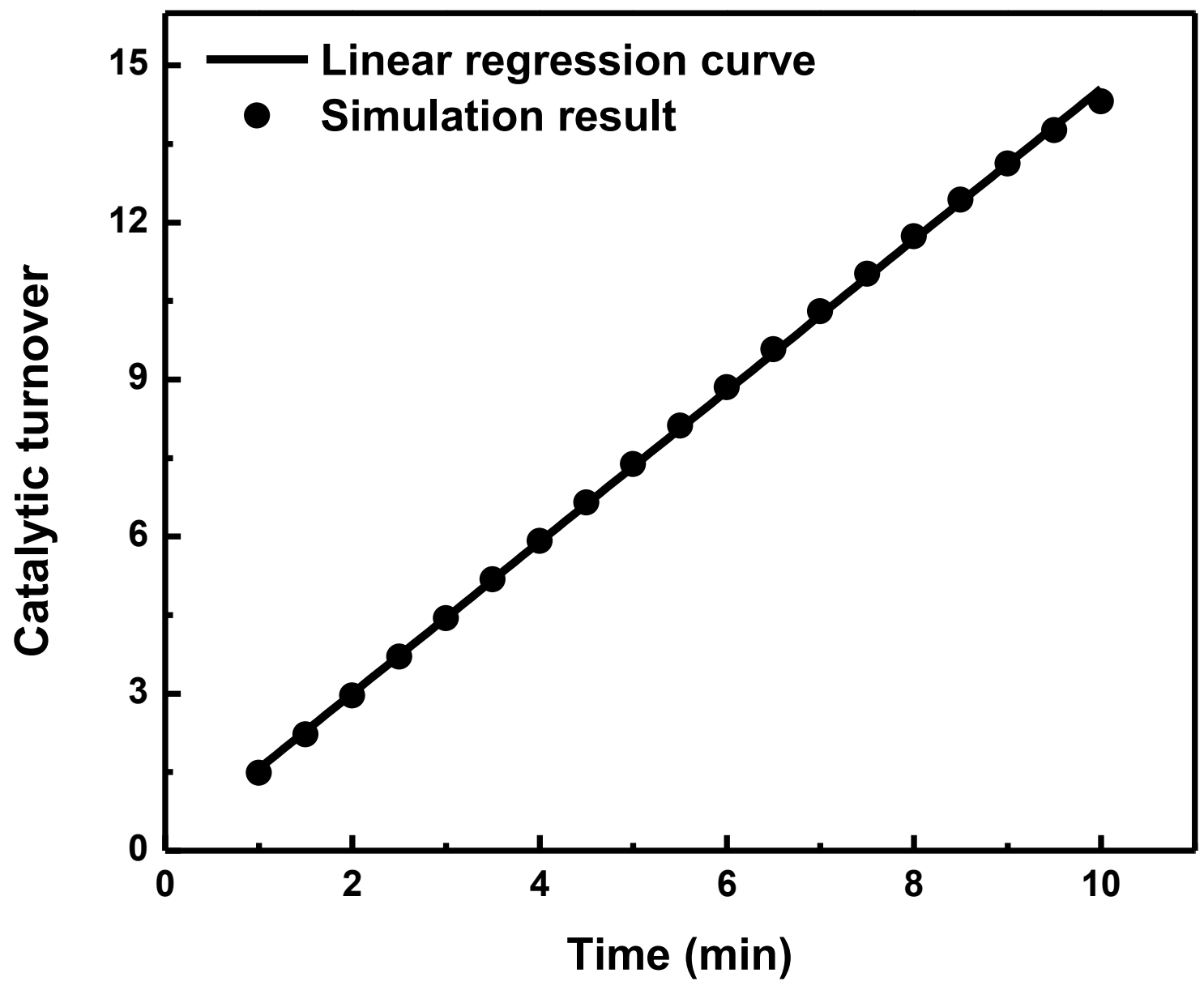

Supplementary Figure 15. Turnover frequency of HHR when there is no substrate scavenging. To assume no substrate scavenging, the hybridization rate between scavenger and substrate was set to 0 in our model. In this condition, catalytic turnover of HHR was predicted by our model, and the linear increase of catalytic turnover over time was used to calculate turnover frequency of HHR. We obtained the following linear regression curve, y = a + bx (a: 0.12, and b: 1.44). The R-squared value of the regression curve is 1.0. From the slope of the graph (1.44), turnover frequency was calculated to 1.44 min^{-1} (86.4 h^{-1}).

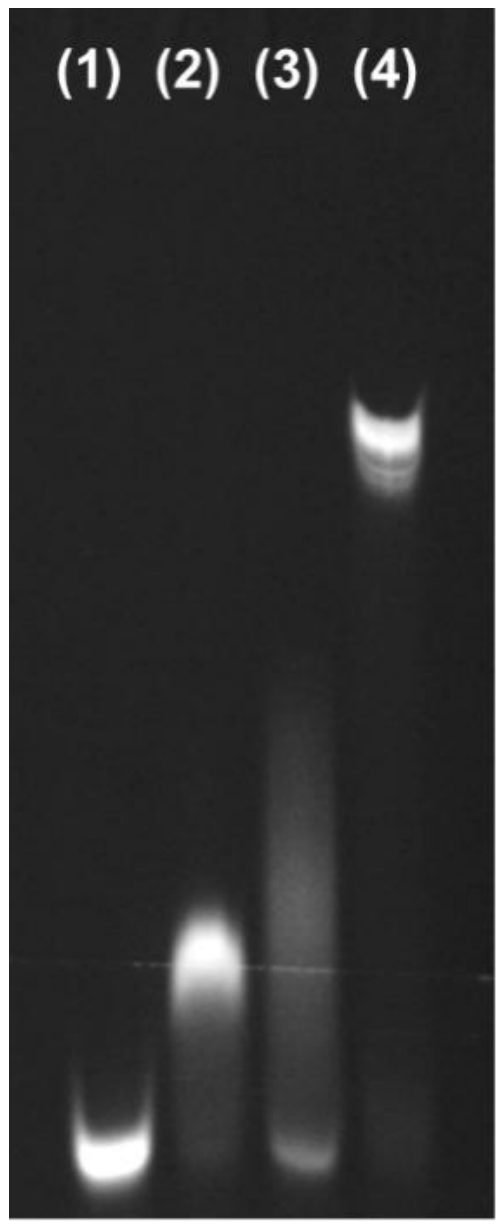

(1) NS1-Atto488
(2) NS1-Atto488 + NS2
(3) NS1-Atto488 + NS3
(4) NS1-Atto488 + NS2 + NS3

Supplementary Figure 16. Y-shaped DNA nanostar formation confirmed by gel electrophoresis. 10% PAGE-1X TBE gel at 4 °C was employed in this assay. When all three strands (NS1-Atto488, NS2, and NS3) were incubated together, they were successfully self-assembled to a high molecular weight complex given the result in gel analysis (lane 4). In sample preparation, the concentration of all DNAs and self-assembled structures was 10 μM in 20 mM Tris-HCl (pH 7.9) and 1.5 mM magnesium acetate. 5 μl of sample was mixed with 2 μl of 30% glycerol for gel electrophoresis. This experiment was performed in a single biological replicate.

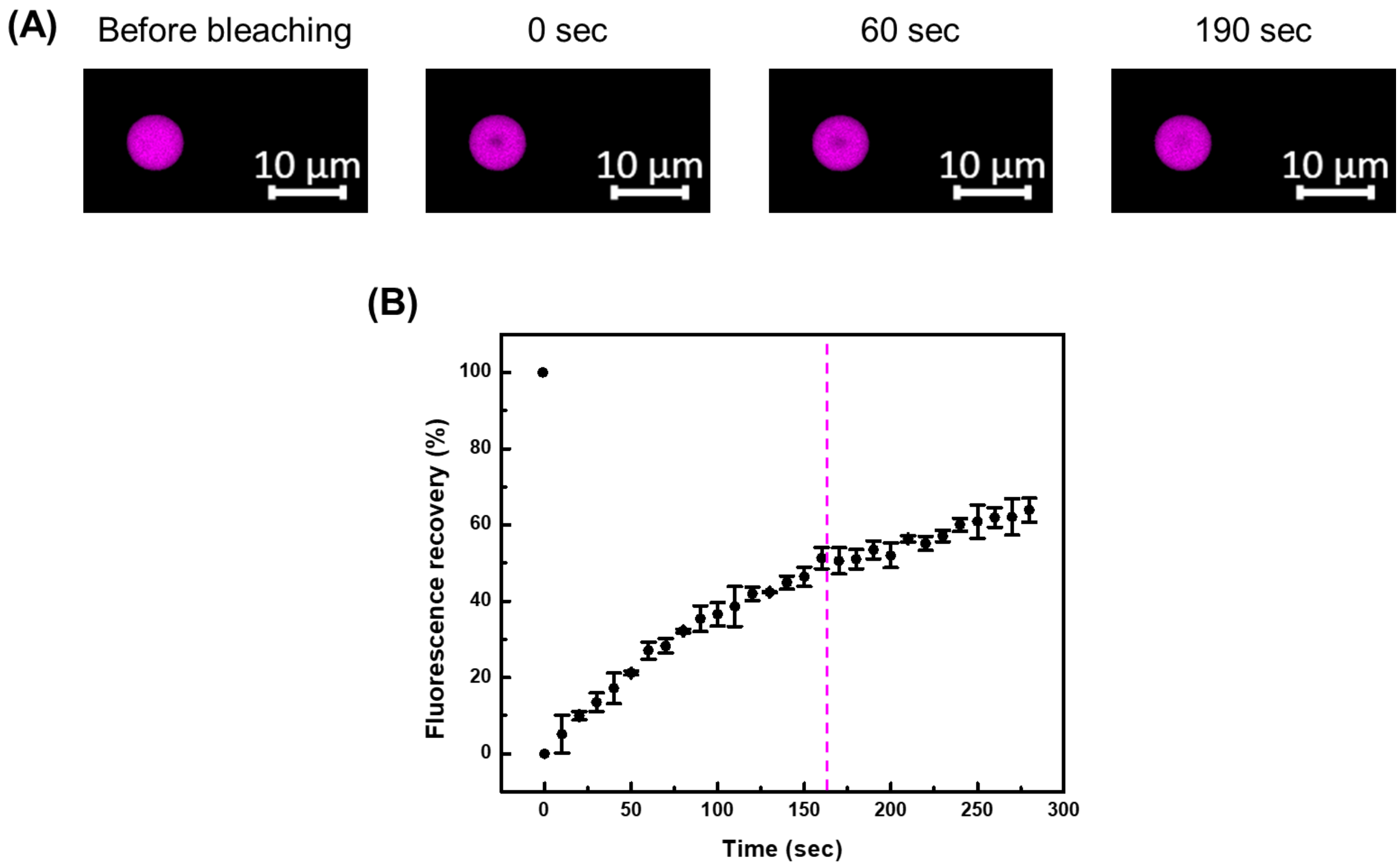

Supplementary Figure 17. FRAP analysis of Cy5-labeled DNA nanostar droplets at 37 °C using confocal microscopy. (A) Monitoring of Cy5 signals of DNA droplets before and after photobleaching. (B) Fluorescence recovery of photobleached DNA droplets over time. The magenta dashed vertical line is the time point at 50% recovery. The circular region used to bleach a droplet was used as the ROI to calculate the mean pixel intensity over time. The percent recovery was calculated according to the equation: $(F_t - F_0)/(F_{pre} - F_0) * 100$, where F_t is the mean fluorescence intensity at a measured timepoint, F_0 is the fluorescence in the ROI of the first image after photobleaching (0%), and F_{pre} is the fluorescence in the ROI of the image just before starting bleaching (100%). Three independent analysis was performed (n=3).

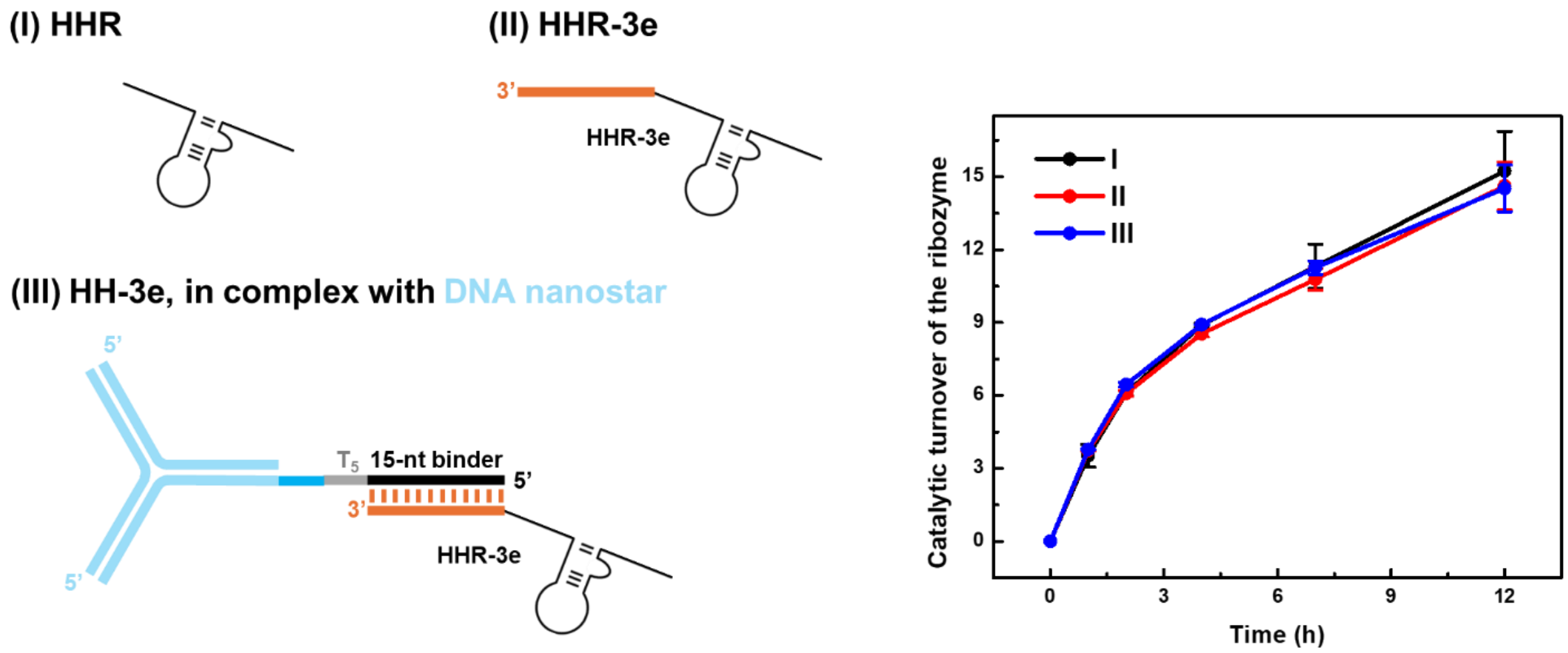

Supplementary Figure 18. Effects of 3' extension (II) and nanostar binding to the 3' extended domain (III) of normal hammerhead ribozyme (I) on catalytic turnover. As the DNA nanostar, we used DNA motif (consisting of NS1, NS2-BE, and NS3-BE), which has two blunt ends and one sticky end to prevent LLPS. 20 μM Sca-16 was also incubated in the reaction mixture (n=2).

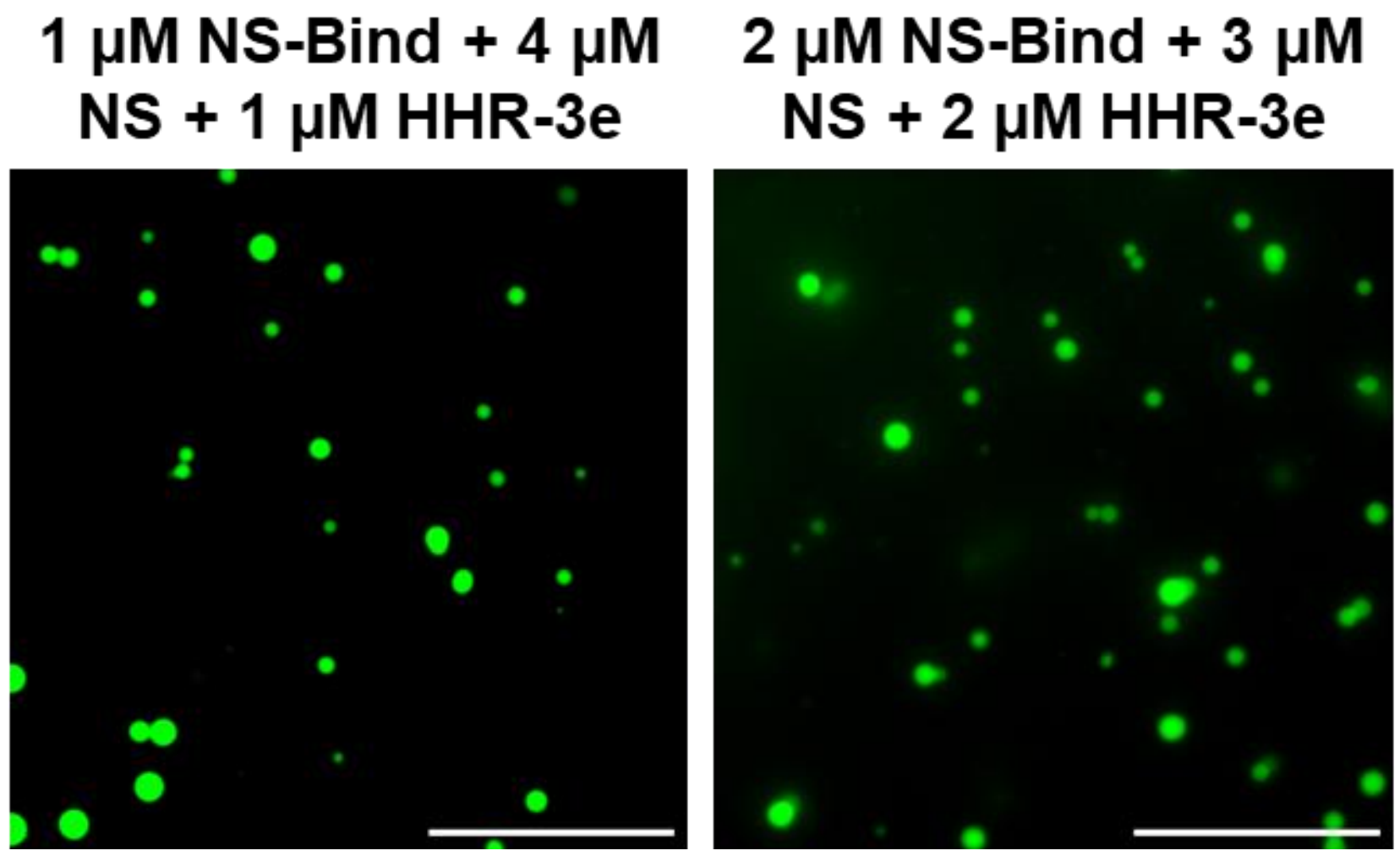

Supplementary Figure 19. Successful LLPS when NS-Bind, NS, and HHR-3e were incubated together. After incubation at 37 °C for 1 h, we stained the resulting solution (10 μl) with 500X SYBR Safe (0.4 μl) for 1 min. Then microscope images were immediately acquired. Scale bars: 50 μm.

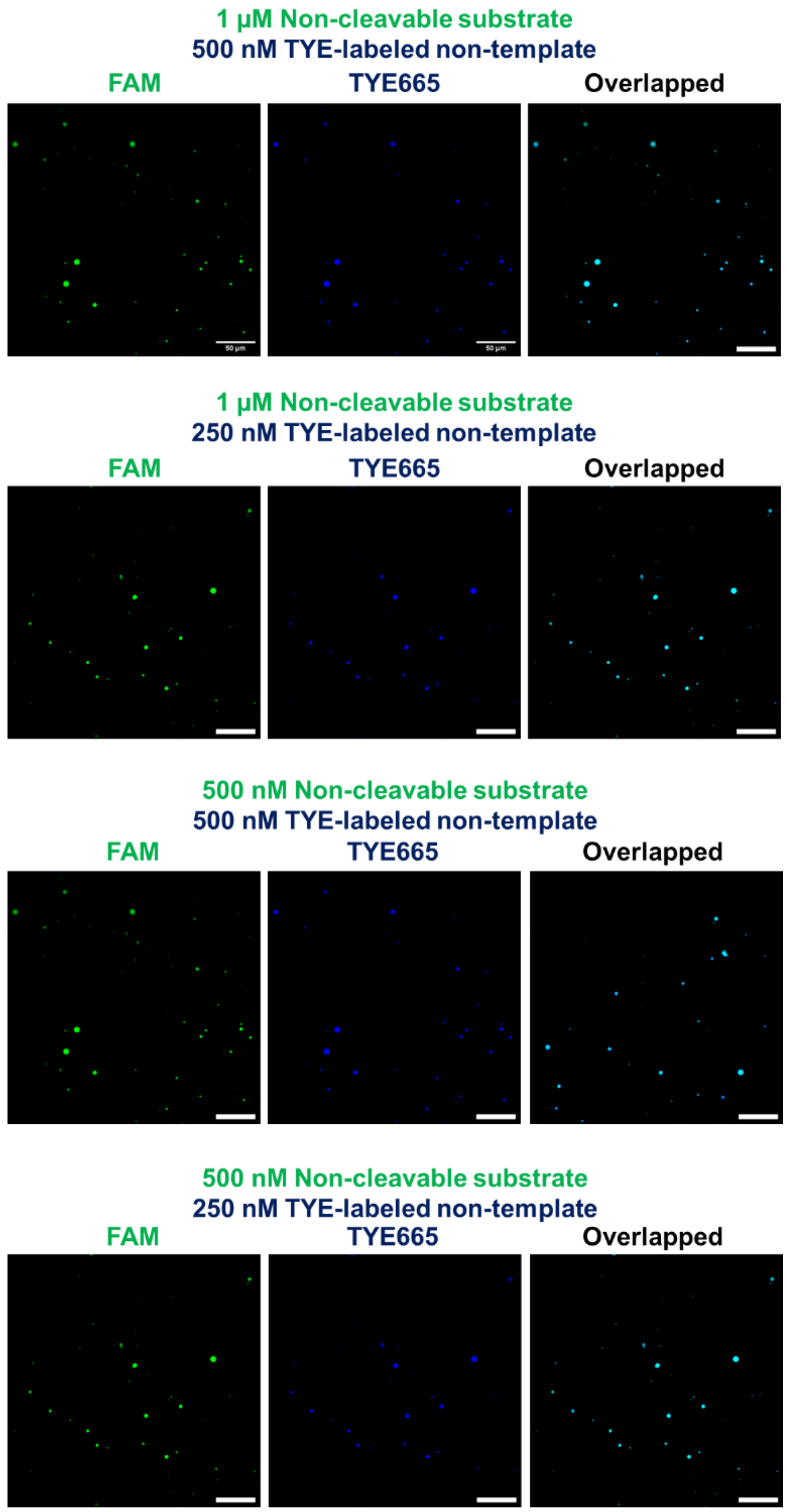

Supplementary Figure 20. Co-localization of hammerhead ribozymes and transcription templates in the dense phases. We found that FAM signals of non-cleavage substrate (NC-HHR Sub), which binds to the hammerhead ribozyme (HHR-3e), and TYE665 signals of nontemplate (nTemp-Sub-TYE665) that hybridizes with NS-txn, are co-partitioned within the same DNA droplets. Four different concentration combinations were used in this assay. The partition coefficient of non-cleavable substrates (1 μM) and non-

templates (500 nM) were measured as 62.35 ± 21.18 and 47.58 ± 34.39, respectively. Scale bars: 50 µm. Over 10 droplets were analyzed to obtain partition coefficients.

Supplementary Table 5. Experimental conditions to check the multiphase effects on cleavage turnover.

	(1)	(2)	(3)	(4)
NS	4.4 µM	4.9 µM	4.5 µM	5 µM
NS-Bind	0.5 µM	0	0.5 µM	0
NS-Bind-BE[a]	0	0.5 µM	0	0.5 µM
HHR-3e	0.5 µM			
NS-template	0.1 µM	0.1 µM	0	0
NS-template-BE[a]	0	0	0.1 µM	0.1 µM
nonTemplate	0.1 µM			
Sca-16	10 µM			
YIPP	0.00135 units/µl			
RC buffer	1X			
T7 RNAP	4 units/µl			

[a]NS-Bind-BE and NS-template-BE have two blunt ends and one sticky end.

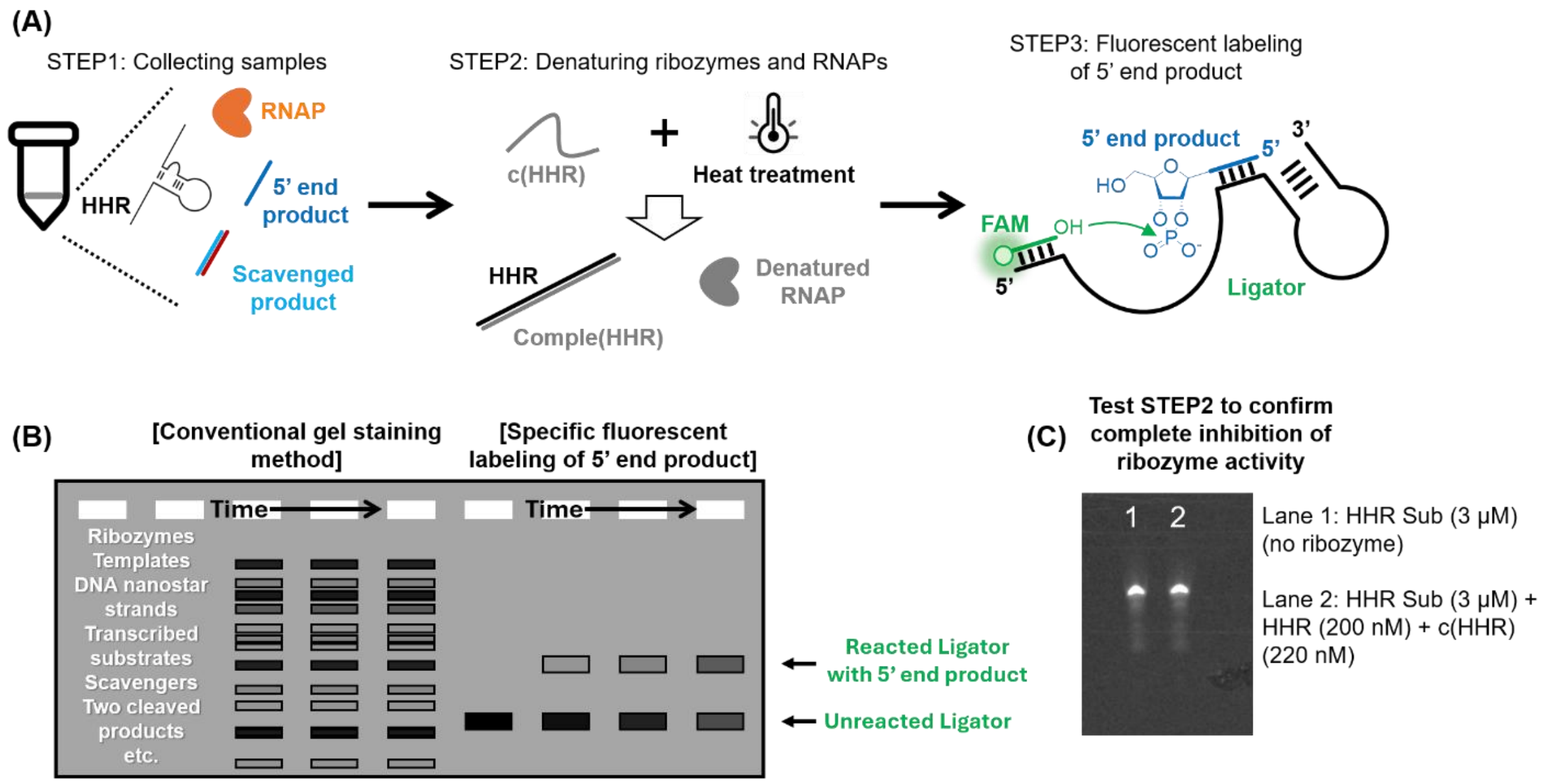

Supplementary Figure 21. Specific fluorescent labeling of ribozyme-catalyzed products. (A) Reaction mixture was collected at a desired time point (STEP1), and it was immediately mixed with c(HHR) strand, which is fully complementary to the HHR followed by heat treatment (90 °C for 5 min, then cooling to 25 °C at a rate of -0.1 °C/sec) deactivating both ribozymatic and transcriptional reactions (STEP2). The 5'P generated by HHR possesses as 2'-3' cyclic phosphate moiety, which was directly utilized for fluorescent labeling (STEP3). A Ligator complex, consisting of 3'-FAM-labeled RNA and a hairpin ligase ribozyme, was incubated with the collected sample overnight to chemically conjugate the 5' OH group of the Ligator to the 2'-3' cyclic phosphate of the 5'P.[8,9] (B) Conventional gel staining method using SYBR Gold will stain every component in our reaction condition, including DNA nanostar strands, template duplexes, transcribed substrates, cleaved substrates, *etc*. This will not allow specific identification of cleaved product formation. However, use of specific fluorescent labeling makes it possible to measure the relative accumulation of the 5'P. (C) Control experiment confirming inhibition of HHR by the complementary strand, c(HHR). HHR Sub was incubated either without ribozyme or with HHR pre-complexed with 1.1X c(HHR) under the same buffer and heat-treatment conditions. No detectable substrate length change was observed in either sample, indicating that the complementary strand completely inhibits HHR-mediated substrate cleavage under these conditions. The experiment was performed in duplicate. Chemical structures in this figure were drawn by ChemDraw 23.1.1.

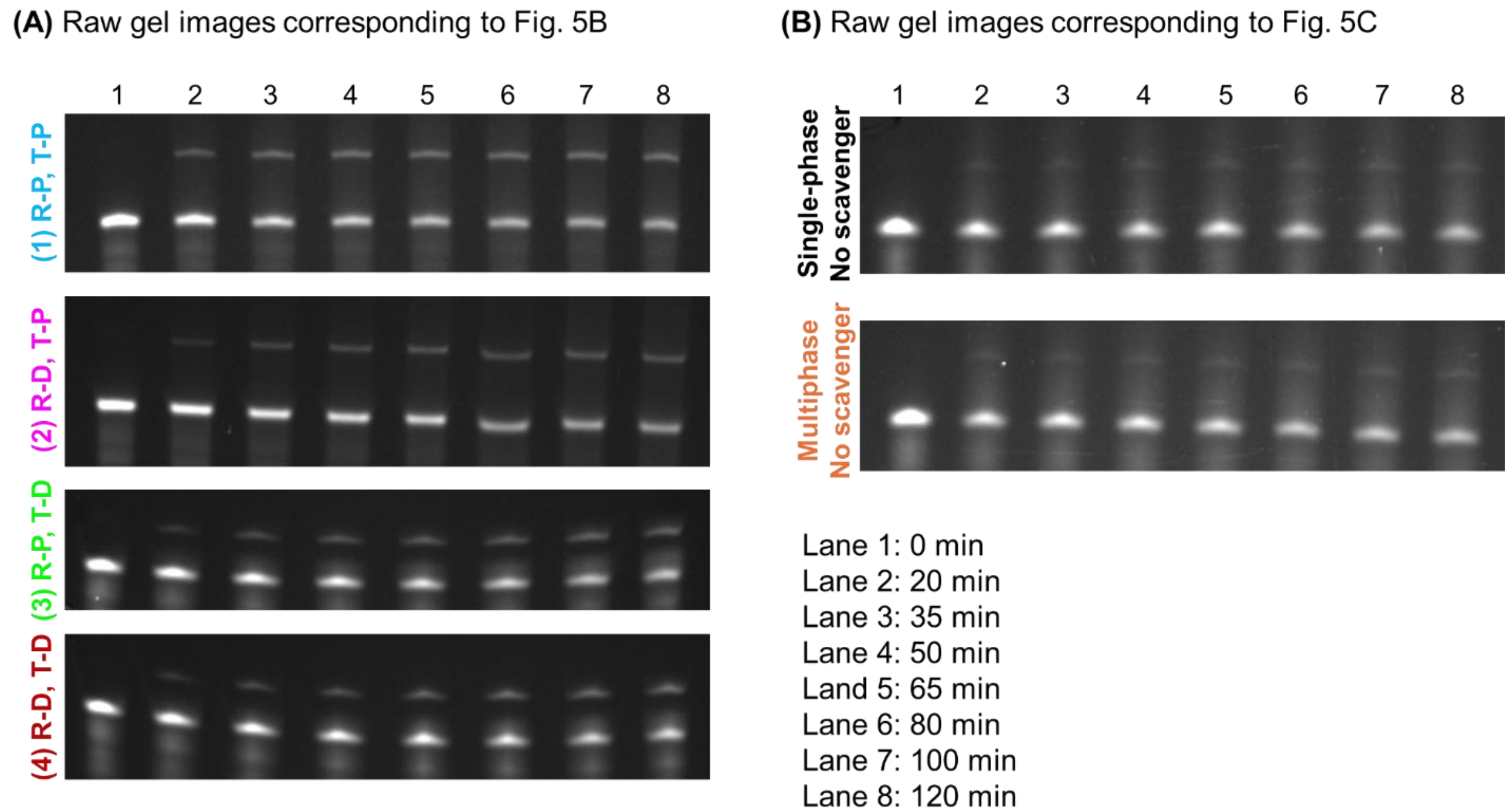

Supplementary Figure 22. Raw gel images corresponding to Fig. 5B-C. (A) Raw gel images showing fraction of downstream Ligator (FAM-labeled) reacted at fixed time points (0-120 min), corresponding to Fig. 5B. (B) Raw gel images corresponding to Fig. 5C. In both gel images, the lower band indicates unreacted Ligator and the upper one indicated reacted Ligator.

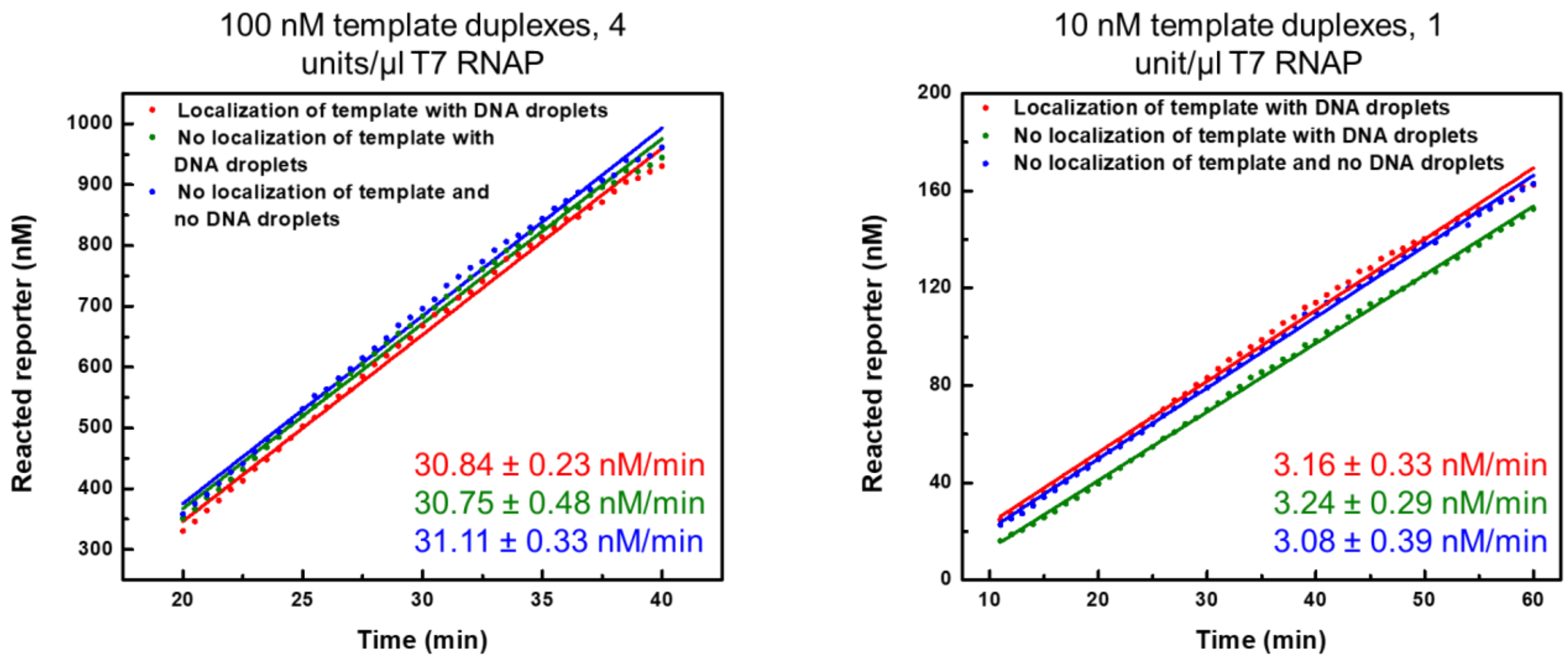

Supplementary Figure 23. Measured transcriptional rates are indistinguishable with and without DNA droplets and whether or not the template is localized to the droplets. We prepared three different conditions: (1) Template localized to DNA droplets, (2) No localization of template in the presence of DNA droplets by using NS-template-BE, and (3) only NS-template-BE with no DNA droplets. Here, 1 µM of a reporter complex, consisting of HHR Sub-Rep-3Q and HHR Sub-Rep-5F was employed to measure transcriptional rates by measuring the change in fluorescence as the RNA transcript separated the complex, and therefore the Cy3 dye on HHR Sub-Rep-5F from the quencher on HHR Sub-Rep-3Q. Because transcription, rather than strand displacement is rate limiting, the linear slope of each curve is reported as the transcription rate of the substrate for the ribozyme.

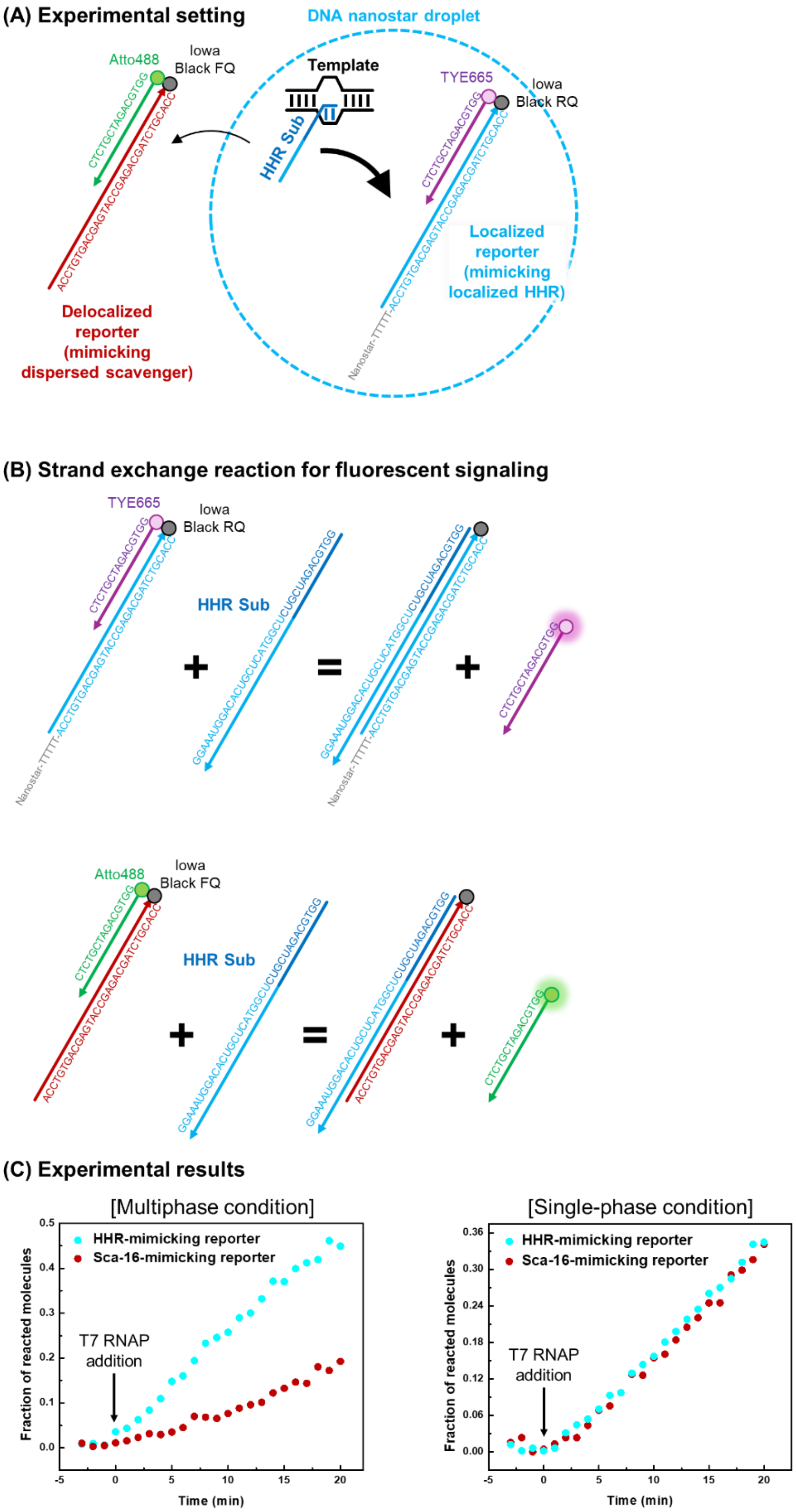

Supplementary Figure 24. Experimental demonstration of multiphase-assisted control of binding reaction pathway. (A) Schematic illustration of binding-pathway canalization in

a multiphase system. Here, one reporter complex (500 nM; Localized Rep-3Q and Localize Rep-5F (Supplementary Table 1)) was co-localized with a template to mimic localized HHR, and another reporter complex (500 nM; Dispersed Rep-3Q and Dispersed Rep-5F) was dispersed in the reaction solution to mimic dispersed scavenger. The transcribed HHR Sub is expected to bind the localized reporter rather than the dispersed reporter. (B) Reporting mechanisms of two reporter complexes upon binding to HHR Sub. Binding of HHR Sub displaces a fluorophore-labeled strand from a reporter complex, generating a fluorescence signal that enables quantification of HHR Sub binding to each reporter. (C) Kinetic profiles of the reporters' fluorescence over 20 min after T7 RNAP (4 units/μl) was added in multiphase and single-phase systems, respectively. In the multiphase (left), the transcribed HHR Sub preferentially binds the localized reporter, whereas in the single-phase system (right) it binds the two reporters with no preference.

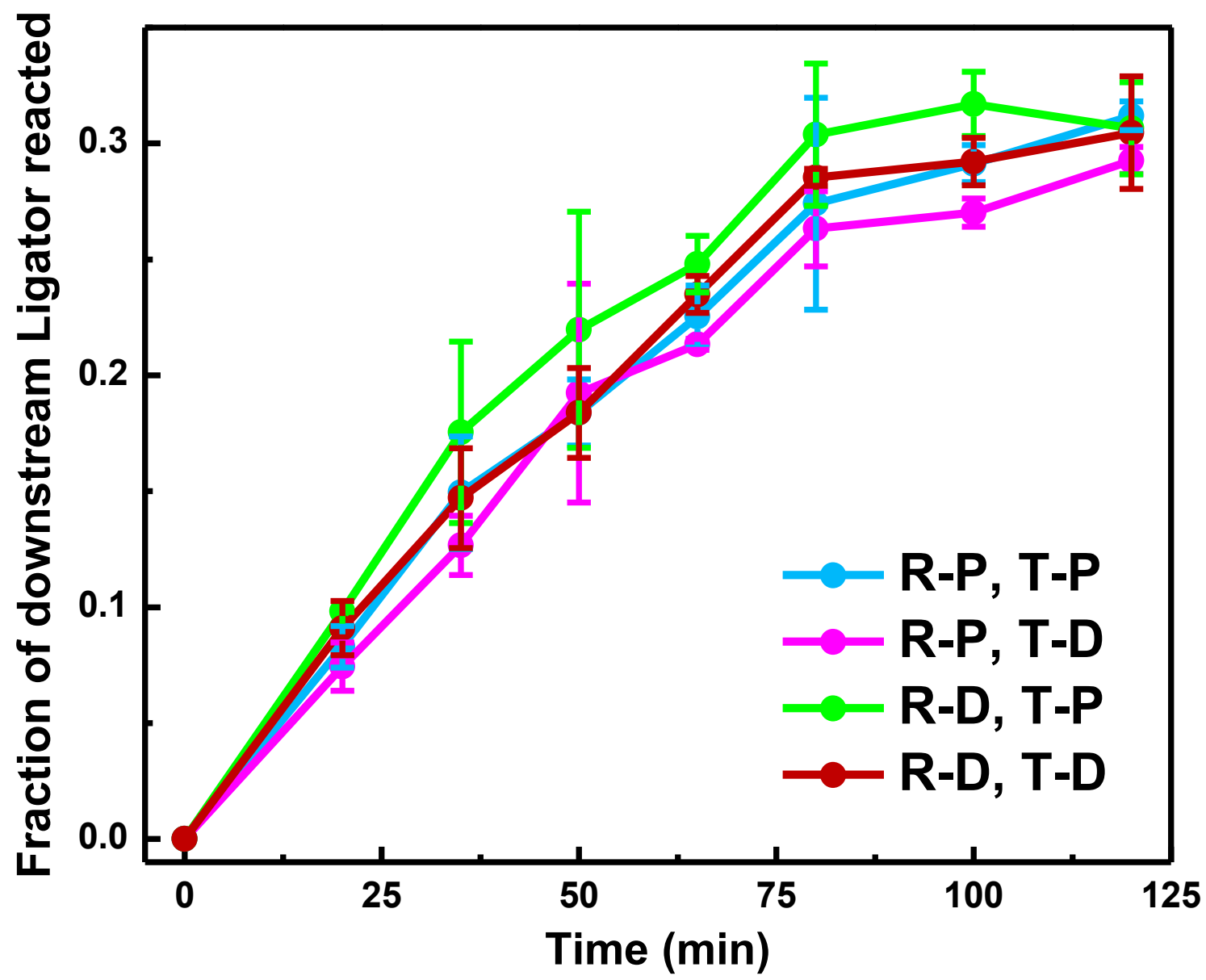

Supplementary Figure 25. Effect of a multiphase reaction system on ribozyme catalysis in the presence of excess Scavenger (100 μM). Here, P means partitioning within DNA nanostar droplets, and D means no localization as in main text Fig. 5B. All four different partitioning conditions showed similar fractions of Ligator reacted over time, meaning that co-partitioning of templates and ribozymes does not accelerate ribozyme catalysis when an excess of scavengers is present. This lack of acceleration is presumably due to the fact that when this very high concentration of Scavenger is used, the concentration of ribozyme inside the dense phase no longer exceeds that of the scavenger in the dense phase, so that substrate scavenging is no longer mitigated. This experiment was performed in duplicate.

Supplementary note 5. Quantification of HHR-mediated cleavage when using a ligation-based assay

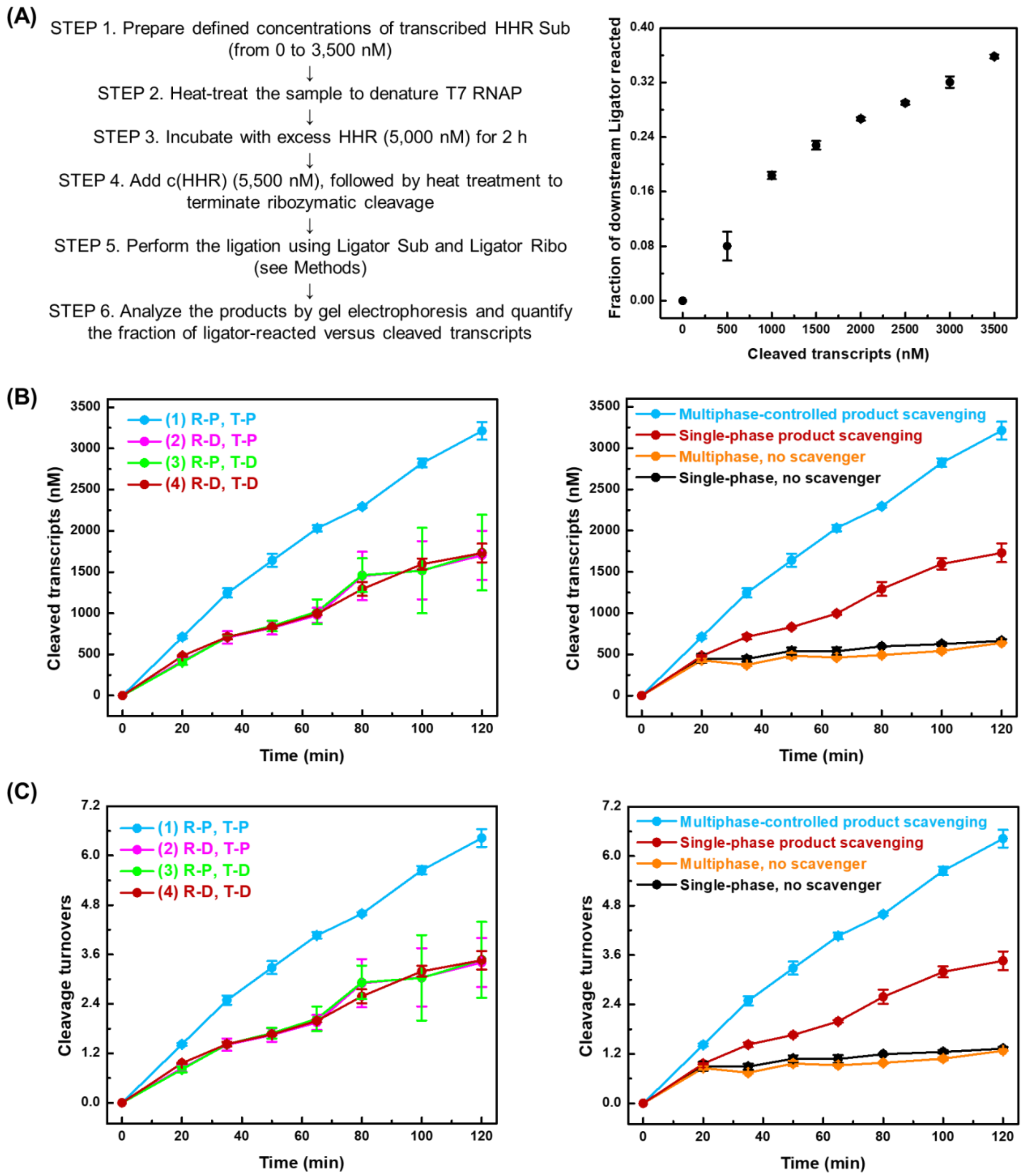

Supplementary Figure 26. Quantification of HHR cleavage of transcribed HHR Sub, and calculation of cleaved transcripts (nM) and cleavage turnovers over time. (A) Workflow for quantifying HHR cleavage using a downstream ligation reaction (left) and representative result (right). This experiment was performed in duplicate. (B) Cleaved

transcripts (nM) versus time (min), corresponding to Fig. 5B (left) and Fig. 5C (right). (C) Cleavage turnovers versus time (min), corresponding to Fig. 5B (left) and Fig. 5C (right).

To quantify HHR-mediated cleavage of transcribed HHR Sub, we performed the assay shown in Supplementary Fig. 26A (left). Based on the measured transcription rates (Supplementary Fig. 23), we first prepared defined solutions containing defined concentrations of transcribed HHR (0, 500, 1000, 1500, 2000, 2500, 3000, and 3500 nM). These solutions were heat-treated to denature T7 RNAP. Excess HHR (5 μM) was then added to each of the solutions to cleave all transcripts. To terminate ribozyme activity, c(HHR) was then added, and the solutions were heat-treated, after which Ligator Sub and Ligator Ribo (Supplementary Table 1) were added to initiate the downstream ligation reaction (see Methods). The fraction of ligator reacted as a function of cleaved transcript concentration was then quantified by denaturing gel electrophoresis (Supplementary Fig. 26A, right).

For quantification, the calibration data were used to construct an empirical relationship between cleaved transcript concentration and the fraction of downstream Ligator reacted. The calibration curve was generated using piecewise cubic Hermite interpolating polynomial (PCHIP) interpolation. We then used the calibration curve to estimate the concentrations of cleaved transcripts and the corresponding cleavage turnover over time for the conditions shown in Fig. 5B-C.

To quantitatively compare cleavage rates between MCPS and single-phase product scavenging (Fig. 5B-C and Supplementary Fig. 26B-C), we used a simple model in which cleavage and transcription are coupled:

$$(Equation\ 3.1)\ \frac{d[S]}{dt} = v_{transcription} - k_{obs}[S]$$

$$(Equation\ 3.2)\ \frac{d[P]}{dt} = k_{obs}[S]$$

Here, [S] is the concentration of uncleaved transcript, [P] is the concentration of cleaved product, and $v_{transcription}$ is the transcription rate, taken as 30 nM/min from Supplementary Fig. 23. The initial conditions were S(0)=0 and P(0)=0.

After 2 h reaction (Supplementary Fig. 26B, cyan line), the MCPS condition yielded 3224 nM cleaved products and 376 uncleaved transcripts. However, when the template, HHR, and Sca-16 were dispersed (red line), 1714 nM cleaved products and 1886 nM uncleaved transcripts were measured. Using these endpoint values, k_{obs} was estimated to be 0.079 min^{-1} for MCPS and 0.012 min^{-1} for the dispersed condition. These results

indicate that the multiphase system accelerated k_{obs} by 6.5-fold, proving effectiveness of pathway canalization by compartmentalization.

Supplementary references

1. Zadeh, J. N., Steenberg, C. D., Bois, J. S., Wolfe B. R., Pierce M. B., Khan, A. R., Dirks R. M. & Pierce N. A. NUPACK: analysis and design of nucleic acid systems. *J. Comput. Chem.* **32**, 170-173 (2011).
2. Zhang, D. Y. & Winfree, E. Control of DNA strand displacement kinetics using toehold exchange. *J. Am. Chem. Soc*. **131**, 17303-17314 (2009).
3. Todisco, M. & Szostak, J. W. Hybridization kinetics of out-of-equilibrium mixtures of short RNA oligonucleotides. *Nucleic Acids Res*. **50**, 9647-9662 (2022).
4. Liu, H., Hong, F., Smith, F., Goertz, J., Ouldridge, T., Stevens, M. M., Yan, H. & Šulc, P. Kinetics of RNA and RNA:DNA hybrid strand displacement. *ACS Synth. Biol*. **10**, 3066-3073 (2021).
5. Smith, F. G., Goertz, J. P., Jurinović, K., Stevens, M. M. & Ouldridge, T. E. Strong sequence–dependence in RNA/DNA hybrid strand displacement kinetics. *Nanoscale* **16**, 17624-17637 (2024).
6. Banerjee, D., Tateishi-Karimata, H., Ohyama, T., Ghosh, S., Endoh, T., Takahashi, S. & Sugimoto, N. Improved nearest-neighbor parameters for the stability of RNA/DNA hybrids under a physiological condition. *Nucleic Acids Res.* **48**, 12042-12054 (2021).
7. Sugimoto, N., Nakano, S., Katoh, M., Matsumura, A., Nakamuta, H., Ohmichi, T., Yoneyama, M. & Sasaki, M. Thermodynamic parameters to predict stability of RNA/DNA hybrid duplexes. *Biochemistry* **34**, 11211-11216 (1995).
8. Fedor, M. J. Structure and function of the hairpin ribozyme. *J. Mol. Biol*. **297**, 269-291 (2000).
9. Yadava, R. S., Choi, A. J., Lebruska, L. L. & Fedor, M. J. Hairpin ribozymes with four-way helical junctions mediate intracellular RNA ligation. *J. Mol. Biol*. **309**, 893-902 (2001).
