# Supplementary material for: Reaction enhancement via product scavenging and phase separation-driven pathway canalization
Source: Nat Commun. 2026 Sep 28;17:11174. doi: 10.1038/s41467-026-77899-x (PMC13714313; doi:10.1038/s41467-026-77899-x)
Supplement: Supplementary file 3 [file 41467_2026_77899_MOESM3_ESM.pdf]

nature portfolio

Peer Review File

Reaction enhancement via product scavenging and phase separation-driven pathway canalization

Corresponding Author: Professor Rebecca Schulman

This file contains all reviewer reports in order by version, followed by all author rebuttals in order by version.

Version 0:

Reviewer comments:

Reviewer #1

(Remarks to the Author)
Kang et al present a mechanism for enhancing the rate with which a nucleic acid catalyst turns over its nucleic acid substrates. The system consists of a RNAzyme that cleaves a specific target RNA strand, but whose turnover is limited by the fact that both halves of the target remain bound to the DNAzyme after cleavage. The authors introduce a scavenging strand that displaces one of the products, allowing enhanced turnover. However, the scavenger also tends to bind to the uncleaved substrate, slowing down the initial binding. On balance, the effect still accelerates turnover, and this effect is enhanced when catalysts are co-localised with the production of the RNA strands in a phase-separated system. The authors also show that their approach works for two other RNA-cleaving RNAzymes.

Overall, the principle is widely interesting and novel (although note comment 1a below). The results are clear. There are a number of issues with the way the results are presented and discussed, outlined below, that should be fixable. A higher-level criticism relates to design choices in the system; it isn't clear to me that these catalysts *need* to operate in product inhibited context, and it's not obvious that the designed scavengers are particularly well designed, given the catalyst and product combination, to circumvent inhibition. These factors don't invalidate the results, which do show the effect claimed, but it would be good to explore these factors more thoroughly.

Major comments:
1. Catalyst design. The authors are accelerating turnover in a cleavage reaction. Cleavage is actually the type of catalysis that is most immune to product inhibition, since the separated products will no longer cooperatively bind to the template -- they will detach *far* faster than the substrate. In order to experience product inhibition, therefore, both halves of the product need to be quite long (10/11 nt here).

(a) In line 87, the authors say that product inhibition is common in vivo because "substrates and products often differ by only a single cleavage-ligation event". But cleavage and ligation are actually massive changes! Cleaving a 21mer into a 10mer and an 11mer turns detachment from something that will never happen on feasible timescales to something that takes ~10 hours, according to the results. Slightly (a couple of base pairs) shorter arms would lead to quick detachment of the products, while the substrate would still stick extremely strongly. By contrast, if the catalyst is acting to template ligation, product inhibition becomes an unavoidable and enormous challenge, because the products tend to stick to the catalysts more strongly than the substrates due to cooperativity. The authors should explain these concepts more carefully in the introduction/discussion. They might note that recent work has explored how product inhibition can be overcome in DNA templating of (non-covalent) association [https://www.nature.com/articles/s41557-025-01831-x], and even introduced a mechanism based on a scavenger-like strand to accelerate it, although here the scavenger only binds to the product after it is formed [https://www.biorxiv.org/content/10.64898/2026.02.18.706517v1.abstract - this latest work was published online after the paper under consideration was submitted, and so does not compromise its novelty].

(b) As the authors note on line 167, shorter recognition domains can be used, which would mitigate product inhibition in cleavage. They say that this speedup comes at the cost of specificity and affinity, but I am not sure about this argument. On the thermodynamics of base pairing alone (ignoring whether reduction in recognition domain length compromises catalytic activity once bound), there is surely a fairly large window in which the substrate, which binds cooperatively via two arms, will have strong affinity for the catalyst but the products, which do not bind cooperatively, will not. The authors cite the fact that the HDV ribozyme requires 15-16nt of recognition - but this is quite a lot less than the 21 used here, enough to (probably)

eliminate product inhibition as a significant effect. The claim about specificity is also not clear to me. A longer recognition domain will be more tolerant of a mismatched base pair, not less, according to the nearest neighbour model. At the very least, more discussion of why particular recognition domain lengths were chosen is required.

2. Scavenger design. The authors design the default scavenger to do a form of toehold exchange, leaving a 2 base-pair "output" toehold for the catalyst with the product fragment that is removed. This isn't discussed much in the text and the diagrams don't usually show these free bases. But this toehold is potentially very important, if the rate limiting step is really the reaction between catalyst bound to half the product and a "scavenged" substrate, as suggested by Fig. 1b. One would surely expect this reaction to proceed via this toehold? The reaction rate observed is on the right order of magnitude.

The authors don't discuss this rate limiting step in detail, but they do give some alternative designs in the SI. However, these designs only consider shortened versions of the output toehold (and shortened versions of the input toehold). Surprisingly to me, the strands with a shortened output toehold are not that much worse at accelerating the reaction, although they are a bit worse. However, what I really don't understand is why the authors didn't try an increased (3,4 base) output toehold. It might make the scavenging a bit slower, but the rate limiting step is binding of the scavenged substrate, so this is surely a price worth paying?

At the very least, the authors should discuss the design of the scavenger probes in a bit more detail, focussing on the question of toehold exchange. Why was the design selected? Why were longer output toeholds not tested? In their position I would probably test the efficacy of a longer output toehold (Sca-15' and Sca-14').

3. Theoretical modelling. It is good that the experimental results are supported by theoretical modelling, but aspects of it are very unclear.

(a) How were the two cleavage rates handled in the analysis? In Supplementary note 1, the two cleavage rates just appear together in the equation, which will be equivalent to a single process with a rate equal to the sum of the two - this will not yield the observed two-phase behaviour. The same applies to the two ligation rates used.

(b) It is also unclear why the replacement of the two products with the substrates is modelled as a single transition with a rate depending on [S] and [ScR]. I can see how something like this might emerge from a more detailed description, but it isn't really justified.

4. The thermodynamic analysis (Figure S5 and Table S2) in the SI is very confusing. First, the Sugimoto parameters can be used to predict the stability of DNA/RNA duplexes. It is not always true that DNA:RNA is more stable than DNA:DNA, it depends on the balance of purines in the RNA strand. Second, the caption of Table S2 is very hard to read and understand. Finally, I am confused as to why the percentage of HHR bound to HHRsub increases as the amount of Sca16 increases in the top table.

Minor Comments:

5. Is the term "enzyme" typically used for RNAzymes?

6. Figure quality is sometimes not great. The images have artefacts from conversion to PDF, and the nucleic acid strands are shown with kinks in them even when they are part of duplexes, which is strange.

7. Line 201 - does "predicted" mean "estimated based on fits"?

8. Lines 235-236 need rephrasing for clarity.

9. I think it would be very beneficial to come up with an abbreviated, consistently-used nomenclature for the species in the system, it's clunky to keep saying eg. "3' end product" and complexes described in this way are particularly awkward.

10. The definitions of the parameters and reactions in the models are very hard to follow from the text. Is it possible to illustrate them in the main text itself?

11. I couldn't follow lines 271-272 - the substrate isn;t transferred fro the scavenger to the "complex", surely? It disrupts the complex.

12. Why is cleavage/ligation site used in Fig3d but not in the others?

13. 322-323 The ratio of ligation/cleavage rates is determined by the thermodynamics of the reactants and the products, not the catalyst.

14. In Fig 4D the text is low quality.

15. Line 356-357 is confusing. It sounds like 86.4 is the measured frequency, but I think you mean this is the maximum possible?

16. Line 373 - what precisely is meant by LLPS droplets "mix rapidly"?

17. Line 415 - the evidence presented is that the scavenger doesn't act as a surfactant - is this the same as saying it doesn't interact with the droplets?

18. Line 507 - is the multiphase acceleration dramatic? It's a factor of 2.

19. Line 635 - was it tested whether this complementary strand inhibited the HHR? I agree it should.

20. Line 95 of SI - I think different notation is being used from what is used in the equations themselves.

21. The caption of Figure S3/S9/S13 could be a bit more explicit about what it is describing. Simulation of what?

(Remarks on code availability)

Reviewer #2

(Remarks to the Author)
The authors present an exciting strategy to overcome product inhibition, a fundamental bottleneck in catalysis where slow product dissociation limits turnover. By using nucleic acid enzymes as models, they demonstrate that a designed affinity reagent—a scavenger—can actively displace products via toehold-mediated strand exchange. This "product scavenging" increases turnover up to 39-fold. To address the inevitable "substrate scavenging" (where the scavenger binds to the structurally similar substrate), the authors utilise DNA nanostar-based condensates to co-localize enzymes and substrates to achieve catalytic rate enhancement of approximately 70-fold.

This work is an excellent demonstration of using predictable base-pairing rules to engineer novel biochemical pathways. The integration of thermodynamic displacement (scavengers) with spatial control (LLPS) is a modern approach to further control such reaction pathways. However, several points regarding the energy requirements, the scope of generalisation, and the clarity of the proposed mechanisms require further clarification. Therefore, I would like to ask for reconsideration of the paper after the following points have been addressed.

Major Points
- The "Energy Cost" of Scavenging: The introduction of a scavenger to "clear" the product requires the addition of a high-energy species (the free scavenger strand) which ends up in a lower-energy state (the scavenger-product duplex). While the catalytic rate is enhanced, the overall system is "driven" by the chemical potential of the scavenger. Could the authors provide a more detailed discussion on the efficiency/trade-off for this system? Specifically, how does the "cost" of providing the scavenger compare to the benefit of accelerated turnover in a potential therapeutic or diagnostic setting?
-
- Evaluation of the "Generalizability" Claim: The manuscript claims MCPS is a "generalizable and programmable framework". While this is clearly shown for three distinct nucleic acid enzymes, the jump to protein-based enzymes mentioned in the Discussion is non-trivial. While base-pairing rules (NUPACK) allow for precise scavenger design, for protein enzymes, finding affinity reagents (antibodies/aptamers) that bind products with higher affinity than the enzyme—without blocking the active site during the reaction—is significantly harder.

- As noted, Figure 1B introduces "scavenged substrate" and "catalyst with one product," which are absent from the "ideal" cycle in 1A . To improve coherence, I suggest updating Figure 1A to show the intermediate state (catalyst bound to one product) after the first product dissociates, as this better reflects the actual mechanism described later in Figure 2.

- Providing a "full cycle" diagram in the Supplementary Materials that tracks the exact fate of the catalyst in the presence of scavengers.

- In a real-world "ideal" reaction, the binding of a new substrate might actually help "push" the old product off the enzyme (associative mechanism). Does the scavenger-mediated displacement compete with or synergise with the natural substrate-assisted dissociation? The Figure 1A "ideal" scheme should be updated to clarify if the "ideal" refers to zero inhibition or the specific MCPS mechanism.

Minor comments:
If excessive scavenger binding to the substrate is the bottleneck, could the authors comment on a "multi-layer" scavenging system? For example, using a small amount of "Primary Scavenger" and "Secondary Scavenger" that clears the primary scavenger from the product-scavenger complex. Would this allow for catalytic use of the scavenger itself?
Asymmetric Toeholds: Could adding shorter, reversible toeholds on the scavenger increase the rate of substrate transfer from the "scavenged substrate" back to the ribozyme?
Figure 2E Labeling: The placement of the "Scavenger" label on top of the thick red arrow in Figure 2E is slightly misleading, as it might be mistaken for a reaction condition rather than a physical species.

(Remarks on code availability)

Reviewer #3

(Remarks to the Author)
This manuscript demonstrates a concept of multiphase-controlled product scavenging (MCPS), which combines the scavenger and LLPS concepts together. The help of a scavenger strand increases the catalytic turnover, and is further enhanced by co-localizing ribozymes and transcription templates within DNA nanostar-based LLPS condensates.

The work of using scavengers to improve the catalytic turnover of ribozyme/DNAzyme is not new, just as the authors cite the work of Winfree in 2009. LLPS mediated by nanostars is also based on classic work from the Franco and Schulman labs. In this manuscript, the authors combine these two well-known concepts together to achieve a programmable framework to manipulate the ribozyme catalytic capacity. I think the concept is quite intriguing. Although the two concepts are not new, coupling them together leads to a novel idea to engineer ribozyme efficiency. That's why I think this work is noteworthy for publication in Nature Communications. However, I would recommend the authors to improve the structure of the manuscript to present this concept in a more rigorous way before the manuscript is accepted.

Major issues:

1. Generalizability of scavenger designs

In the manuscript, the design principle of generalizable scavengers was not elaborated clearly. The authors mention that they used NUPACK to help with the sequence design, but the criteria or design rules used to achieve efficient scavengers were not clearly described. For readers who would like to cite this manuscript and design scavengers for other types of ribozyme or DNAzyme, what rules should they follow? The figures and main text do not mention this clearly. It is somewhat difficult to infer the base-pairing regions directly from Figures 3C and 3D.

2. Generalizability of MCPS

In Figures 2 and 3, the authors provide examples of scavengers for the hammerhead ribozyme, the 10–23 DNAzyme, and the hairpin ribozyme, demonstrating that the scavenger strategy can function across different types of nucleic acid enzymes. However, for the central concept of this work,MCPS, the manuscript currently presents only one example using the hammerhead ribozyme.
Figure 4c suggests two possible strategies to generate LLPS, either by co-partitioning the nanostar with HHR or with the DNA template of the RNA substrate. However, it appears that only one of these approaches was used in Figure 5C. This point would benefit from clarification. It would be helpful to know whether the authors also tested the alternative strategy and, if so, what the outcome was.
While the concept of a "generalizable and programmable framework, multiphase-controlled product scavenging (MCPS)" is appealing, further clarification or additional examples would help readers better appreciate the scope and generalizability of this approach.

3. Some important details for Fig 5c need clarification

The most exciting experiment, in my opinion, is Figure 5c. However, I did not clearly understand how the ligation reactions were quantified and how the authors arrived at their conclusion. Maybe the authors have described this somewhere but I did not catch it.
(1) I'm convinced that DNA template of RNA substrate co-partition with LLPS of nanostars. However, each DNA templates can generate multiple copies of RNA substrates. Is there any way to show that all RNA substrates co-parition in the condensate? Though ref 49 is beautiful, Figure S19 is yet not obvious evidence. It is unclear that all of RNA copies co-partitioned in the droplets. Any the fraction of RNA that diffuse freely in and out the dilute phase? If so, how can we confidently quantify the yield of RNA cleavage reactions?
(2) Please provide reference or experiment to validate the efficiency of ligation reaction in step 3 of Figure S20, since this step is the key readout. If 2',3' cyclic phosphate is hydrolyzed to form either 2' or 3' mono phosphate, will the reaction still happen? (I think it is unlikely.) The hydrolysis of 2',3' cyclic phosphate might happen quite fast during heat treatment in step 2 of Figure S20.
(3) The increase in reaction is presented in Figure 5C, which appears better than single-phase product scavenging. However, this figure only presents the increase in fraction of downstream ligator reacted, but not the turnover or reaction rate. The relevant numbers are hard to find in figure legend or main text regarding the kinetic shift and the concentration of scavenger.
(4) Importantly, please include the raw gels of figures 5B and 5C in the SI. This will convince the readers of the conclusions.

4. Structure of the manuscript

The author spent a large portion of manuscript establishing of scavenger concept, including the modeling and kinetic analysis in figure 2 and 3. While readers may appreciate this is as a foundation before moving towards MCPS, this concept itself is not new. I'm not quite sure whether this part helps the readers better appreciate this work or instead dilute the importance of the key point of this manuscript. In my understanding, the novelty and excitement of this manuscript lie mainly in figure 5.
With all above three major issues, I would recommend to adjust the manuscript to include more data and discussion showcasing the programmability and generalizability of MCPS.

Minor issue:

1. With the scavenger, the mechanism of the dynamic circuit is different from equilibrium-shifting reaction? If the scavenger-mediated displacement changes microscopic rate constants or merely shifts effective equilibria? Could you make it clearer?
2. The kinetic modeling contains some assumed or fitted parameters, such as the substrate transfer from scavenger to ribozyme-product complex. Could you provide some experiments to explicitly report parameter ranges tested? Or confidence intervals for fitted rates?
3. This paper discusses a lot about the tradeoff between substrate scavenging and product scavenging. Do you have any direct quantitative measurements of substrate-scavenger binding affinity?
4. Fig.1 could much clearer labeling of rate-limiting steps.
5. Fig. 3A needs the annotation for lines. Fig. 3B is hard to see all conditions mentioned in the annotation.
6. Fig.5 should show the conditions or annotations in the graphs.
7. Some data not specified with numbers of replicates. Please clarify n values consistently.
8. What's the concentration of scavenger to the HHR and HHR sub? The manuscript does not always mention. It would be helpful to include this information in the figure captions. (For instance, I was wondering about this when I read figure 2, but I had to dig into the SI and found the scavenger concetration is 20 uM, if I understood correctly.)

(Remarks on code availability)

Reviewer #4

(Remarks to the Author)
I co-reviewed this manuscript with one of the reviewers who provided the listed reports. This is part of the Nature Communications initiative to facilitate training in peer review and to provide appropriate recognition for Early Career Researchers who co-review manuscripts.

(Remarks on code availability)

Version 1:

Reviewer comments:

Reviewer #1

(Remarks to the Author)
The authors have substantially revised and improved the manuscript and I think it is well on the way to acceptance. A couple of issues remain incompletely addressed, however

1. The figures are still not publication quality; the text and lines that should be very sharp are clearly blurred when one zooms in. I leave this as something to be addressed when the proofs are prepared.

2. The model on lines 92-99 of the SI still wouldn't behave in a biphasic way, I think. You can still just add the pathways together in the ODEs (this time yielding a single process with a weighted average rate). To get a biphasic behaviour, one would need to explicitly represent the two variants of the ribozyme (in all its complexes) as separate variables -- eg. [ScR1] and [ScR2].

3. The authors have indeed now cited work on templated ligation/assembly (not both the ones that I recommended, as I understood from the rebuttal, but one of those and some alternatives). But the key point is that they haven't fully engaged with the differences from their work, which is a shame.

(i) In line 89-93, where the citation is made, the authors aren't really clear enough that they are drawing a contrast with ligation/assembly and their work. Perhaps simply extending the "severe" to "even more severe than for cleavage reactions" is enough to make this point.

(ii) The authors really should engage with the Ref 28 properly in the Discussion with regard to its demonstration of a scavenger-like motif. There are relative strengths and weaknesses of both papers, and understanding them is very helpful in thinking about where this work could go next. In particular, a major weakness of the manuscript under consideration is that scavengers bind to substrates - an advantage of Ref. 28 is that the equivalent to the scavenger only binds to products, because the assembled product presents a very different target from the reactants. How to generalize such a strategy to cleavage is not immediately obvious, but exploring whether it is possible is surely of interest and worth discussing?

(Remarks on code availability)

Reviewer #2

(Remarks to the Author)
I am satisfied with the authors' response and the revisions made to the manuscript. Therefore, I recommend it be accepted for publication.

(Remarks on code availability)

Reviewer #3

(Remarks to the Author)
I appreciate the considerable effort the authors have made in revising the manuscript. The revised version is much improved. The authors have addressed all major and minor concerns in my previous review. The additional data and analyses have strengthened the manuscript. The figures are also clearer. I continue to find the central concept interesting and believe this work represents a useful conceptual advance to mitigate product inhibition in nucleic acid catalysis. I would love to see this work published soon.

I only have two minor comments, which may simply reflect my personal writing preference.

1. Although the manuscript is clearer than before, I still found parts of it more difficult to follow than necessary, and some sections could be a bit wordy. In several places, lengthy conceptual ideas are introduced before the experiment is clearly described, making the flow less straightforward. I think the manuscript would benefit from a more streamlined presentation.

A very minor point: I would also avoid repeating the same transition words in adjacent sentences. For example, line 239-241, both sentences begin with "Thus…" ; line 400-402, both sentences begin with "Because…."

2. I encourage the authors to be slightly more conservative when discussing the generality of the approach. The revised manuscript now demonstrates the concept across three different nucleic acid enzymes, which strengthens the work. However, it remains unclear whether the same strategy can be broadly applied to other catalytic systems.

(Remarks on code availability)

Reviewer #4

(Remarks to the Author)
I co-reviewed this manuscript with one of the reviewers who provided the listed reports. This is part of the Nature Communications initiative to facilitate training in peer review and to provide appropriate recognition for Early Career Researchers who co-review manuscripts.

(Remarks on code availability)

Version 2:

Reviewer comments:

Reviewer #1

(Remarks to the Author)
I thank the authors for responding to my comments. I am essentially happy to proceed to publication.

The model/fitting is still a little confusing, and I would encourage the authors to further tidy it up prior to publication. It is not that clear that the biphasic fit referenced around line 85 follows from a model with fundamentally different assumptions from the kinetic model used subsequently, perhaps that could be clarified.

I would also advise against leaving the ODEs with two cleavage terms. It is encouraging misinterpretation. Surely it would be simpler to present a single term in the ODE, and say that its rate was taken as a weighted average of the previous fit?

(Remarks on code availability)

Open Access This Peer Review File is licensed under a Creative Commons Attribution 4.0 International License, which permits use, sharing, adaptation, distribution and reproduction in any medium or format, as long as you give appropriate credit to the original author(s) and the source, provide a link to the Creative Commons license, and indicate if changes were made.
In cases where reviewers are anonymous, credit should be given to 'Anonymous Referee' and the source.
The images or other third party material in this Peer Review File are included in the article's Creative Commons license, unless indicated otherwise in a credit line to the material. If material is not included in the article's Creative Commons license and your intended use is not permitted by statutory regulation or exceeds the permitted use, you will need to obtain permission directly from the copyright holder.
To view a copy of this license, visit https://creativecommons.org/licenses/by/4.0/

Point-by-Point Response

Manuscript #: NCOMMS-26-007007-T

Title: Dramatic reaction enhancement via product scavenging and phase separation-driven pathway canalization

Corresponding Author: Dr. Rebecca Schulman

Authors: Byunghwa Kang *et al*.

Reviewer #1

Reviewer overall comment:

Kang et al present a mechanism for enhancing the rate with which a nucleic acid catalyst turns over its nucleic acid substrates. The system consists of a RNAzyme that cleaves a specific target RNA strand, but whose turnover is limited by the fact that both halves of the target remain bound to the DNAzyme after cleavage. The authors introduce a scavenging strand that displaces one of the products, allowing enhanced turnover. However, the scavenger also tends to bind to the uncleaved substrate, slowing down the initial binding. On balance, the effect still accelerates turnover, and this effect is enhanced when catalysts are co-localised with the production of the RNA strands in a phase-separated system. The authors also show that their approach works for two other RNA-cleaving RNAzymes.

Overall, the principle is widely interesting and novel (although note comment 1a below). The results are clear. There are a number of issues with the way the results are presented and discussed, outlined below, that should be fixable. A higher-level criticism relates to design choices in the system; it isn't clear to me that these catalysts *need* to operate in product inhibited context, and it's not obvious that the designed scavengers are particularly well designed, given the catalyst and product combination, to circumvent inhibition. These factors don't invalidate the results, which do show the effect claimed, but it would be good to explore these factors more thoroughly.

Overall response: We sincerely appreciate the reviewer for the thoughtful and constructive assessment of our manuscript, and for recognizing the novelty and general interest of the underlying principle. We appreciate the reviewer's concerns regarding the presentation, mechanistic discussion, and design choices of the scavenger systems. We address each concern raised by the reviewer below in a point-by-point manner. We believe these revisions have significantly strengthened the manuscript.

Major comments:

1. Catalyst design. The authors are accelerating turnover in a cleavage reaction. Cleavage is actually the type of catalysis that is most immune to product inhibition, since the separated products will no longer cooperatively bind to the template -- they will detach *far* faster than the substrate. In order to experience product inhibition, therefore, both halves of the product need to be quite long (10/11 nt here).

(a) In line 87, the authors say that product inhibition is common in vivo because "substrates and products often differ by only a single cleavage-ligation event". But cleavage and ligation are actually massive changes! Cleaving a 21mer into a 10mer and an 11mer turns detachment from something that will never happen on feasible timescales to something that takes ~10 hours, according to the results. Slightly (a couple of base pairs) shorter arms would lead to quick detachment of the products, while the substrate would still stick extremely strongly. By contrast, if the catalyst is acting to template ligation, product inhibition becomes an unavoidable and enormous challenge, because the products tend to stick to the catalysts more strongly than the substrates due to cooperativity. The authors should explain these concepts more carefully in the introduction/discussion. They might note that recent work has explored how product inhibition can be overcome in DNA templating of (non-covalent) association [https://www.nature.com/articles/s41557-025-01831-x], and even introduced a mechanism based on a scavenger-like strand to accelerate it, although here the scavenger only binds to the product after it is formed [https://www.biorxiv.org/content/10.64898/2026.02.18.706517v1.abstract - this latest work was published online after the paper under consideration was submitted, and so does not compromise its novelty].

Response: We thank the reviewer for this helpful comment. We agree that our original wording did not sufficiently distinguish cleavage from ligation or templated assembly. Our intention was not to suggest that cleavage is only a minor perturbation, but rather that cleavage products can remain inhibitory when they retain substantial complementarity to the catalyst. We have revised the Introduction accordingly to clarify that, in cleavage systems, the extent of product inhibition depends on the residual product-catalyst duplex stability, whereas in templated ligation or assembly it can be even stronger because the joined product often binds cooperatively more strongly than the separate reactants. We also now cite the recent work noted by the reviewer.

Line 88-93 in the revised manuscript

(b) As the authors note on line 167, shorter recognition domains can be used, which would mitigate product inhibition in cleavage. They say that this speedup comes at the cost of specificity and affinity, but I am not sure about this argument. On the thermodynamics of base pairing alone (ignoring whether reduction in recognition domain length compromises catalytic activity once bound), there is surely a fairly large window in which the substrate, which binds cooperatively via two arms, will have strong affinity for the catalyst but the products, which do not bind cooperatively, will not. The authors cite the fact that the HDV ribozyme requires 15-16nt of recognition - but this is quite a lot less than the 21 used here, enough to (probably) eliminate product inhibition as a significant effect. The claim about specificity is also not clear to me. A longer recognition domain will be more tolerant of a mismatched base pair, not less, according to the nearest neighbour model. At the very least, more discussion of why particular recognition domain lengths were chosen is required

Response: We thank the reviewer for this thoughtful comment. We agree that our original wording on specificity was too imprecise. Our intention was not to claim that longer recognition domains directly enhances single-mismatch discrimination. Rather, our point was that, in practical RNA-targeting applications, productive processing of long cellular RNA targets often depends on target accessibility and formation of a catalytically competent folding, not just idealized duplex thermodynamics for a short unstructured substrate (*Nucleic Acids Res.* 25, 769-775 (1997), *Nucleic Acids Res.* 25, 4985-4993 (1997), *Nucleic Acids Res.* 52, 11177-11187 (2024). This is especially

relevant for ribozyme and DNAzyme applications against long structured RNAs, where accessibility of the target site and availability of the recognition arms are often dominant design constraints. For instance, prior work on hammerhead ribozymes showed that cleavage efficiency for long RNA substrates depends strongly on local target structure and accessibility, and that extending antisense arms can facilitate cleavage of longer RNA targets by engaging accessible flanking domains. Therapeutic antisense oligonucleotides likewise commonly operate in the ~15–30 nt range, illustrating that practical targeting often uses substantially longer recognition domains than the minimum needed for binding in an idealized short-substrate model. We have therefore revised the manuscript to distinguish mismatch discrimination from target accessibility and productive target binding, and to clarify that the 21-bp recognition system was chosen as a deliberately stringent, product-inhibited model relevant to structured-targeting regimes, rather than because longer arms are inherently more sequence-specific.

Line 172-180 in the revised manuscript

2. Scavenger design. The authors design the default scavenger to do a form of toehold exchange, leaving a 2 base-pair "output" toehold for the catalyst with the product fragment that is removed. This isn't discussed much in the text and the diagrams don't usually show these free bases. But this toehold is potentially very important, if the rate limiting step is really the reaction between catalyst bound to half the product and a "scavenged" substrate, as suggested by Fig. 1b. One would surely expect this reaction to proceed via this toehold? The reaction rate observed is on the right order of magnitude.

The authors don't discuss this rate limiting step in detail, but they do give some alternative designs in the SI. However, these designs only consider shortened versions of the output toehold (and shortened versions of the input toehold). Surprisingly to me, the strands with a shortened output toehold are not that much worse at accelerating the reaction, although they are a bit worse. However, what I really don't understand is why the authors didn't try an increased (3,4 base) output toehold. It might make the scavenging a bit slower, but the rate limiting step is binding of the scavenged substrate, so this is surely a price worth paying?

At the very least, the authors should discuss the design of the scavenger probes in a bit more detail, focussing on the question of toehold exchange. Why was the design selected? Why were longer output toeholds not tested? In their position I would probably test the efficacy of a longer output toehold (Sca-15' and Sca-14').

Response: We thank the reviewer for this insightful comment. We agree that the strand-exchange geometry, including the use of the residual "output" toehold left on the ribozyme-bound product, could be an important design feature. And, as the reviewer points out, such a design could mitigate substrate-scavenger inhibition, speeding up the reaction further.

The reason why we selected Sca-16 as the default design in the present study, despite these benefits, is that it empirically provided the best overall balance between efficient product displacement and retention of productive HHR:HHR Sub complex formation. The output toehold domain lies very close to the ribozyme catalytic core, so we hypothesized that modifying this segment by extending would be unlikely to act as a simple, independently tunable toehold and instead could affect molecular accessibility, catalytic-core folding, and product scavenging.

That said, we did not test this hypothesis. Therefore, in response, in addition to Sca-16, we tested six more related scavenger variants with altered input and output toehold lengths and have included these results in the revised version. All of these variants enhanced HHR cleavage rate relative to the no-scavenger control, and importantly, among equal-length pairs, as reviewer expected, Sca-15-3' (5.4 turnovers for 2 h) and Sca-14-3' (4.4 turnovers in 2h) outperformed Sca-15 (4.4 turnovers in 2 h) and Sca14 (4.1 turnovers in 2 h), respectively. This is presumably consistent with a beneficial effect of a longer toehold during the reaction of the scavenged substrate with the ribozyme.

However, at the same time, the improvement was modest rather than dramatic. Because toehold extension can, in principle, produce much larger rate enhancements (10-100 fold) in canonical strand-displacement reactions, this result suggests that scavenger performance in the present system is governed by multiple coupled factors rather than by output-toehold length alone. Consistent with this interpretation, Sca-18 (3.8 turnovers for 2 h), which provides a 0-nt output toehold, and Sca-17 (3.9 turnovers for 2 h), which provides a 1-nt output toehold, showed very

similar catalytic enhancement than Sca-16 (4.5 turnovers for 2 h), which provides a 2-nt output toehold.

We therefore conclude that, in this ribozyme-scavenger system, scavenger behavior cannot yet be predictably programmed simply by changing the output-toehold length. We have revised Figure S6 and its caption to include new experimental data and have expanded the discussion of scavenger design and toehold exchange accordingly.

<u>Line 171-178 in the revised SI</u>

<u>Line 210-219 in the revised SI</u>

3. Theoretical modelling. It is good that the experimental results are supported by theoretical modelling, but aspects of it are very unclear.

(a) How were the two cleavage rates handled in the analysis? In Supplementary note 1, the two cleavage rates just appear together in the equation, which will be equivalent to a single process with a rate equal to the sum of the two - this will not yield the observed two-phase behaviour. The same applies to the two ligation rates used.

Response: We thank the reviewer for this careful comment. The reviewer is mathematically correct, as written in the previous Supplementary Note 1, the equation is mathematically equivalent to a single effective process and does not explicitly show biphasic behavior. The original equations were written incorrectly, *i.e.* they did not accurately reflect the model used in the analysis. In the actual fitting/simulation, we used two non-interconverting ribozyme subpopulations (fast and slow; R_f and 1-R_f, respectively) with the initial fractions in each subpopulation taken from the single-turnover kinetic analysis. We have now corrected Supplementary Note 1, Supplementary Note 3, and Table S4 so that the equations accurately reflect the model used in the analysis, which shows biphasic behavior.

<u>Line 94-97, 102-103, 237-241, and 325 in the revised SI</u>

(b) It is also unclear why the replacement of the two products with the substrates is modelled as a single transition with a rate depending on [S] and [ScR]. I can see how something like this might emerge from a more detailed description, but it isn't really justified.

Response: We thank the reviewer for this insightful comment. We agree that this step is a coarse-grained approximation, not a literal elementary reaction. In reality, replacement of the products by the substrate is expected to involve multiple sequential steps, including dissociation of either 3' or 5' product and substrate-mediated displacement of the remaining product to re-form the substrate-ribozyme complex. To simplify the model by assuming there would be a rate-limiting step in this complex process, we represented these unresolved steps as a single effective transition relying on [S] and [ScR]. This simplification avoids overparameterization and the creation of reactions whose rates could not be measured directly. We have revised Supplementary Note 1 to clarify that this term is a phenomenological lumped rate constant rather than a mechanistically resolved single step. Importantly, this simplified treatment was sufficient to capture the turnover kinetics across all three nucleic acid enzymes (hammerhead ribozyme, hairpin ribozyme, and 10-23 DNAzyme).

<u>Line 110-116 in the revised SI</u>

4. The thermodynamic analysis (Figure S5 and Table S2) in the SI is very confusing. First, the Sugimoto parameters can be used to predict the stability of DNA/RNA duplexes. It is not always true that DNA:RNA is more stable than DNA:DNA, it depends on the balance of purines in the RNA strand.

Response: We thank the reviewer for this insightful comment. We agree that our original statement was too general (Line 136 in SI). The thermodynamic stability of DNA:RNA duplexes is sequence-dependent, and it is not always valid to state that DNA:RNA is more stable than DNA:DNA. In the revised SI, we removed a general statement to this effect and clarified that our NUPACK analysis used DNA-only and RNA-only systems as a qualitative proxy. We also checked RNA/DNA hybrid nearest-neighbor parameters reported by the Sugimoto group (doi: 0.1093/nar/gkaa572) for the scavenging reactions and found that these parameters predict that the DNA:RNA duplex is more stable than DNA:DNA duplex. Thus, the proxy and this analysis agree,

preserving the qualitative conclusion of the thermodynamic analysis. We now note this point in the Figure S5 caption and cite the corresponding reference.

Line 185-188 in the revised SI

Second, the caption of Table S2 is very hard to read and understand. Finally, I am confused as to why the percentage of HHR bound to HHRsub increases as the amount of Sca16 increases in the top table.

Response: Thank you for this careful comment. We agree that the original caption of Table S2 was unclear. We have revised the table and caption to state explicitly that these values are NUPACK-predicted equilibrium fractions of HHR Sub bound to HHR. We also list the strand compositions/concentrations more clearly and have corrected the values in the top table.

Line 196-204 in the revised SI

Minor Comments:

5. Is the term "enzyme" typically used for RNAzymes?

Response: We thank the reviewer for the comment. To avoid any possible ambiguity, we used the terms “nucleic acid enzymes”, “protein enzymes,” and “RNA enzymes” where appropriate in the manuscript.

6. Figure quality is sometimes not great. The images have artefacts from conversion to PDF, and the nucleic acid strands are shown with kinks in them even when they are part of duplexes, which is strange.

Response: We thank the reviewer for this careful comment. We have revised Figure 1, Figure 2B, Figure 2H, Figure 3A-B, and Figure 4D in the manuscript to ensure that the images are clear and

do not have conversion artifacts. We have also removed kinks from diagrams of duplexes in the main text and SI.

7. Line 201 - does "predicted" mean "estimated based on fits"?

Response: We thank the reviewer for the comment. ‘A predicted turnover frequency’ has been revised to “An estimated turnover frequency” in Line 201.

<u>Line 208-210 in the revised manuscript</u>

8. Lines 235-236 need rephrasing for clarity.

Response: We thank the reviewer for this comment. We have rephrased lines 235-236 to clarify the rationale for scavenger design.

<u>Lines 241-243 in the revised manuscript</u>

9. I think it would be very beneficial to come up with an abbreviated, consistently-used nomenclature for the species in the system, it's clunky to keep saying eg. "3' end product" and complexes described in this way are particularly awkward.

Response: We thank the reviewer for this helpful suggestion. We already used HHR and HHR Sub to denote the hammerhead ribozyme and substrate, respectively. Following the reviewer’s comment, we have further simplified the nomenclature by abbreviating the 3’ and 5’ end products as 3’P and 5’P, respectively. Similarly, the ribozyme-5’ end product complex is now referred to as HHR-5’P. These abbreviations are now used consistently throughout the revised manuscript and SI.

10. The definitions of the parameters and reactions in the models are very hard to follow from the text. Is it possible to illustrate them in the main text itself?

Response: We thank the reviewer for this constructive comment. We agree that the full set of modeled reactions and parameter definitions was difficult to follow from the original text. To improve clarity, we have revised the manuscript in three ways.

First, we added terms representing the rates of key scavenger-mediated reactions directly to Figure 2E–G, so that the most important kinetic processes are illustrated in the main text alongside the relevant model outputs. Second, we substantially revised the main-text paragraph describing the kinetic model involving scavengers. In the revised text, we now focus on the two critical reactions that primarily determine the rate of HHR cleavage: scavenger-mediated release of 3′P, described by k_{sca}, and substrate transfer from the scavenger to the ribozyme pre-hybridized with 5′P, described by k_{trf}. The other hybridization steps were assigned association rate constants of 1×10^6 $M^{-1}sec^{-1}$, a commonly used baseline value for short, unstructured DNA or RNA oligomers in solution. Because varying these additional hybridization rates had negligible effects on HHR turnover, as shown in Figure S8, we shortened their discussion in the main text to reduce complexity and improve readability.

Third, we expanded Supplementary Note 3 by adding Figure S9, which summarizes the expected reactions and kinetic parameters used in the reaction model. This new supplementary figure focuses on the fate of the ribozyme and its complexes with substrate or products, thereby providing a comprehensive overview of the modeled reaction network. We considered adding this full reaction scheme to the main text. However, when we made the full diagram, it was clear that including all reactions and rate constants would make the main text figure overly complex. We therefore chose to keep the main text focused on the central MCPS concept while providing the complete reaction schematic in the Supplementary Information.

<u>Line 269-272 in the revised SI</u>

<u>Line 279-287 in the revised manuscript</u>

11. I couldn't follow lines 271-272 - the substrate isn;t transferred fro the scavenger to the "complex", surely? It disrupts the complex.

Response: We thank the reviewer for this comment. We agree that the original wording was unclear. We have revised lines 271-272 to clarify that the substrate is transferred from the scavenger-bound state to the ribozyme while disrupting the HHR-5'P complex, rather than being transferred to the intact complex.

<u>Line 291-293 in the revised manuscript</u>

12. Why is cleavage/ligation site used in Fig3d but not in the others?

Response: Thank you for pointing out this. We have revised Figure 3C in the manuscript to include this pointer. Figures 2B and 3D already denoted the cleavage/ligation site.

13. 322-323 The ratio of ligation/cleavage rates is determined by the thermodynamics of the reactants and the products, not the catalyst.

Response: We completely agree with the reviewer that the global thermodynamic equilibrium constant (K_{eq}) between free reactants and free products is an inherent property of the RNA sequences themselves and cannot be altered by a catalyst; a catalyst, by definition, simply can speed up progress toward that equilibrium by changing the activation energy. Our original wording was ambiguous. We appreciate the reviewer's comment and have clarified this in the revised manuscript. To recap the specific concern here, the cleavage and ligation rate constants ($k_{cleavage}$ and $k_{ligation}$) in our kinetic model describe the internal catalytic rates of the enzyme-bound states (ES ⇌ EP). According to the thermodynamic cycle of enzyme catalysis, the internal equilibrium constant ($K_{int}= k_{cleavage}/k_{ligation}$) (but not the overall conversion between substrate and product) depends on how strongly the catalyst stabilizes the substrate versus the product. Because the 10-23 DNAzyme and the four-way junction hairpin ribozyme (HairR) are structurally distinct catalysts targeting different sequences, their relative stabilization of the ES and EP states can differ substantially. This explains why their internal ligation/cleavage rate ratios are inherently system-dependent and differ.

Our study introduces a scavenger to continuously sequester the cleavage products. The scavenger, unlike a catalyst, is a stoichiometric agent that supplies an additional reactant (scavenger) to

remove free products from the reaction network, the scavenger suppresses rebinding of products and thereby reduces the effective reverse reaction, allowing ribozymes with high intrinsic ligation tendencies, such as HairR, to sustain multiple-turnover cleavage. Because the scavenger is consumed in a reaction with a product, it can alter the equilibrium between substrate and product. We have clarified this distinction in the revised Table S4.

Line 327-330 in the revised SI

Line 352-354 in the revised manuscript

14. In Fig 4D the text is low quality.

Response: We thank the reviewer for the comment. We have revised Figure 4D.

15. Line 356-357 is confusing. It sounds like 86.4 is the measured frequency, but I think you mean this is the maximum possible?

Response: We thank the reviewer for this constructive comment. We agree that the original sentence was ambiguous. We have revised the text to clarify that 86.4 h^{-1} is a model-predicted upper limit in the absence of substrate scavenging, not the experimentally measured turnover frequency.

Line 387-389 in the revised manuscript

16. Line 373 - what precisely is meant by LLPS droplets "mix rapidly"?

Response: We thank the reviewer for this comment. By “mix rapidly,” we meant that molecules in the LLPS system undergo dynamic exchange between the dense and dilute phases. We have revised the text to state this more precisely.

Line 400-402 in the revised manuscript

17. Line 415 - the evidence presented is that the scavenger doesn't act as a surfactant - is this the same as saying it doesn't interact with the droplets?

Response: We thank the reviewer for this interesting question. The absence of a surfactant-like effect of the scavenger (in Figure 4D) is not, by itself, equivalent to saying that the surfactant has no interaction with the droplets. Our interpretation that the scavenger exhibits no measurable interaction with the DNA droplets is based on the combination of two observations: first, increasing Sca-16 concentration had negligible effects on droplet size (Figure 4D), indicating that Sca-16 does not measurably perturb droplet morphology (no effective change in interfacial properties of the droplets) which would affect multivalent interactions between DNA nanostar oligomers; second, the partition coefficient of FAM-labeled Sca-16 was 1.03 ± 0.02 (Figure 4E), indicating no preferential enrichment of the scavenger in the dense phase. Together, these data support the conclusion that Sca-16 does not appreciably associate with DNA droplets under our experimental conditions.

18. Line 507 - is the multiphase acceleration dramatic? It's a factor of 2.

Response: We thank the reviewer for this comment. In the manuscript, "dramatic" was intended to describe the combined enhancement produced by scavenging together with the multiphase system, rather than the effect of the multiphase system alone.

We have revised the manuscript to clarify this point. However, in response to Reviewer 3's concern about presenting only the increase in fraction of downstream ligator reacted rather than the turnover or reaction rate (in Figure 5), we also created a calibration method that allows us to more precisely quantify the extent of enhancement due to the multiphase organization of the reaction (newly added Supplementary note 5 with Figure S26). We find that the multiphase system increased the substrate cleavage rate mediated by HHR by 6.5-fold over a single-phase product scavenging system, which, given that the single-phase product scavenging increases conversion 40-fold, is a >250-fold enhancement, which we consider dramatic, and we hope the reviewer concurs!

Line 471-511 in the revised SI

Line 547-556 in the revised manuscript

19. Line 635 - was it tested whether this complementary strand inhibited the HHR? I agree it should.

Response: We thank the reviewer for this question. We tested whether the complementary strand inhibits HHR activity by comparing two samples under the same buffer and heat-treatment conditions used in the manuscript. In the first sample, we included the FAM-labeled HHR Sub alone as no ribozyme activity control. In the second sample, we included 1X HHR and 1.1X complementary strand to HHR, followed by the addition of the FAM-labeled HHR Sub and immediate heat treatment according to our methods. In both samples, we observed no detectable length change of the FAM-labeled HHR Sub in resulting gel electrophoresis. The sample containing HHR plus the complementary strand was indistinguishable from the no-ribozyme control, indicating that the complementary strand completely inhibits HHR cleavage activity under these conditions. We have added this experimental result to the revised SI (Figure S21C).

Line 418-423 in the revised SI

20. Line 95 of SI - I think different notation is being used from what is used in the equations themselves.

Response: We thank the reviewer for pointing this out. We agree that the notation was inconsistent. We have revised Supplementary Notes 1 and 3.

Line 85 and 223-224 in the revised SI

21. The caption of Figure S3/S9/S13 could be a bit more explicit about what it is describing. Simulation of what?

Response: We thank the reviewer for this helpful comment. We agree that the original captions of Figure S3, S9 (now S10), and S13 (now S14) did not clearly state what was being simulated. We have therefore revised these captions to specify that the plots show simulated catalytic turnover

over time predicted by the kinetic models, together with the linear regression used to extract turnover frequency.

Line 120-123, 276-278, and 339-344 in the revised SI

Reviewer #2

Reviewer overall comment:

The authors present an exciting strategy to overcome product inhibition, a fundamental bottleneck in catalysis where slow product dissociation limits turnover. By using nucleic acid enzymes as models, they demonstrate that a designed affinity reagent—a scavenger—can actively displace products via toehold-mediated strand exchange. This "product scavenging" increases turnover up to 39-fold. To address the inevitable "substrate scavenging" (where the scavenger binds to the structurally similar substrate), the authors utilise DNA nanostar-based condensates to co-localize enzymes and substrates to achieve catalytic rate enhancement of approximately 70-fold. This work is an excellent demonstration of using predictable base-pairing rules to engineer novel biochemical pathways. The integration of thermodynamic displacement (scavengers) with spatial control (LLPS) is a modern approach to further control such reaction pathways. However, several points regarding the energy requirements, the scope of generalisation, and the clarity of the proposed mechanisms require further clarification. Therefore, I would like to ask for reconsideration of the paper after the following points have been addressed.

Overall response: We thank the reviewer for the highly positive and thoughtful evaluation of our work, and for their explanation of both the novelty of the product-scavenging concept and the broader significance of integrating thermodynamic displacement with spatial control in LLPS-driven systems. We also appreciate the reviewer's thoughtful comments and questions regarding the energetic cost of scavenging, the scope of generalization, and the clarity of the mechanistic presentation, and appreciate the opportunity to improve the manuscript to address them. We have revised the manuscript and SI to clarify these points, refine the discussion of the system's scope and limitations, and improve the presentation of the proposed reaction pathways and figures as described in the following point-by-point responses.

Major comments:

The "Energy Cost" of Scavenging: The introduction of a scavenger to "clear" the product requires the addition of a high-energy species (the free scavenger strand) which ends up in a lower-energy

state (the scavenger-product duplex). While the catalytic rate is enhanced, the overall system is "driven" by the chemical potential of the scavenger. Could the authors provide a more detailed discussion on the efficiency/trade-off for this system? Specifically, how does the "cost" of providing the scavenger compare to the benefit of accelerated turnover in a potential therapeutic or diagnostic setting?

Response: We thank the reviewer for this insightful comment. We agree that scavenger-assisted product removal is not energetically free. In our system, the enhancement in catalytic flux is driven by the favorable binding free energy released when the free scavenger forms a more stable scavenger-product duplex. Thus, the scavenger acts as a stoichiometric affinity reagent whose available binding capacity is consumed as product accumulates, rather than as a catalyst. Thus, MCPS improves the kinetics of catalyst turnover by relieving product inhibition, but this comes at the cost of consuming scavenger binding capacity as product accumulates.

We have now clarified this tradeoff in the revised manuscript (Discussion section). Specifically, the benefit of scavenging is not improved thermodynamic efficiency of the overall system but improved kinetic efficiency of catalyst usage: scavenger binding removes a major kinetic bottleneck, namely slow product dissociation, and thereby increases catalyst turnover. This comes at the cost of requiring free scavenger in excess, which is also consistent with our experimental and modeling results showing that scavenger concentrations must exceed substrate concentration to produce strong rate enhancement. At lower scavenger concentrations, the scavenger is largely tied up by substrate and does not efficiently accelerate product release.

Importantly, this trade-off can be favorable in practice because the additional driving reagent is a DNA oligonucleotide, whereas the catalyst is any nucleic acid enzyme, including ribozymes. DNA strands are generally less expensive to synthesize, easier to handle, and more stable during storage than RNA enzymes. Thus, MCPS provides a practical strategy for accelerating a process catalyzed by an RNA enzyme using a comparatively inexpensive and stable DNA affinity reagent. In this context, the energetic cost of scavenging is offset by improved use of the RNA catalyst: a stoichiometric DNA reagent is consumed to relieve product inhibition and enable faster ribozyme turnover. This distinction is particularly relevant for diagnostic or analytical applications, where rapid signal generation and efficient use of the catalytic RNA component are often critical. Rather

than requiring additional ribozymes to compensate for slow turnover, MCPS uses DNA scavengers to increase the productive turnover of existing ribozyme molecules. We have revised the Discussion to clarify that the multiphase architecture does not eliminate the energetic cost of scavenging but improves the utilization of this driving force by biasing substrate toward productive catalyst binding and away from unproductive scavenger association.

Line 601-620 in the revised manuscript

Evaluation of the "Generalizability" Claim: The manuscript claims MCPS is a "generalizable and programmable framework". While this is clearly shown for three distinct nucleic acid enzymes, the jump to protein-based enzymes mentioned in the Discussion is non-trivial. While base-pairing rules (NUPACK) allow for precise scavenger design, for protein enzymes, finding affinity reagents (antibodies/aptamers) that bind products with higher affinity than the enzyme—without blocking the active site during the reaction—is significantly harder.

Response: We thank the reviewer for this important comment. We agree that the generalizability demonstrated in the present study is strongest for nucleic acid enzymes, for which scavenger design can be guided by predictable base-pairing interactions and thermodynamic analysis. We also agree that extending this strategy to protein enzymes would be substantially more challenging, because suitable product-selective affinity reagents would need to be identified and optimized without perturbing the catalytic process.

In response, we have revised the Discussion to clarify this distinction and to avoid overgeneralizing beyond the systems experimentally demonstrated here. Specifically, we now emphasize that nucleic acid enzymes themselves constitute a broad and important class of catalysts, including ribozymes, DNAzymes, and emerging CRISPR-associated systems, for which programmable product scavenging may be broadly applicable.

We further clarify that the broader conceptual principle demonstrated here is not necessarily nucleic-acid-specific: namely, that a secondary cofactor or affinity reagent can lower the effective energetic barrier for product release and thereby enhance turnover in systems limited by product inhibition or slow product dissociation. While implementing this principle for protein enzymes

would require substantially different molecular strategies and extensive empirical optimization, we believe the concept may motivate future investigation in systems where product-selective affinity reagents are already available or could be engineered.

To make this point more concrete without overstating feasibility, we now discuss several possible future directions only as illustrative examples. These include SAM-dependent methyltransferases, where SAH-binding aptamers have been reported, and UDP-producing glycosyltransferase systems, for which UDP-responsive aptamers have been described. We present these not as demonstrated extensions of MCPS, but rather as examples of how the broader concept might eventually be explored outside nucleic acid catalysis. We believe these revisions better distinguish between what is directly demonstrated in the present work and the broader conceptual opportunities that it may open.

<u>Line 571-582 in the revised manuscript</u>

As noted, Figure 1B introduces "scavenged substrate" and "catalyst with one product," which are absent from the "ideal" cycle in 1A. To improve coherence, I suggest updating Figure 1A to show the intermediate state (catalyst bound to one product) after the first product dissociates, as this better reflects the actual mechanism described later in Figure 2.

Response: We thank the reviewer for this helpful suggestion. We agree that explicitly showing the intermediate state in which the catalyst remains bound to one product would improve the mechanistic continuity between Figure 1A, Figure 1B, and the more detailed reaction scheme in Figure 2. In response, we fully revised Figure 1 (from A to C) to include this intermediate after dissociation of the first product. We believe this change makes the catalytic cycle clearer and better aligns the conceptual overview in Figure 1 with the mechanistic description developed later in the manuscript.

<u>Line 114 in the revised manuscript</u>

Providing a "full cycle" diagram in the Supplementary Materials that tracks the exact fate of the catalyst in the presence of scavengers.

Response: We thank the reviewer for this helpful suggestion. In response, we added a full mechanistic cycle diagram in the SI (Figure S9) that explicitly tracks the fate of the ribozyme catalyst (and its complexed form with products or substrate) in the presence of scavenger. Rather than using a simplified schematic cycle, we chose to present the full reaction cycle so that the catalyst-bound intermediates, product-scavenging step, and substrate re-entry pathway are shown explicitly. We believe this more directly addresses the reviewer's request and clarifies the catalyst's progression through the scavenger-assisted turnover cycle.

Line 269-272 in the revised SI

In a real-world "ideal" reaction, the binding of a new substrate might actually help "push" the old product off the enzyme (associative mechanism). Does the scavenger-mediated displacement compete with or synergise with the natural substrate-assisted dissociation?

Response: We thank the reviewer for this insightful comment. We agree that binding a new substrate can sometimes assist the dissociation of the remaining product via an associative pathway. In fact, our mathematical model already includes both substrate-assisted removal of the old products and scavenger-mediated displacement steps (Supplementary note 3). The overall turnover therefore reflects the combined contributions of these parallel pathways, with the faster route dominating under a given condition. Also, it should be noted that the scavenger-mediated product displacement was much faster (~10^4 times) than the substrate-mediated product displacement in our model. We have clarified that these two reactions were considered together in the revised SI.

Line 229-231 in the revised SI

Line 303-306 in the revised manuscript

The Figure 1A "ideal" scheme should be updated to clarify if the "ideal" refers to zero inhibition or the specific MCPS mechanism.

Response: We thank the reviewer for this comment. Our intention in Figure 1A was to illustrate qualitatively the expected results of ideal product scavenging, defined as the case where product scavenging is rapid enough that product dissociation is no longer the rate-limiting step and when substrate scavenging does not occur. In such a case, conversion is limited by substrate binding and catalytic rate rather than MCPS explicitly. The goal of MCPS is to approach this idea case, but in practice it mitigates but not completely eliminates substrate scavenging. We now have revised Figure 1A with more detailed mechanism and revised its associated caption to clarify that the scheme represents the slowest product dissociation step is removed in the presence of scavenger that performs product scavenging.

<u>Line 114 in the revised manuscript</u>

Minor comments:

If excessive scavenger binding to the substrate is the bottleneck, could the authors comment on a "multi-layer" scavenging system? For example, using a small amount of "Primary Scavenger" and "Secondary Scavenger" that clears the primary scavenger from the product-scavenger complex. Would this allow for catalytic use of the scavenger itself?

Response: We thank the reviewer for this interesting suggestion. Conceptually, a multi-layer scavenging system could be envisioned in which a primary scavenger first captures the ribozyme-bound product and a secondary strand then displaces or removes the primary scavenger from the product–scavenger complex. However, such a system would not automatically make the scavenger catalytic. In the present MCPS design, product scavenging primarily functions as a stoichiometric sink, removing cleaved product from the ribozyme and thereby promoting turnover. If a secondary scavenger were used to regenerate the primary scavenger, the secondary scavenger would itself likely become the stoichiometric sink for the product-containing complex. Thus, the overall system would still require stoichiometric product capture. Moreover, implementing such a system would introduce additional design constraints. For example, depending on its sequence, the secondary

scavenger could interact with the ribozyme, substrate, or product-containing intermediates, potentially affecting substrate association, ribozyme folding, or productive product removal. Therefore, although multi-layer scavenging is an interesting conceptual direction, it would require a more complex hybridization and strand-transfer network than the one examined here. This is beyond the scope of the present study, whose goal was to establish the core MCPS concept, which solves the tradeoff between substrate and product scavenging via a physical mechanism.

Asymmetric Toeholds: Could adding shorter, reversible toeholds on the scavenger increase the rate of substrate transfer from the "scavenged substrate" back to the ribozyme?

Response: We thank the reviewer for this suggestion. We interpret the reviewer’s comment as referring to the possibility that altering the toehold architecture of the scavenger could improve transfer of substrate from a scavenger-bound state back to the ribozyme-containing complex. Because “shorter, reversible toeholds” could affect several coupled steps in this system, including substrate sequestration by the scavenger (substrate scavenging), scavenger-mediated product displacement (product scavenging), and substrate exchange with the ribozyme-bound product, this design parameter is not straightforward to predict a priori.

This issue is closely related to the point raised by Reviewer 1 regarding the role of the residual output toehold on the ribozyme-bound product (HHR-5’P). In response to that comment, we performed additional experiments testing six related scavenger variants with altered input and output toehold lengths, and these data are now included in revised Figure S6. These experiments directly examine whether changing the toehold architecture improves scavenger-mediated HHR turnover. The results showed that toehold architecture influences activity, but not in a simply predictable manner. Among equal-length pairs, the 3′-truncated variants, Sca-15-3′ and Sca-14-3′, showed higher turnover than the corresponding 5′-truncated variants, Sca-15 and Sca-14, consistent with the idea that the geometry of the remaining toehold can affect strand exchange. However, the improvement was modest, and shorter scavengers were not uniformly better than Sca-16. For example, Sca-14 was less effective than Sca-16, indicating that weakening scavenger–substrate interactions can compromise productive product scavenging.

We therefore agree that asymmetric toehold design is a relevant parameter, but our new data suggest that scavenger performance is governed by multiple coupled factors rather than by toehold reversibility or toehold length alone. We have revised Figure S6 and its caption to clarify this point and to present the new variant analysis.

<u>Line 209-219 in the revised SI</u>

Figure 2E Labeling: The placement of the "Scavenger" label on top of the thick red arrow in Figure 2E is slightly misleading, as it might be mistaken for a reaction condition rather than a physical species.

Response: We appreciate the reviewer's helpful comment. We have revised Figure 2E and Figure 2F to make it clear that "scavenger" denotes a nucleic acid species, not a reaction condition.

<u>Line 138 in the revised manuscript</u>

Reviewer #3

Reviewer overall comment:

This manuscript demonstrates a concept of multiphase-controlled product scavenging (MCPS), which combines the scavenger and LLPS concepts together. The help of a scavenger strand increases the catalytic turnover, and is further enhanced by co-localizing ribozymes and transcription templates within DNA nanostar-based LLPS condensates. The work of using scavengers to improve the catalytic turnover of ribozyme/DNAzyme is not new, just as the authors cite the work of Winfree in 2009. LLPS mediated by nanostars is also based on classic work from the Franco and Schulman labs. In this manuscript, the authors combine these two well-known concepts together to achieve a programmable framework to manipulate the ribozyme catalytic capacity. I think the concept is quite intriguing. Although the two concepts are not new, coupling them together leads to a novel idea to engineer ribozyme efficiency. That's why I think this work is noteworthy for publication in Nature Communications. However, I would recommend the authors to improve the structure of the manuscript to present this concept in a more rigorous way before the manuscript is accepted.

Overall response:

We thank the reviewer for this thoughtful and positive assessment. We agree that both toehold-mediated strand displacement and nanostar-based LLPS are well-established techniques with a range of applications. However, we would like to clarify the distinction between those enabling concepts and the specific advances of the present study. The prior strand-displacement literature cited by the reviewer, including Zhang and Winfree (2009), established the kinetics and programmability of enzyme-free toehold exchange. Although strand displacement chemistry itself is well established, it is not obvious from that mechanism alone that a product-scavenging strategy would improve ribozyme or DNAzyme turnover in practice because the scavenger can also bind the highly similar uncleaved substrate, perturb catalyst-product or catalyst-substrate exchange, or introduce new rate-limiting steps that offset any benefit from product removal. Moreover, RNA:DNA hybrid strand displacement is strongly sequence-dependent, and the relevant exchange occurs in the context of a folded ribozyme-product complex, where tertiary structure, local accessibility, and coupling to the catalytic core could frustrate an otherwise plausible scavenger design. The advance here 1) identifies product removal as the key rate limiting step in RNA

cleavage, identifies designs and thermodynamic driving forces and putative kinetic rates that would alter the rate-limiting step of a cycle of enzymatic cleavage involving multiple reactions, and creating a coherent design strategy for scavengers that sidestep potential biochemical and structural limitations to the reaction rates. We further show how these effects are relevant whether cleavage or ligation are favored and work generally for different RNA enzymes with different structures. This assessment concurs with the assessment of Reviewer 1, who finds this work of great interest for the design and use of scavengers to control reaction cycles as well as Reviewer 2's enthusiasm for the scavenger development.

We appreciate the reviewer's kind suggestion about our group's role in the establishment of LLPS mediated by nanostars. While our group has published at least one other result using DNA nanostars (J. Am. Chem. Soc. 2024), credit for the development of LLPS by DNA nanostars would more properly be given to Francesco Scortino (ACS Nano 2017 and PNAS 2013) and Omar Saleh (J. Chem. Phys. 2022 and Soft Matter 2018) for seminal early work in this area, and also to the Franco group for a range of papers about the use of nanostar LLPS for cargo sequestration (*e.g.* Nat. Nanotech. 2024) and control over LLPS using strand displacement (Nat. Commun. 2024). In this work, we used well-understood principles of LLPS by nanostars and created mechanisms to ensure robust co-partitioning of multiple species that build on the approaches of other groups. The key advance, however, is to show how this co-partitioning can further enhance turnover acceleration by explicitly controlling the balance of rates through competing reactions, exploiting the different local concentrations of species within different phases.

We appreciate the reviewer's consideration of this work and further the consideration that combining multiple mechanisms to achieve a new overall effect is an advance worthy of study and that such an advance is suitable for *Nature Communications*. We agree with the reviewer combination of multiple interactions and mechanisms to produce functional and robust chemical systems is a critical topic for research in chemistry and chemical and biomolecular engineering and will be required to achieve effects not possible through single mechanisms. Yet the combination and how they work best is often non-obvious.

We appreciate the many thoughtful questions and comments mentioned throughput this review and respond them point by point below. We appreciate the opportunities these questions and comments have presented to improve the manuscript.

Major comments:

1. Generalizability of scavenger designs

In the manuscript, the design principle of generalizable scavengers was not elaborated clearly. The authors mention that they used NUPACK to help with the sequence design, but the criteria or design rules used to achieve efficient scavengers were not clearly described. For readers who would like to cite this manuscript and design scavengers for other types of ribozyme or DNAzyme, what rules should they follow? The figures and main text do not mention this clearly. It is somewhat difficult to infer the base-pairing regions directly from Figures 3C and 3D.

Response: We thank the reviewer for this helpful opportunity to improve the manuscript. In the revised manuscript, we describe the three requirements for a scavenger: (i) the scavenger should preferentially displace and stably sequester one cleavage product (for instance, 3' end product (3'P) in hammerhead ribozyme (HHR) reaction in our paper), (ii) the scavenger-mediated product removal should be faster than spontaneous substrate-mediated product dissociation, and (iii) the full-length substrate should remain capable of forming the substrate-ribozyme complex for continued catalysis in the presence of unreleased product (5' end product; 5'P), ribozyme, full-length substrate, and excess scavenger.

We further clarify that, in our tested nucleic acid enzyme systems (Hammerhead ribozyme, hairpin ribozyme, and 10-23 DNAzyme), a design methodology that satisfies these three requirements is to choose a scavenger that: (1) binds the target product—3'P in hammerhead ribozyme and hairpin ribozyme reactions, and 5'P in 10-23 DNAzyme reactions—more strongly than the target binds to its cognate nucleic acid enzyme; (2) provides a kinetic pathway to displace the target product at a rate much faster than spontaneous substrate-mediated pushing of products to remove canonical rate-limiting step, estimated from toehold-mediated strand-exchange kinetics; and (3) thermodynamically favors substrate-ribozyme binding over scavenger-substrate binding in the

presence of 1X, 10X, and 100X scavengers. Thermodynamically, the 1st and 3rd requirements were satisfied in NUPACK models. Kinetically, because NUPACK does not prescribe or predict a kinetic pathway for strand exchange, we separately evaluated whether each scavenger candidate could initiate product displacement through an accessible toehold expected to support toehold-mediated strand exchange. For example, in the HHR system, the apparent rate constant for spontaneous formation of a new substrate–ribozyme complex through substrate-mediated replacement of the old product was only 6.8 $M^{-1}sec^{-1}$. Therefore, we designed scavengers to initiate displacement of HHR-bound 3′P through a 3′P toehold longer than 4 nt, which is expected from established toehold-mediated strand-exchange kinetics to be substantially faster (>1,000-fold) than the spontaneous substrate-mediated pathway. The revised Supplementary Note 2 and main text now clearly state these points.

To address the reviewer's point regarding figure readability, we have revised Figures 3C and 3D to indicate the scavenger-binding site, which provides an anchor point for readily identifying specific base-pairing regions.

Line 244-260, and 315 in the revised manuscript

Line 139-170 in the revised SI

2. Generalizability of MCPS

In Figures 2 and 3, the authors provide examples of scavengers for the hammerhead ribozyme, the 10–23 DNAzyme, and the hairpin ribozyme, demonstrating that the scavenger strategy can function across different types of nucleic acid enzymes. However, for the central concept of this work,MCPS, the manuscript currently presents only one example using the hammerhead ribozyme.

Response: We thank the reviewer for this comment. We agree that, in principle, additional experiments investigating how multiphase-controlled product scavenging (MCPS) could enhance turnover could broaden the scope of the study. In the revised manuscript, as described below, we have provided additional evidence to show how partitioning the template and ribozyme can bias

the binding of a substrate to a partitioned partner, such as a ribozyme, over a binding partner in the bulk, and that this preference is not specific to the HHR ribozyme.

We focused our efforts in the original manuscript on investigating how partitioning of the template and ribozyme in themselves on the HHR ribozyme because analyzing the products of a full MCPS experiment is technically much more complex than quantifying cleavage products in a reaction involving scavenging in a well-mixed solution. In the well-mixed experiments, shown in Figures 2 and 3, cleavage can be quantified directly from a labeled synthetic substrate by denaturing gel electrophoresis because the substrate is synthesized previously and purified so that it forms a clean band on a gel. In contrast, in the MCPS experiment, the substrate is generated continuously by transcription in a mixture containing DNA nanostars, transcription templates, ribozymes, RNAP, scavengers, transcripts, and cleavage products; conventional gel staining cannot specifically distinguish the ribozyme-generated product from the many other nucleic acid species present. We therefore had to develop a product-specific downstream ligation/fluorescent labeling assay to quantify HHR cleavage products under multiphase transcription-coupled conditions. This assay exploits the difference in end-group chemistry of the cleaved product from uncleaved transcripts and chemically synthesized oligonucleotides. Importantly, as a result, this type of product-specific labeling platform is not generally transferable from one nucleic acid enzyme to another: each ribozyme or DNAzyme produces products with different sequences, termini, structures, and chemical features, so a new enzyme system would require a newly designed and validated product-specific readout. This would require substantial synthesis of long and/or fluorescently modified oligonucleotides and extensive optimization.

Thus, to address the reviewer's concern directly yet without undue delay and expense, we added Figure S24, which experimentally demonstrates the central mechanistic principle of MCPS—multiphase-assisted reaction pathway canalization—using a DNA reporter system. Briefly, in this assay, the transcribed HHR substrate can bind either of two competing reporter complexes with no inherent thermodynamic preference, and binding is monitored through displacement of a fluorophore-labeled strand. In the multiphase system, the newly transcribed substrate preferentially binds the co-localized reporter rather than the dispersed reporter, whereas this preference is lost in the corresponding single-phase control. This principle illustrates that the central mechanism underlying the production speedup in the HHR case is maintained for a

completely different set of competing binding partners. Figure S24 thus provides a direct experimental validation that compartmentalization biases substrate binding toward the intended pathway, and that this bias is not specific to the HHR but rather a general property of multiphase systems that could be exploited generally.

The revised manuscript and SI therefore first show that product scavenging is applicable to multiple nucleic acid enzymes, and, by adding Figure S24 with revised manuscript and SI to directly, demonstrate the core physical principle underlying MCPS and how it is general. We believe this clarification makes clear both why only one full MCPS implementation is presented experimentally at this stage and why the underlying principle is expected to be more broadly applicable.

Line 444-458 in the revised SI

Line 508-522 in the revised manuscript

Figure 4c suggests two possible strategies to generate LLPS, either by co-partitioning the nanostar with HHR or with the DNA template of the RNA substrate. However, it appears that only one of these approaches was used in Figure 5C. This point would benefit from clarification. It would be helpful to know whether the authors also tested the alternative strategy and, if so, what the outcome was.

Response: We thank the reviewer for this comment as it suggests we could have been more clear. Critically, a key result is that we need to co-partition *both* the HHR (or in general, the ribozyme) and the DNA template from which the RNA substrate is transcribed. Both are used in Figure 5C (i.e. for MCPS). The reviewer may be referring to the diagram in Figure 4C, where in the original manuscript we showed that a similar strategy could be used to co-partition both the HHR and the DNA template within DNA nanostar condensates. Here, one of the three sticky ends of DNA nanostar was extended with a binder domain capable of hybridizing with a target strand. For ribozyme localization, this domain contained a 15-nt sequence complementary to the 3' extended end of HHR. For template duplex localization, this domain was modified to include a T7 promoter

and a sequence encoding HHR Sub, which forms a stable duplex with a non-template sequence. For clarity, we revised Figure 4C to include such information

Importantly, we did test the “alternative” strategies in which only one component was localized. As shown in Figure 5A-B, we compared all four cases: (1) co-partitioned ribozyme and template (R-P, T-P), (2) partitioned template with dispersed ribozyme (R-D, T-P), (3) partitioned ribozyme with dispersed template (R-P, T-D), and (4) both dispersed (R-D, T-D). Only the co-partitioned condition accelerated the reaction, whereas localizing only the template or only the ribozyme gave results comparable to the fully dispersed control. Figure 5C was intended as a summary comparison rather than a separate test of only one localization strategy. We believe this confusion arose because the abbreviations describing the four localization conditions (R-P, R-D, T-P, and T-D) were defined in Figure 5B but were not shown in Figure 5A, although Figure 5A schematically presents the same four experimental configurations. We have now revised Figure 5A (and its caption) to include these abbreviations explicitly, so that the relationship between the schematics in Figure 5A and the results in Figure 5B is immediately clear.

<u>Line 453, and 457-458 in the revised manuscript</u>

While the concept of a “generalizable and programmable framework, multiphase-controlled product scavenging (MCPS)” is appealing, further clarification or additional examples would help readers better appreciate the scope and generalizability of this approach.

Response: We thank the reviewer for this helpful comment. We agree that clarifying the scope and generalizability of MCPS is important, and we have revised the manuscript to make the modular logic of the framework clearer. MCPS contains two mechanistic components: product scavenging and multiphase pathway canalization. First, we show that the product-scavenging component is general across multiple nucleic acid enzymes. In addition to the hammerhead ribozyme, we designed cognate scavengers for the 10–23 DNAzyme and the hairpin ribozyme, two catalysts that differ in sequence, structure, nucleic-acid composition, and cleavage/ligation behavior. In both cases, the scavenger enhanced catalytic turnover, supporting the conclusion that scavenger-mediated mitigation of product inhibition is not specific to the HHR architecture.

Second, we strengthened the evidence that the multiphase component of MCPS is also based on a general physical mechanism rather than an idiosyncratic feature of one ribozyme. In the revised manuscript, we added reporter experiments that directly test whether phase separation can bias molecular recognition toward co-partitioned partners. In these experiments, a newly transcribed HHR substrate preferentially reacted with a co-partitioned HHR-mimicking reporter rather than a dispersed scavenger-mimicking reporter in the multiphase system, whereas this preference disappeared in the corresponding single-phase control. This experiment demonstrates that compartmentalization can canalize nucleic-acid hybridization pathways by increasing the probability of reaction with spatially co-localized components, which is the core physical principle underlying the multiphase portion of MCPS.

We therefore now present MCPS as a programmable framework supported by two forms of generality: programmable scavenger design through nucleic-acid base pairing, demonstrated across three nucleic acid enzymes, and programmable pathway biasing through selective partitioning, demonstrated using a reaction-pathway reporter assay. We also clarified in the Discussion that the strongest current evidence for MCPS generality is within nucleic-acid systems, where both scavenger design and phase-localization strategies can be rationally programmed. At the same time, we note that the broader principle—combining product-selective affinity reagents with spatial control of competing molecular encounters—may guide future extensions to other catalytic systems where suitable product-binding reagents are available.

<u>Line 571-582 in the revised manuscript</u>

<u>Line 508-522 in the revised manuscript</u>

3. Some important details for Fig 5c need clarification

The most exciting experiment, in my opinion, is Figure 5c. However, I did not clearly understand how the ligation reactions were quantified and how the authors arrived at their conclusion. Maybe the authors have described this somewhere but I did not catch it.

(1) I'm convinced that DNA template of RNA substrate co-partition with LLPS of nanostars. However, each DNA templates can generate multiple copies of RNA substrates. Is there any way to show that all RNA substrates co-partition in the condensate? Though ref 49 is beautiful, Figure S19 is yet not obvious evidence. It is unclear that all of RNA copies co-partitioned in the droplets. Any the fraction of RNA that diffuse freely in and out the dilute phase? If so, how can we confidently quantify the yield of RNA cleavage reactions?

Response: We thank the reviewer for these important questions. The RNA substrates are generated from localized DNA templates within the condensate rather than being pre-partitioned species. This generation, critically, biases interaction, because the concentration of ribozyme in the condensates is much higher than the concentration of scavenger, meaning the RNA, tends to interact with a ribozyme before a substrate. In line with this key point, Figure S19 does not show partitioning of all RNA substrate copies into the condensates but rather demonstrates co-partitioning of the hammerhead ribozyme and the DNA template, which enables spatially localized RNA production within the condensates consistent with prior studies (*J. Am. Chem. Soc.* **146**, 32942–32952 (2024)).

Regarding the quantification of RNA cleavage, our measurement does not rely on the assumption that all RNA substrates remain confined within the condensates. Instead, at defined time points, we added a complementary DNA strand to hammerhead ribozyme to form a dead-end duplex, and performed heat treatment to both denature T7 RNA polymerase and dissolve DNA condensates. We then specifically detected the 5' end product by chemical ligation of a FAM-labeled ligator primer to the 2'-3' cyclic phosphate generated by hammerhead ribozyme, a chemical feature not present in full-length transcripts or solid-phase-synthesized RNA molecules. The ligation product was confirmed by denaturing gel electrophoresis. Therefore, the reported cleavage yield reflects the amount of hammerhead cleavage product formed, regardless of whether some RNA molecules exchanged with the dilute bulk phase during the reaction (please see the response of the below question for more detailed information about quantitative analysis).

(2) Please provide reference or experiment to validate the efficiency of ligation reaction in step 3 of Figure S20, since this step is the key readout. If 2',3' cyclic phosphate is hydrolyzed to form

either 2’ or 3’ mono phosphate, will the reaction still happen? (I think it is unlikely.) The hydrolysis of 2’,3’ cyclic phosphate might happen quite fast during heat treatment in step 2 of Figure S20.

(3) The increase in reaction is presented in Figure 5C, which appears better than single-phase product scavenging. However, this figure only presents the increase in fraction of downstream ligator reacted, but not the turnover or reaction rate. The relevant numbers are hard to find in figure legend or main text regarding the kinetic shift and the concentration of scavenger.

Response: We sincerely appreciate the reviewer’s helpful comments. To address them, we added Supplementary note 5 and Figure S26, which provide a quantitative calibration and analysis of the downstream ligation assay. Importantly, we do not assume that the ligation step in Figure S21 proceeds with 100% efficiency. Instead, the ligation readout was calibrated empirically under the same workflow used for the experimental samples. Specifically, defined concentrations of transcribed HHR substrate were fully cleaved using excess HHR, then subjected to the same quenching procedure used in the assay—addition of c(HHR) followed by 90 °C for 5 min heat treatment and cooling—before addition of ligator substrate (Ligator Sub) and ligation-performing ribozyme (Ligator Ribo). The resulting relationship between the fraction of ligator reacted and the concentration of cleaved transcripts was then used to quantify product formation in Figure 5.

We agree with the reviewer that if the 2’,3’-cyclic phosphate were hydrolyzed to a 2’- or 3’-monophosphate, efficient ligations by this readout would not be expected. However, because the calibration samples and the experimental samples underwent the same post-cleavage treatment prior to ligation, any loss of ligatable cyclic phosphate during the quenching step would be reflected in the calibration curve itself. Thus, our quantification does not rely on assuming complete preservation of the cyclic phosphate during heating; rather, it relies on an experimentally calibrated conversion from ligation signal to cleaved-product concentration under the exact assay conditions used.

To address the reviewer’s second point, we now report the quantitative kinetic values corresponding to Figure 5C more explicitly. The experiments in Figure 5 were performed with 10 μM Sca-16. Using the calibrated ligation assay and a simple transcription-coupled kinetic model, we estimated that after 2 h the MCPS condition produced 3213 nM cleaved products, whereas the corresponding single-phase product scavenging condition produced 1730 nM cleaved products.

The fitted apparent rate constants were 7.9×10^{-2} min^{-1} for MCPS and 1.2×10^{-2} min^{-1} for single-phase product scavenging, corresponding to a 6.5-fold enhancement in cleavage rate. These values are now presented in the revised main text and supported by revised SI (with Supplementary note 5).

Line 547-556 in the revised manuscript

Line 471-511 in the revised SI

(4) Importantly, please include the raw gels of figures 5B and 5C in the SI. This will convince the readers of the conclusions.

Response: We thank the reviewer for this helpful suggestion. We have now included the raw gel images for Fig. 5B and 5C in the SI as Figure S21. We agree that providing these raw data improves transparency and further supports the conclusions drawn from these experiments. Moreover, most raw data used in the manuscript is available at: https://github.com/BH-Kang/Scavenger-project and all data will be provided as a DOI archive.

Line 425-430 in the revised SI

4. Structure of the manuscript

The author spent a large portion of manuscript establishing of scavenger concept, including the modeling and kinetic analysis in figure 2 and 3. While readers may appreciate this is as a foundation before moving towards MCPS, this concept itself is not new. I'm not quite sure whether this part helps the readers better appreciate this work or instead dilute the importance of the key point of this manuscript. In my understanding, the novelty and excitement of this manuscript lie mainly in figure 5. With all above three major issues, I would recommend to adjust the manuscript to include more data and discussion showcasing the programmability and generalizability of MCPS.

Response: We thank the reviewer for this thoughtful comment. In our view, programmable product scavenging is itself a central conceptual contribution of this work, because it establishes a new strategy to relieve product inhibition in nucleic acid catalysis and defines the key tradeoff with substrate scavenging that motivates MCPS. This aspect of the work was also independently recognized by other reviewers, who described the scavenger principle as novel, widely interesting, and exciting. For this reason, we believe that Figures 2–3 are not merely background, but provide a key contribution. Figures 4–5 then build on this foundation by showing how multiphase spatial control further enhances catalytic flux by overcoming the remaining limitation of scavenging alone. In response to the reviewer's thoughtful comments above, we have also strengthened the manuscript with one more example for reaction pathway canalization (Figure S24) and additional quantitative analysis (Figure S26) to understand how much the cleavage rate could be accelerated which provide more results about how MCPS can accelerate multiple catalytic turnovers.

Minor comments:

1. With the scavenger, the mechanism of the dynamic circuit is different from equilibrium-shifting reaction? If the scavenger-mediated displacement changes microscopic rate constants or merely shifts effective equilibria? Could you make it clearer?

Response: We thank the reviewer for this question. The scavenger is not intended to change the intrinsic microscopic rate constants of the catalytic cleavage/ligation step itself (we did not change rate constants in the simulation model when adding scavengers; Supplementary Note 3). Rather, it introduces additional strand-exchange reactions, including product capture and substrate scavenging, that alter the occupancies of intermediate states and reroute the turnover pathway. Thus, scavenging is not simply an equilibrium shift within the original reaction scheme, but a kinetic-network rewiring that changes the effective post-cleavage landscape while leaving the core catalytic chemistry unchanged. We have clarified this point in the revised manuscript.

<u>Line 294-296 in the revised manuscript</u>

2. The kinetic modeling contains some assumed or fitted parameters, such as the substrate transfer from scavenger to ribozyme-product complex. Could you provide some experiments to explicitly report parameter ranges tested? Or confidence intervals for fitted rates?

Response: We thank the reviewer for this helpful comment. In the revised manuscript, we have strengthened the treatment of the fitted parameters (k_{tot} and k_{trf}) by explicitly reporting their statistical uncertainty. Whereas the original version used averaged experimental values (obtained from ribozyme-mediated cleavage reactions) for fitting, the revised analysis fits the experimental measurements directly and reports the 95% confidence interval for the fitted rate constant. Briefly, the kinetic model was implemented as a system of ODEs describing concentrations of each molecular species over time. For each trial value of k_{tot} or $k_{tot,2}$, the ODE was solved numerically using odeint, and the simulated observable ([5' end product]) was compared with the experimental data after interpolation to the measured time points. The best-fit was obtained by nonlinear least-squares optimization, with k_{tot} or $k_{tot,2}$ constrained to non-negative values and the other rate constants fixed (the code is now uploaded here: https://github.com/BH-Kang/Scavenger-project). To quantify uncertainty, we calculated 95% confidence interval for each fitted parameter using the standard error supplied by the Jacobian-based covariance matrix of the least-square fit. The revised manuscript and SI now report the fitted kinetic parameters together with their 95% confidence intervals.

<u>Line 204 and 290 in the revised manuscript</u>

<u>Line 325 in the revised SI</u>

3. This paper discusses a lot about the tradeoff between substrate scavenging and product scavenging. Do you have any direct quantitative measurements of substrate-scavenger binding affinity?

Response: We thank the reviewer for this question. We did not perform a separate direct binding assay between nucleic acid strands, including substrate-scavenger binding. In this study, the relative affinity of the substrate for the scavenger was evaluated using standard nucleic-acid design/thermodynamic analysis based on sequence complementarity (for example, NUPACK-

based calculations; see Fig. S5 and Table S2), together with the concentration-dependent kinetic behavior of the system. The kinetic rates used in the model were taken from previous studies (*Nucleic Acids Res*. **50**, 9647–9662 (2022), *ACS Synth. Biol*. **10**, 3066–3073 (2021), and *Nanoscale* **16**, 17624–17637 (2024)) or constrained from our experimental data, rather than introduced arbitrarily. This type of affinity estimation is commonly used in nucleic-acid circuit design when many different base-pairing interactions must be considered simultaneously. Also, this is one of the unique strengths of DNA design. In addition, under our experimental conditions the HHR–substrate interaction is very strong and effectively irreversible on the timescale of the assay, making direct quantitative measurement of a competing substrate–scavenger equilibrium less straightforward and less informative than relative thermodynamic/kinetic analysis. We have clarified this point in the revised SI (Supplementary Note 3).

<u>Line 255-260 in the revised SI</u>

4. Fig.1 could much clearer labeling of rate-limiting steps.

Response: We thank the reviewer for the comment. We revised Figure 1A, 1B and 1C in the manuscript.

<u>Line 114 in the revised manuscript</u>

5. Fig. 3A needs the annotation for lines. Fig. 3B is hard to see all conditions mentioned in the annotation.

Response: We thank the reviewer for this helpful comment. We have revised Figure 3 to improve clarity. Specifically, we added annotations identifying the lines and dot points in Figure 3A, and we enlarged the annotations in Figure 3B to improve readability.

<u>Line 315 in the revised manuscript</u>

6. Fig.5 should show the conditions or annotations in the graphs.

Response: We thank the reviewer for this helpful comment. We have revised Figure 5. In detail, Figure 5A now includes the abbreviated terms used for the experimental groups in Figure 5B; Figure 5B now indicates the experimental conditions common to all groups (500 nM HHR, 100 nM template, and 10 µM Sca-16); and Figure 5C now explicitly includes the label "MCPS".

Line 453 in the revised manuscript

7. Some data not specified with numbers of replicates. Please clarify n values consistently.

Response: We thank the reviewer for this comment. We have fully revised the manuscript and SI to ensure the number of replicates (n) for all experiments is specified.

8. What's the concentration of scavenger to the HHR and HHR sub? The manuscript does not always mention. It would be helpful to include this information in the figure captions. (For instance, I was wondering about this when I read figure 2, but I had to dig into the SI and found the scavenger concetration is 20 uM, if I understood correctly.)

Response: We appreciate the reviewer's helpful comment. We agree that the concentrations of scavenger, nucleic acid enzyme, and its substrate are important to know explicitly. We have revised the captions of Figures 2 and 3 to include the concentrations of the scavenger, nucleic acid enzyme, and substrate used in each experiment.

Line 145-146, 158-159, 318, 327-328, 331, and 333 in the revised manuscript

Reviewer #4

Reviewer overall comment:

I co-reviewed this manuscript with one of the reviewers who provided the listed reports. This is part of the Nature Communications initiative to facilitate training in peer review and to provide appropriate recognition for Early Career Researchers who co-review manuscripts.

Overall response: We thank the reviewer for their careful and constructive comments. Our responses to each point are provided above.

Point-by-Point Response

Manuscript #: NCOMMS-26-007007A

Title: Dramatic reaction enhancement via product scavenging and phase separation-driven pathway canalization

Corresponding Author: Dr. Rebecca Schulman

Authors: Byunghwa Kang *et al*.

Reviewer #1

Reviewer overall comment:

The authors have substantially revised and improved the manuscript and I think it is well on the way to acceptance. A couple of issues remain incompletely addressed, however

Overall response: We sincerely appreciate the reviewer's positive assessment of the revised manuscript and for acknowledging the substantial improvements made. We also appreciate the reviewer's identification of the remaining issues. We have carefully addressed each of these points in the revised manuscript, as detailed in our point-by-point responses below.

Comments:

1. The figures are still not publication quality; the text and lines that should be very sharp are clearly blurred when one zooms in. I leave this as something to be addressed when the proofs are prepared.

Response: We thank the reviewer for noting this issue. To ensure publication-quality presentation, we updated our manuscript with vector images, also we will upload each figure separately as high-resolution files generated directly from the original source.

2. The model on lines 92-99 of the SI still wouldn't behave in a biphasic way, I think. You can still just add the pathways together in the ODEs (this time yielding a single process with a weighted average rate). To get a biphasic behaviour, one would need to explicitly represent the two variants of the ribozyme (in all its complexes) as separate variables -- eg. [ScR1] and [ScR2].

Response: We thank the reviewer for this careful observation. We agree that, because the weighted fast and slow cleavage terms in our original equations act on the same substrate–ribozyme complex, they can be combined mathematically into a single effective cleavage process. Therefore, the original turnover model itself does not reproduce the biphasic single-turnover kinetics shown in Fig. S1. Our intention was to use the biphasic fit only to obtain an ensemble-averaged description of cleavage and ligation for the subsequent multiple-turnover model. The model was designed to evaluate how product release and substrate transfer affect catalytic turnover, rather than to identify the molecular origin of the biphasic single-turnover behavior.

We considered explicitly representing fast and slow ribozyme populations and all of their corresponding complexes as separate variables. However, such a formulation would require the additional assumption that the fast and slow kinetic modes correspond to persistent, noninterconverting molecular populations throughout the entire reaction cycle. The current ensemble kinetic data does not establish this assumption or provide sufficient constraints to independently parameterize all of these additional states. A biphasic time course indicates that two kinetic components are needed to describe the experimental curve, but it does not demonstrate that individual ribozyme molecules remain permanently assigned to fast or slow populations.

We have therefore revised Supplementary note 1 to clarify the scope and limitations of the model. Specifically, we now express the weighted cleavage and ligation terms as effective ensemble-averaged rate constants and explicitly state that the coarse-grained model is not intended to reproduce the biphasic single-turnover time course. Also, we edited our main text to avoid term "biphasic behavior". This revision does not affect the turnover simulations or the conclusions regarding the rate-limiting effects of product release and substrate transfer.

<u>Line 103-110 in the revised SI</u>

Line 183 in the revised manuscript

3. The authors have indeed now cited work on templated ligation/assembly (not both the ones that I recommended, as I understood from the rebuttal, but one of those and some alternatives). But the key point is that they haven't fully engaged with the differences from their work, which is a shame.

(i) In line 89-93, where the citation is made, the authors aren't really clear enough that they are drawing a contrast with ligation/assembly and their work. Perhaps simply extending the "severe" to "even more severe than for cleavage reactions" is enough to make this point.

Response: We thank the reviewer for the comment. As the reviewer suggested, we extended the “severe” to “even more severe than for cleavage reactions”.

Line 91-92 in the revised manuscript

(ii) The authors really should engage with the Ref 28 properly in the Discussion with regard to its demonstration of a scavenger-like motif. There are relative strengths and weaknesses of both papers, and understanding them is very helpful in thinking about where this work could go next. In particular, a major weakness of the manuscript under consideration is that scavengers bind to substrates - an advantage of Ref. 28 is that the equivalent to the scavenger only binds to products, because the assembled product presents a very different target from the reactants. How to generalize such a strategy to cleavage is not immediately obvious, but exploring whether it is possible is surely of interest and worth discussing?

Response: We thank the reviewer for this helpful suggestion. We agree that Ref. 28 provides an important complementary strategy in which product assembly generates a new recognition motif, allowing the fuel strand to act preferentially on the completed product and thereby avoid reactant sequestration. We have added a concise paragraph to the Discussion comparing this advantage with the substrate-scavenging limitation of our cleavage system and highlighting product-gated scavenging based on cleavage-generated termini or structures as an important future direction.

<u>Line 603-612 in the revised manuscript</u>

Reviewer #2

Reviewer overall comment:

I am satisfied with the authors' response and the revisions made to the manuscript. Therefore, I recommend it be accepted for publication.

Overall response: We sincerely thank the reviewer for their positive assessment of our responses and the revised manuscript. We greatly appreciate the reviewer's time, thoughtful evaluation, and recommendation for publication.

Reviewer #3

Reviewer overall comment:

I appreciate the considerable effort the authors have made in revising the manuscript. The revised version is much improved. The authors have addressed all major and minor concerns in my previous review. The additional data and analyses have strengthened the manuscript. The figures are also clearer. I continue to find the central concept interesting and believe this work represents a useful conceptual advance to mitigate product inhibition in nucleic acid catalysis. I would love to see this work published soon.

Overall response:

We sincerely thank the reviewer for their positive and encouraging assessment of the revised manuscript. We greatly appreciate the reviewer's thoughtful comments and recognition of the additional data, analyses, and improved figures. We are also grateful for the reviewer's view that the central concept represents a useful advance in mitigating product inhibition in nucleic acid catalysis. The reviewer's constructive feedback throughout the review process has substantially helped us strengthen the manuscript.

Comments:

I only have two minor comments, which may simply reflect my personal writing preference.

1. Although the manuscript is clearer than before, I still found parts of it more difficult to follow than necessary, and some sections could be a bit wordy. In several places, lengthy conceptual ideas are introduced before the experiment is clearly described, making the flow less straightforward. I think the manuscript would benefit from a more streamlined presentation. A very minor point: I would also avoid repeating the same transition words in adjacent sentences. For example, line 239-241, both sentences begin with "Thus…" ; line 400-402, both sentences begin with "Because….”

Response: To identify sections that were unnecessarily wordy or difficult to follow, we discussed the manuscript with several colleagues, who are potential readers of the work, and carefully revised multiple paragraphs throughout the text. In particular, we shortened lengthy conceptual explanations, introduced experimental aims and procedures more directly, simplified sentence structure, and removed redundant wording. We also corrected the repeated transition words noted by the reviewer and reviewed the manuscript for similar instances. We believe these revisions have made the presentation more concise and the overall flow easier to follow.

Line 165, 172-174 in the revised manuscript

Line 238-240 in the revised manuscript

Line 243-263 in the revised manuscript

Line 374-378 in the revised manuscript

Line 387-389 in the revised manuscript

Line 455-465 in the revised manuscript

2. I encourage the authors to be slightly more conservative when discussing the generality of the approach. The revised manuscript now demonstrates the concept across three different nucleic acid

enzymes, which strengthens the work. However, it remains unclear whether the same strategy can be broadly applied to other catalytic systems.

Response: We thank the reviewer for this important comment. Although the successful application of product scavenging to three distinct nucleic acid enzymes demonstrates generalizability among the systems examined, it does not establish broad applicability across all nucleic acid catalysts or other catalytic classes. We have therefore revised the Discussion to distinguish clearly between the scope demonstrated experimentally and possible future extensions. Specifically, we replaced statements that MCPS is "readily generalizable" with more conservative language indicating its potential broader applicability within nucleic acid catalysis. In addition, we removed the sentence proposing aptamers against small peptide products of proteases, because the feasibility of obtaining such aptamers has not been established.

Line 551-563 in the revised manuscript

Reviewer #4

Reviewer overall comment:

I co-reviewed this manuscript with one of the reviewers who provided the listed reports. This is part of the Nature Communications initiative to facilitate training in peer review and to provide appropriate recognition for Early Career Researchers who co-review manuscripts.

Overall response: We truly appreciate the reviewer for constructive and helpful comments. Our responses to reviewers' comments are provided above.
